# Fluconazole-COX Inhibitor Hybrids: A Dual-Acting Class of Antifungal Azoles

**DOI:** 10.1021/acs.jmedchem.1c01807

**Published:** 2022-01-27

**Authors:** Rebecca Elias, Pallabita Basu, Micha Fridman

**Affiliations:** School of Chemistry, Raymond & Beverly Sackler Faculty of Exact Sciences, Tel Aviv University, Tel Aviv 6997801, Israel

## Abstract

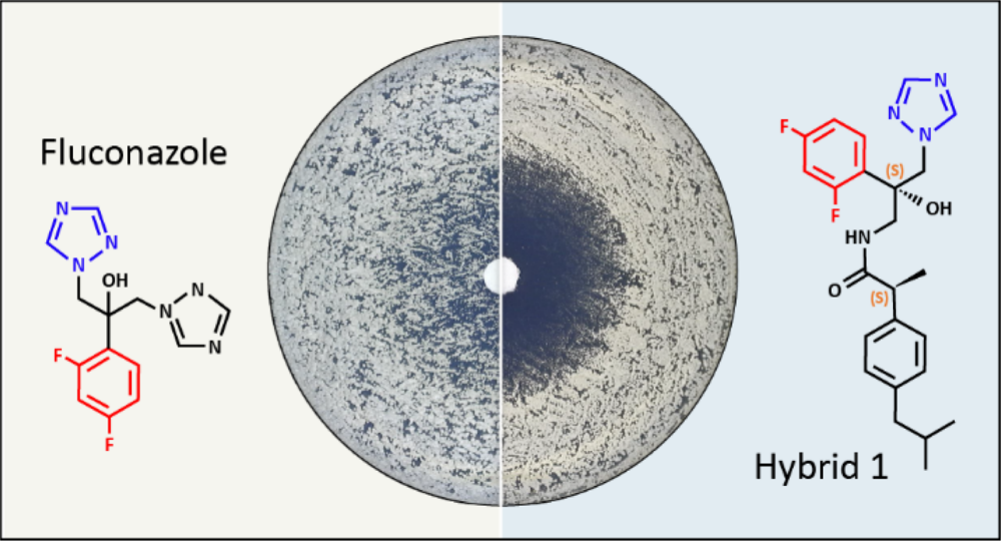

When used in combination with azole antifungal drugs, cyclooxygenase (COX) inhibitors such as ibuprofen improve antifungal efficacy. We report the conjugation of a chiral antifungal azole pharmacophore to COX inhibitors and the evaluation of activity of 24 hybrids. Hybrids derived from ibuprofen and flurbiprofen were considerably more potent than fluconazole and comparable to voriconazole against a panel of *Candida* species. The potencies of hybrids composed of an *S*-configured azole pharmacophore were higher than those with an *R*-configured pharmacophore. Tolerance, defined as the ability of a subpopulation of cells to grow in the presence of the drug, to the hybrids was lower than to fluconazole and voriconazole. The hybrids were active against a mutant lacking CYP51, the target of azole drugs, indicating that these agents act via a dual mode of action. This study established that azole-COX inhibitor hybrids are a novel class of potent antifungals with clinical potential.

## Introduction

Although humans and yeast have been evolving along different paths over a period of about a billion years, there is still a significant resemblance between the genomes of human and both friendly and pathogenic yeast.^1−5^ Approximately one-third of the genes found in the human genome have counterparts in the genomes of yeast; amino acid sequences of the human proteome overlap by more than 30% with those of the yeast proteome.^6^ Moreover, when 414 human genes were inserted into yeast cells one at a time, approximately 50% of them were found to be functional and facilitated the survival of the yeast cells.^7^ It is, therefore, no wonder that, compared to the relative abundance of unique drug targets in bacteria, few such targets are suitable for selective inhibition of essential cellular processes in pathogenic fungi.

Prevention and treatment of fungal infections currently relies on a relatively limited number of antifungal drugs in only four major drug classes: azoles, echinocandins, allylamines, and polyenes.^8−10^ The incidence of fungal infections has risen sharply in recent decades due to growing numbers of immunosuppressed persons and higher prevalence of drug-resistant pathogenic fungi.^11,12^ Global epidemics are increasingly being caused by drug-resistant (and multidrug-resistant) fungal pathogens, including *Aspergillus fumigatus*, *Candida glabrata*, *Cryptococcus neoformans*,^5,13−16^ and, more recently, *Candida auris*, a pathogen with the potential for extensive multidrug resistance.^17−20^ Notably, infections with drug-resistant fungi are associated with mortality rates in the range of 50%, granting them high priority for new drug development.^21−24^ An increasingly favored approach to rapidly overcome the shortage in fungal drug targets and drug classes is to enhance the efficacy of existing antifungal drugs through combination therapies.^25^ To date, several FDA-approved drugs have been reported to synergize with antifungal drugs, including inhibitors of Hsp90, calcineurin, TOR, and PKC pathways, and drug efflux inhibitors.^5,26−29^

**Scheme 1:**
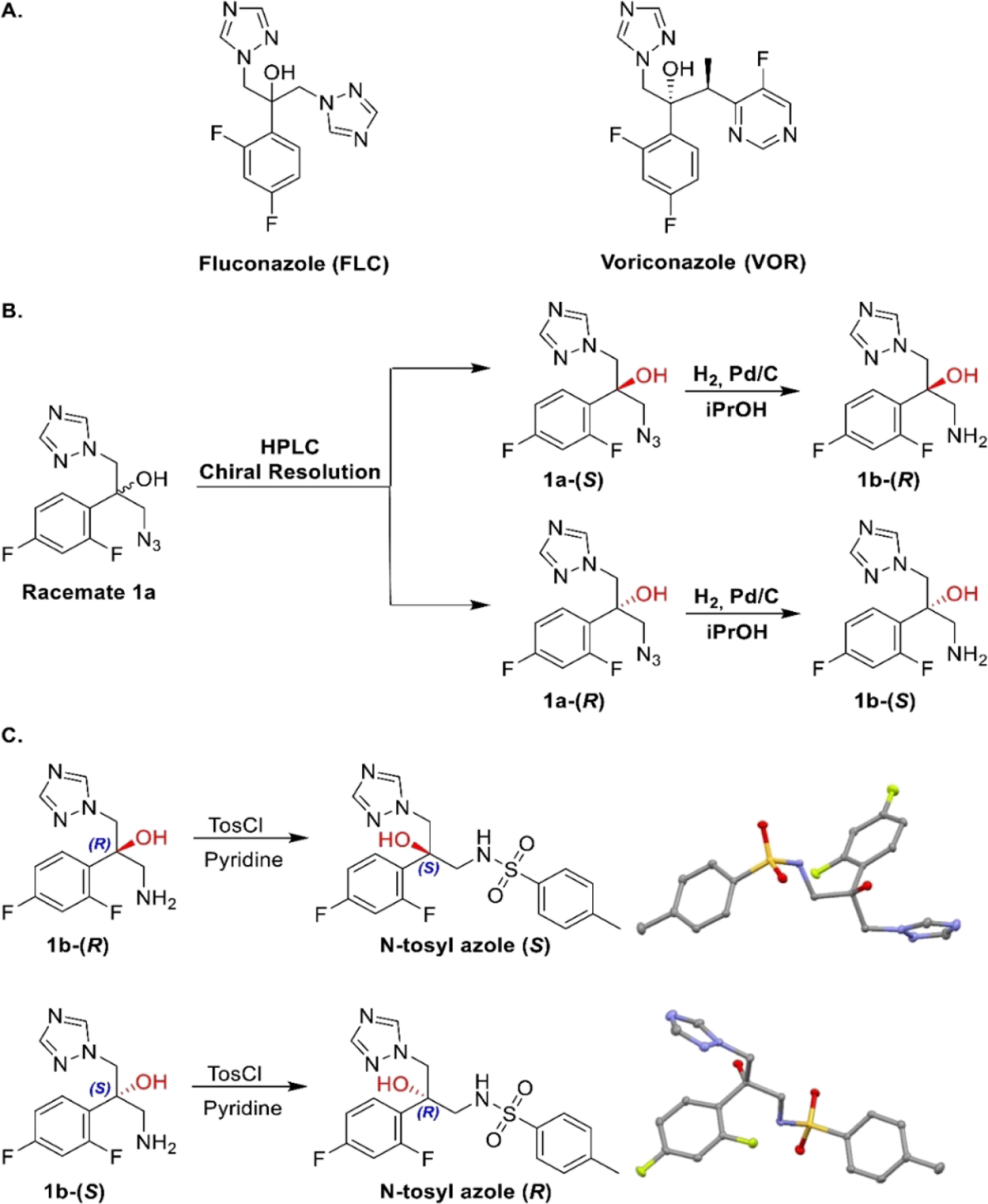
(A) Structure of Clinically Used Antifungal Azole Drugs Fluconazole and Voriconazole; (B) Synthesis of Enantiomerically Pure Antifungal Azole Pharmacophores; (C) Synthesis of Crystallizable *N*-tosyl Derivatives of the Enantiomerically Pure Pharmacophores and X-ray Structures Confirming Their Absolute Configuration

Several clinically used nonsteroidal anti-inflammatory drugs that act by inhibiting cyclooxygenase (COX) enzymes, including ibuprofen, aspirin, and indomethacin, have been shown to possess moderate antifungal activity; the mechanism is unknown.^30−33^ When used in combination with the most commonly used antifungal azole drug fluconazole (**FLC**, Scheme 1A), COX inhibitors significantly improve antifungal efficacy in vitro.^34,35^ The antifungal efficacy of this type of combination was validated in animal models.^36,37^ For example, ibuprofen was shown to effectively synergize with **FLC** against azole-resistant *C. albicans*.^34,38^ A similar effect was observed for a combination of **FLC** and FK506, a 23-membered-ring macrolide immunosuppressant, that also acts as a broad-spectrum inhibitor of pleiotropic drug resistance ATP-binding cassette transporters.^26,39,40^**FLC**-resistant isolates revert to **FLC** susceptible after incubation with ibuprofen yet retain high levels of expression of CDR1 and CDR2 efflux pumps.^41^ It was shown that ibuprofen can alter the expression of the genes encoding the efflux pumps and that it may also act directly as an efflux pump blocker.^42,43^

The arachidonic acid pathway has been associated with the yeast-to-hyphae morphogenesis in several species of *Candida*,^44,45^ the most commonly diagnosed pathogens causing fungal-born infectious diseases in humans.^46,47^ In mammals, nonsteroidal anti-inflammatory drugs such as COX inhibitors reduce the formation of prostaglandins generated via the arachidonic acid pathway.^48^ Prostaglandins are involved in the morphogenesis and pathogenicity of yeast and mediate the host inflammatory response.^32,49^ Prostaglandin E2 (PGE_2_) regulates growth and colonization and promotes the formation of biofilms of several *Candida* species.^50,51^ Several studies have shown that reduced PGE_2_ production limits the virulence of pathogenic fungi, suggesting that the use of inhibitors of the arachidonic acid pathway could improve outcomes of fungal infections.^36,44,45^

Physicians are reluctant to prescribe COX inhibitors to patients with infections due to their anti-inflammatory effects as these agents reduce the ability of the innate immune system to combat the pathogen. The efficacy of combination treatments heavily relies on the pharmacokinetic and pharmacodynamic properties of each of the drugs in the combination.^52^ Moreover, COX-inhibiting drugs are known to induce gastrointestinal irritation. COX-1 is mainly responsible for mucus formation in the gastrointestinal tract and its inhibition is therefore blamed for inducing irritation.^53−56^ These effects have been attributed to the carboxylic acid functionality that is common to all classical COX-inhibiting nonsteroidal anti-inflammatory drugs.^57,58^ Ester and amide derivatives of these drugs maintain COX inhibition but cause less gastrointestinal problems, suggesting that the carboxylic acid group present in these drugs may not be required for COX inhibition.^59,60^ Based on these observations, in this study, we sought to incorporate the antifungal properties of COX inhibitors with those of antifungal azoles by conjugating the amine-functionalized pharmacophore of **FLC** to different COX inhibitors via their carboxylic acid to form hybrid drugs. We report here on the synthesis and in vitro efficacies of dual-acting antifungals composed of the pharmacophore of **FLC** and a collection of clinically used COX inhibitors.

## Results and Discussion

### Synthesis of Diastereoisomers and Enantiomers of Azole-COX Inhibitor Hybrids

To synthesize the hybrids composed of an antifungal azole pharmacophore and a COX inhibitor, we prepared racemic mixture **1a**, the azide-functionalized pharmacophore of the first and second-generation antifungal azole drugs **FLC** and voriconazole (**VOR**) (Scheme 1A) as we previously reported.^61^ Enantiomerically pure **1a-(*S*)** and **1a-(*R*)** were readily obtained by HPLC using a preparative amylose-based chiral resolution column (Scheme 1B). The azide-functionalized pharmacophores **1a-(*S*)** and **1a-(*R*)** were then subjected to catalytic hydrogenation to afford the corresponding amine-functionalized derivatives **1b-(*R*)** and **1b-(*S*)**, respectively (Scheme 1B). The absolute configurations of the two amine-functionalized enantiomers of the azole pharmacophore were assigned by solving the X-ray structures of crystals of the two enantiomerically pure *N*-tosyl derivatives of the amine-functionalized derivatives **1b-(*R*)** and **1b-(*S*)**, which readily crystalized from acetonitrile (Scheme 1C).

**Scheme 2:**
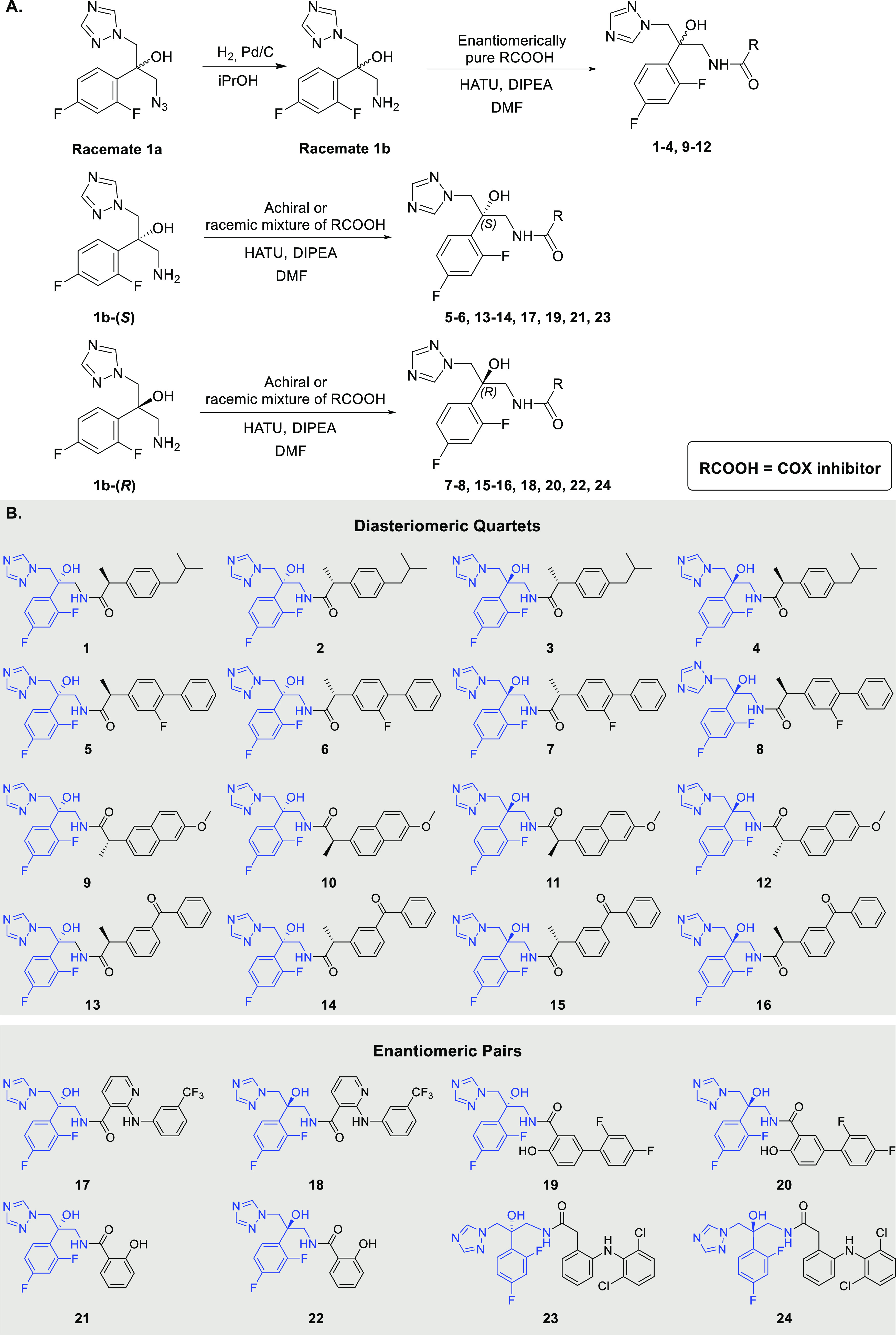
(A) General Synthesis of Hybrids **1**–**24**; (B) Structures of the 24 COX Inhibitor-Azole Hybrids Synthesized

We generated 24 hybrids by forming an amide bond between the primary amine of the azole pharmacophore and the carboxylic acid of the COX inhibitor following the strategies described in Scheme 2A. Four of the COX inhibitors, ibuprofen, flurbiprofen, naproxen, and ketoprofen, contain a chiral center and were used for the generation of all four diastereomers of each hybrid (**1–4**, **5–8**, **9–12**, and **13–16**, respectively, Scheme 2B). The achiral COX inhibitors niflumic acid, diflunisal, salicylic acid and diclofenac were used in the synthesis of enantiomeric azole pairs (**17**–***24***, respectively, Scheme 2B).

Hybrids **1**–**4** and **9**–**12** were prepared by coupling of an enantiomerically pure COX inhibitor to racemate **1b** (Scheme 2A). Hybrids **5**, **6**, **13**, and **14** were prepared by coupling the enantiomerically pure amine-functionalized azole pharmacophore **1b-(*S*)** to a racemate of the COX inhibitors. Hybrids **17**, **19**, **21**, and **23** were prepared by coupling the enantiomerically pure amine-functionalized azole pharmacophore **1b-(*S*)** to achiral COX inhibitors. The same strategy was applied for the preparation of hybrids **7**, **8**, **15**, **16**, **18**, **20**, **22**, and **24** from the enantiomerically pure amine-functionalized azole pharmacophore **1b-(*R*)** (Scheme 2A). The purities of the 24 hybrids were determined by chiral semi-preparative HPLC column and confirmed to be ≥95% (Table S1, Figures S2–S25). The structures of the hybrids synthesized were verified using ^1^H, ^13^C, and ^19^F NMR (Figures S28–S99) and HRMS.

### Antifungal Potencies of the Hybrids and the Effects of Chiral Centers

**Figure 1:**
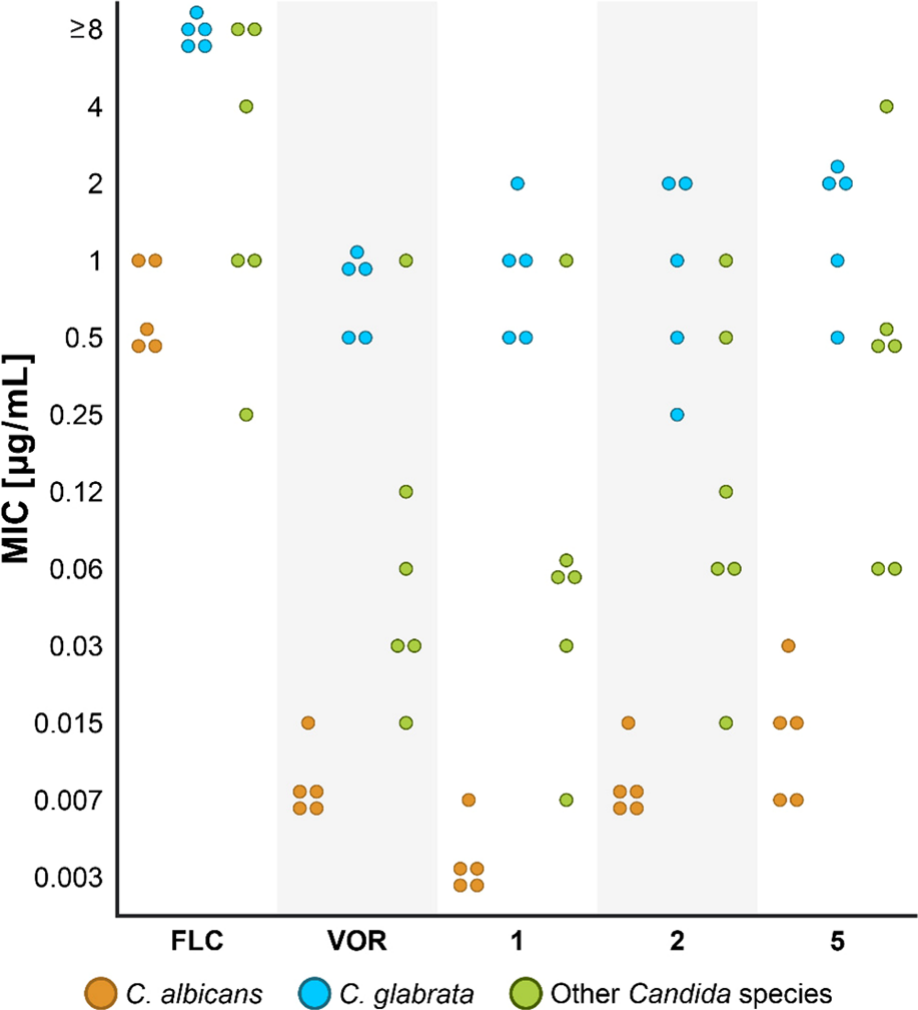
Antifungal activities (MIC_80_ values) of clinically used **FLC** and **VOR** and of the three most potent azole-COX inhibitor hybrids **1**, **2**, and **5**. MIC_80_ values were determined using the broth microdilution method over a concentration range of 0.003–64 μg/mL. Orange circles represent *C. albicans* strains, yellow circles represent *C. glabrata* strains, and green circles represent *C. parapsilosis*, *C. tropicalis*, *C. dubliniensis*, and *C. auris*. Cells were grown in YPAD medium at 30 °C (For *C. auris* strains 37 °C) for 24 h. Each concentration was tested in triplicate, and the results were confirmed in at least two independent experiments.

The antifungal activities of the 24 azole-COX inhibitor hybrids were evaluated against a panel of 16 strains representing seven different species of the genus *Candida*. *Candida* species cause both superficial and systemic infections.^62^ The panel included strains of *C. albicans*, *C. glabrata*, *C. parapsilosis*, *C. tropicalis*, *C. guilliermondii*, *C. dubliniensis*, and *C. auris* (for strains information see Table S2 in the Supporting Information). To evaluate the antifungal activity, we determined minimal inhibitory concentration 80% (MIC_80_) values, which were defined as the lowest drug concentrations with turbidity (measured at OD_600_) less than or equal to that of specific 1:5 dilutions of the growth control. As controls we tested **FLC** and **VOR**. MIC_80_ values of the 24 hybrids and of the control azole drugs against the 16 *Candida* strains tested are summarized in Figure 1 and in Tables S3–S6 in the Supporting Information.

Of the 24 hybrids, three stood out as the most potent agents with the lowest MIC_80_ values against all of the azole-susceptible strains in the panel: ibuprofen-based hybrids **1** and **2** and flurbiprofen-based hybrid **5** (Figure 1). Of these three hybrids, ibuprofen-based azole **1** had the most potent activity against the majority of the azole-susceptible strains in the panel; this hybrid was up to two orders of magnitude more potent than **FLC** and was as potent as **VOR**.

**Figure 2:**
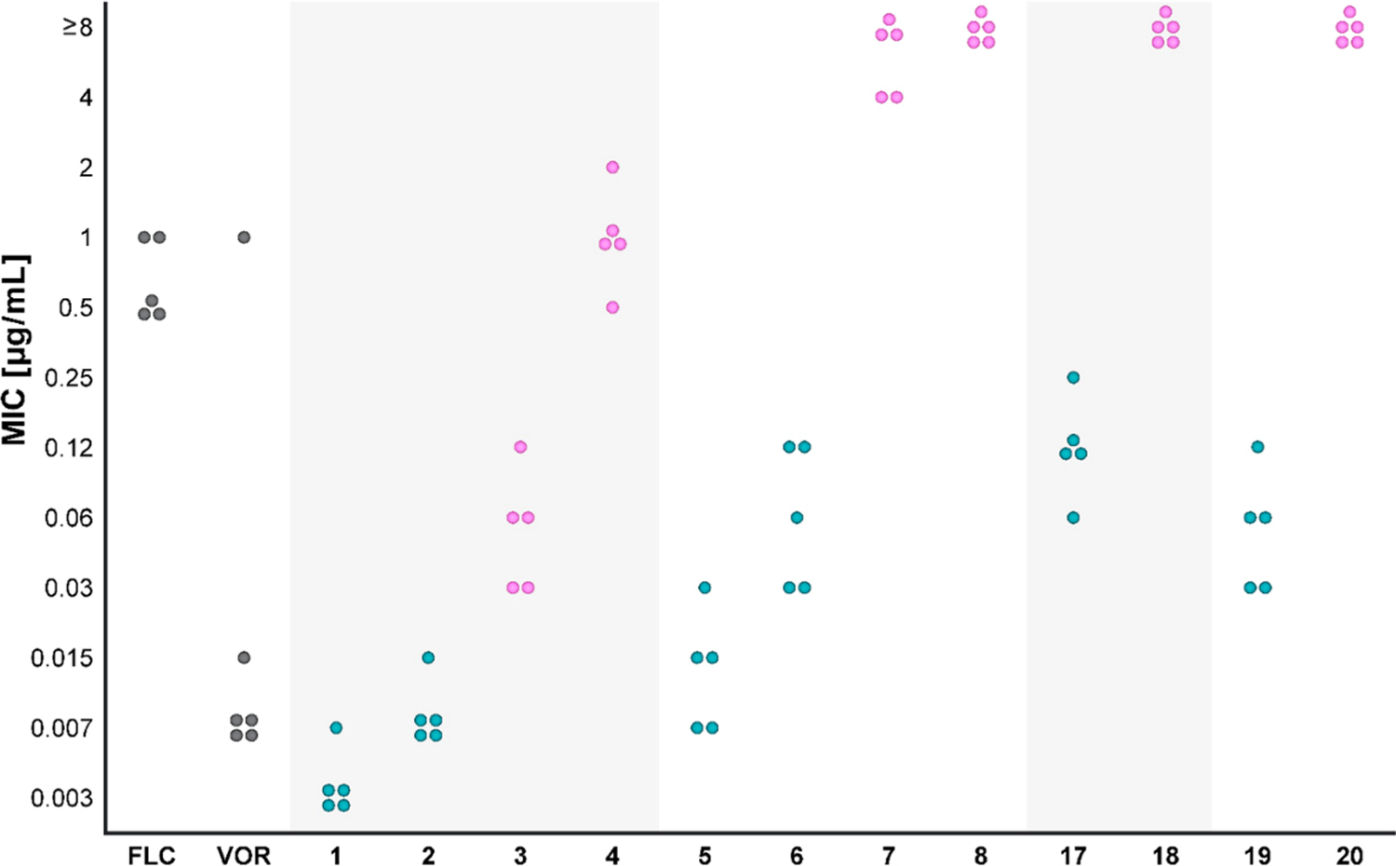
The effect of chirality on antifungal activity against *C. albicans* strains. Black circles represent MIC_80_ values of **FLC** and **VOR**. Blue circles represent MIC_80_ values of hybrids composed of an *S*-configured azole pharmacophore, and pink circles represent MIC_80_ values of hybrids composed of an *R*-configured azole pharmacophore.

In search of structure–activity relationships, we next analyzed the results of the antifungal activity tests in the context of the chiral center or centers of the hybrids. Our analysis revealed a clear connection between the absolute configuration of the chiral center at the benzylic carbon of the azole pharmacophore segment in both the diastereomeric tetrads and enantiomeric pairs. In all cases, the antifungal activity of hybrids with an *S*-configured benzylic carbon of the azole pharmacophore segment had higher potency than the corresponding hybrids with the *R*-configured center. Selected examples of two tetrads (ibuprofen-based **1**–**4** and flurbiprofen-based **5**–**8**) and two enantiomeric pairs (niflumic acid-based **17** and **18** and diflunisal-based **19** and **20**), which demonstrate the superior activity of the *S*- vs *R*-configured benzylic carbon against the azole pharmacophore are presented in Figure 2.

No general correlation could be made between antifungal potency and the chiral center of the COX inhibitor segments of the diastereomeric tetrads; rather, the results depended on the specific COX inhibitor. For example, hybrid **3** composed of *R*-configured ibuprofen was more potent than the corresponding *S*-configured ibuprofen hybrid **4**. In the flurbiprofen tetrad, however, the *S*-configured flurbiprofen hybrid **5** was more potent than the corresponding *R*-configured ibuprofen hybrid **6** (Figure 2). Of note, the chiral center of the azole pharmacophore markedly affected the antifungal activity of the hybrids, and the modest contribution of the chiral center of the COX inhibitor supports the hypothesis that the main target of these dual-acting antifungals is CYP51, the target of the azole class of antifungals. The investigation of the antifungal activity indicated that hybrids prepared by conjugation of the carboxylic acid of COX inhibitors to the amine-functionalized pharmacophore of the azole drug **FLC** can have markedly improved antifungal activity compared to that of **FLC** and comparable to that of the potent second-generation azole **VOR**.

### *Candida* Tolerance to Azole-COX Inhibitor Hybrids Is Lower Than That to FLC and VOR

**Figure 3:**
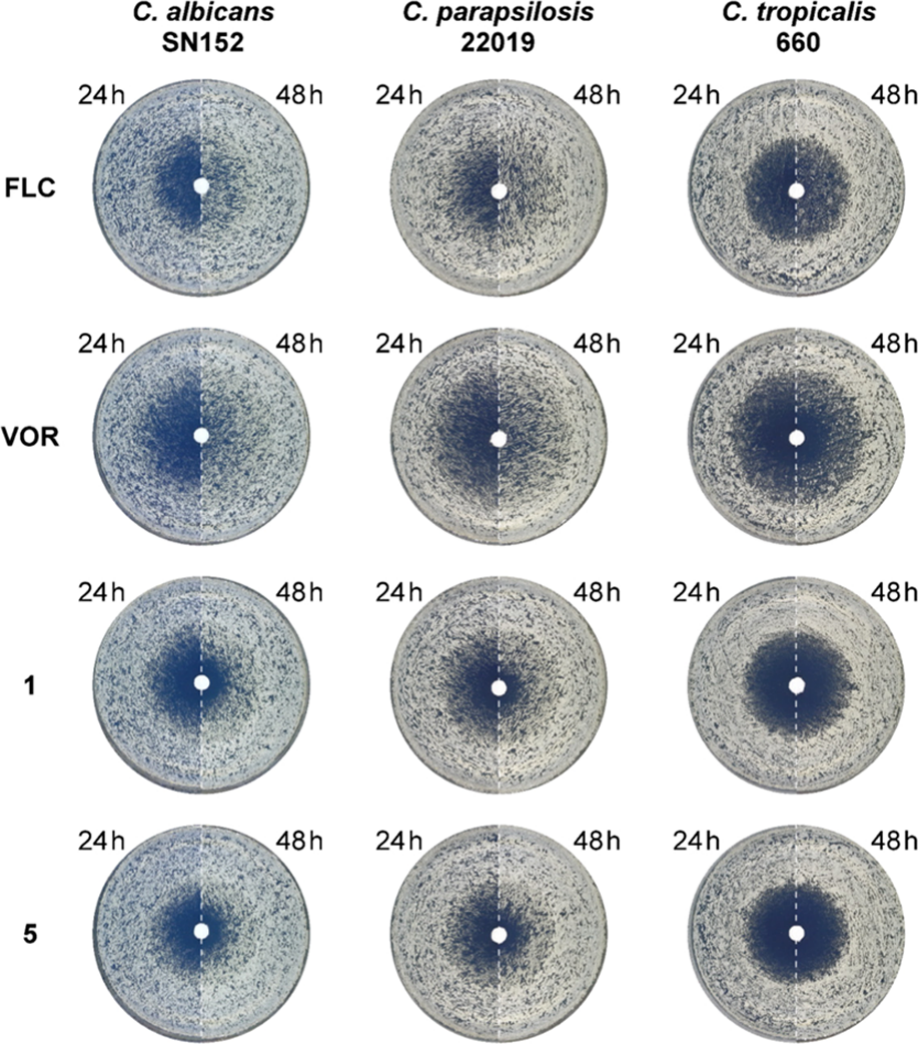
Compared to **FLC** and **VOR**, azole-COX inhibitor hybrids **1** and **5** display reduced tolerance measured by disk diffusion assays. Disk diffusion assays were carried out on casitone agar plates containing disks loaded with 25 μg of the tested hybrids. Plates were imaged after 24 h to evaluate antifungal activity (left half of the plate image) and after 48 h to evaluate tolerance (right half of the plate image).

The majority of treatment failures for patients with invasive candidiasis are caused by apparently susceptible isolates.^63^ For example, during a clinical trial on the treatment of invasive candidiasis, the drug anidulafungin, which belongs to the echinocandin class of antifungal drugs that act by inhibiting cell-wall formation,^64,65^ was significantly superior to **FLC**, although the vast majority of isolates were susceptible to both drugs.^66^ Apparently susceptible isolates resist antifungal drugs by exhibiting tolerance, defined as the ability of a subpopulation of cells to grow slowly at supra-MIC concentrations. Activation of tolerance mechanisms depends on stress response pathways.^67^ Tolerance is, therefore, mechanistically distinct from resistance that relies upon mechanisms that are constantly under alert and do not require activation by stress response signals. Since the subpopulation exhibiting antifungal tolerance is usually characterized by slow growth, it becomes visually detectible after at least 48 h of growth in the presence of the drug, whereas resistance is generally evident after 24 h.^67^ The level of tolerance varies between isolates presumably due to genetic differences, and even within a single genetic isolate, tolerance responses of individual cells may differ significantly.^67^ Tolerance is thus the result of physiological or epigenetic differences rather than genetic variation. Clinical isolates that cause persistent infections and that fail to respond to a single course of **FLC** have higher intrinsic tolerance levels than those isolates that cause nonpersistent infections that are cleared with a single **FLC** course.^67^ This suggests that measurement of tolerance may provide useful prognostic information and there is a need for development of drugs that are unaffected by tolerance. To investigate how tolerance is affected by the azole-COX inhibitor hybrids, we compared hybrids **1** and **5** to **FLC** and **VOR** in a disk diffusion assay. Tolerance was evaluated by comparing the zone of inhibition after 24 h to that after 48 h. The assay was carried out on three representative strains: *C. albicans*, *C. parapsilosis*, and *C. tropicalis* (Figure 3).

After 48 h of incubation with **FLC** or **VOR** disks, the zones of inhibition that had appeared after 24 h of incubation in plates seeded with *C. albicans* SN152 or with *C. parapsilosis* ATCC 22019 were covered by drug tolerant colonies; the drug tolerant subpopulation was smaller for *C. tropicalis* 660. All three tested strains displayed reduced tolerance to both hybrids **1** and **5** compared to the tolerance to **FLC** and **VOR** with the most pronounced effect observed in *C. tropicalis* 660 plates (Figure 3). No correlation could be made between MIC_80_ values and the level of tolerance. For example, the MIC_80_ values of **5**, and **VOR** against *C. parapsilosis* 22019 were 0.5 μg/mL, and 0.015 μg/mL, respectively (Table S6), whereas the observed tolerance of this strain to hybrid **5** was lower than that to **VOR** (Figure 3). Since **VOR** acts predominantly by inhibition CYP51, this suggests that the observed reduced tolerance to the azole-COX inhibitor hybrids is not exclusively due to inhibition of CYP51 and that the antifungal effect of their COX inhibitor segment is likely responsible for the reduction in tolerance to these agents.

### Dual-Acting Azole-COX Inhibitor Hybrids Act Predominantly by Inhibiting Ergosterol Biosynthesis

**Figure 4:**
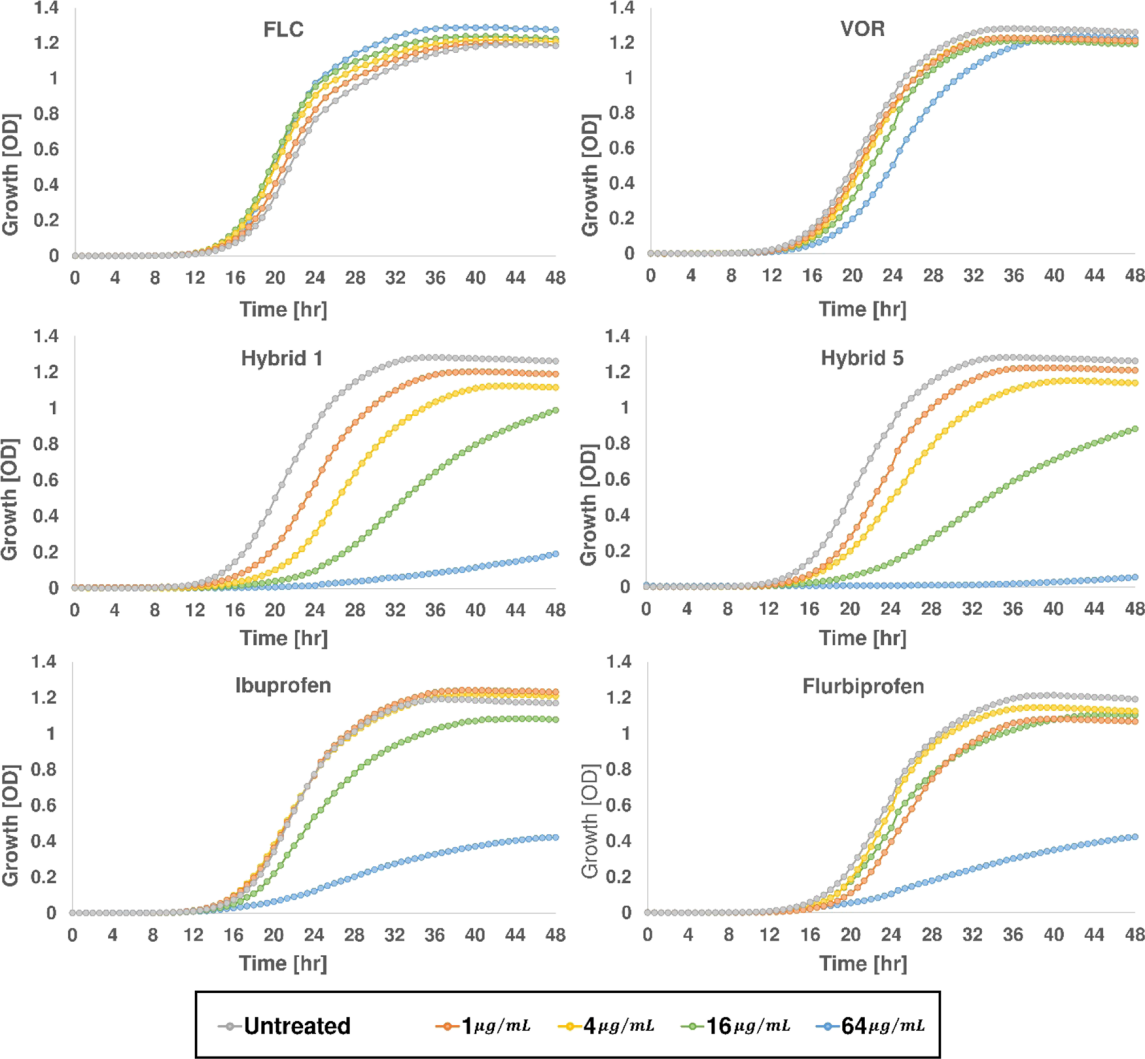
The effect of azole-COX inhibitor hybrids **1** and **5** on the growth of *C. albicans* lacking CYP51, the target of antifungal azoles. Cells of *erg3*ΔΔ*/erg11*ΔΔ mutant *C. albicans* were grown in YPAD media at 30 °C and treated with different concentrations of the tested hybrids. Growth was measured by recording the OD_600_ values every 40 min over a 48 h course on an automated plate reader.

It is well established that clinically used azole antifungals including **FLC** and **VOR** act primarily by preventing ergosterol biosynthesis via inhibition of CYP51.^68,69^ We asked if fungal growth inhibition by the dual-acting hybrids requires the presence of the *ERG11* gene that encodes CYP51.^70^ The antifungal activities of hybrids **1** and **5** and of **FLC** and **VOR** were determined against an *erg3*ΔΔ*/erg11*ΔΔ mutant *C. albicans* strain and against *C. albicans* SN152 from which this double knockout strain was derived (Table S2). The *erg3*ΔΔ*/erg11*ΔΔ mutant is viable despite lacking CYP51, which is essential for aerobic growth unless *ERG3*, which encodes a C-5 sterol desaturase, is inactive.^71^ Yeast growth was followed at OD_600_ over 48 h in 96-well plates containing serial double dilutions of the tested hybrids. The results are summarized in Figure 4.

As expected, when CYP51 is not present, no significant effect on the growth of the double knockout mutant was observed for the entire range of concentrations of **FLC**. Modest reduction in growth was observed in wells treated with **VOR** at 64 μg/mL, the highest concentration tested, presumably due to nonspecific effects of the drug at this high concentration. In contrast, a clear dose-dependent reduction in growth was evident in wells containing hybrids **1** or **5**. Dose-dependent growth reduction was also observed in the presence of free ibuprofen and flurbiprofen, from which hybrids **1** and **5**, respectively, were derived. This supports that the CYP51-independent antifungal effect of the azole-COX inhibitor hybrids **1** and **5** results from their COX inhibitor segments. Of note, the MIC_80_ values of hybrids **1** and **5** against the *erg3*ΔΔ*/erg11*ΔΔ mutant *C. albicans* strain were 64 μg/mL while **FLC** and **VOR** were inactive (Table S4). The MIC_80_ values of these hybrids against *C. albicans* SN152, the parent strain of the *erg3*ΔΔ*/erg11*ΔΔ mutant were 0.003 μg/mL and 0.007 μg/mL, respectively (Table S4). The high MIC_80_ values against the *erg3*ΔΔ*/erg11*ΔΔ mutant relative to those against the parent strain support our hypothesis that the contribution to the antifungal activity of the COX-inhibiting segment in these dual-acting agents is modest compared to that of the inhibition of CYP51.

## Conclusions

It was previously established that nonsteroidal COX-inhibiting anti-inflammatory drugs and azole antifungals synergize to improve antifungal potency. Combination therapies can be affected by differences in pharmacological properties and by side effects of the drugs in the combination. With the goal of overcoming such potential limitations for combinations of azole antifungals and COX inhibitors, we synthesized a novel type of antifungals by linking an azole pharmacophore with a COX inhibitor to form a hybrid drug molecule. These hybrids were prepared by conjugation of a chiral azole pharmacophore to a collection of chiral and achiral COX inhibitors to form 24 chiral hybrids.

The antifungal activity profiles of the hybrids were tested against a diverse panel of *Candida* representing seven of the most encountered species of this common fungal pathogen and compared to the activities of the clinically used azole drugs **FLC** and **VOR**. The antifungal activities of several hybrids were superior to that of **FLC**. Two hybrids, ibuprofen-based **1** and flurbiprofen-based **5**, stood out due to potency significantly higher than **FLC** and comparable to **VOR**. Structure–activity relationship analysis revealed that all hybrids with an *S*-configured azole pharmacophore were more potent antifungals than the corresponding hybrids with an *R*-configured azole pharmacophore. No such generalization could be made for the chiral COX inhibitors. In all hybrids with a chiral COX inhibitor, the contribution of the chiral center of the azole pharmacophore to the antifungal activity of the hybrids was markedly higher compared to that of the chiral center of the COX inhibitor.

Importantly, analysis of tolerance, defined as the ability of a subpopulation of cells to grow in the presence of the drug, revealed that yeast cultures were less likely to be tolerant in the presence of the hybrids **1** and **5** than in the presence of **FLC** and **VOR**. Clinical isolates with high tolerance are associated with persistent infections, suggesting that lower levels of tolerance to a drug may reduce the chances of the persistence and/or reoccurrence of the infection.

Mechanistic investigation revealed that unlike the clinically used **FLC** and **VOR** that target CYP51 as their main mode of action, hybrids **1** and **5** retained activity against an *erg3*ΔΔ*/erg11*ΔΔ mutant *C. albicans* strain, which lacks CYP51. This activity was significantly lower, however, than the activity of these hybrids against the parent *C. albicans* strain from which the mutant lacking the target was derived. This indicates that the antifungal activity of these dual-acting hybrids results mainly from the inhibition of CYP51 yet, unlike **FLC** and **VOR**, the hybrids also act via a second mode of action contributed by the COX-inhibiting segment.

This study offers guidelines for development of potent antifungal agents that incorporate the antifungal activities of azole antifungals and COX inhibitors in hybrid molecules. These new antifungals display potent antifungal activity and, importantly, reduced levels of tolerance. The dual-acting hybrids described here offer attractive leads for further clinical development.

## Experimental Section

### Chemistry

#### General Methods and Instrumentation

^1^H-NMR spectra (including one-dimensional total correlation spectroscopy (1D-TOCSY)) were recorded on BrukerAvance 400 or 500 MHz spectrometers, and chemical shifts (reported in ppm) were calibrated to CD_3_OD (δ = 3.31). ^13^C-NMR spectra were recorded on BrukerAvance 400 or 500 MHz spectrometers at 100 or 125 MHz, respectively. ^19^F-NMR spectra were recorded on BrukerAvance 400 or 500 MHz spectrometers at 375 or 470 MHz, respectively. Multiplicities are reported using the following abbreviations: s = singlet, d = doublet, t = triplet, q = quartet, dd = doublet of doublets, m = multiplet. Coupling constants (*J*) are given in Hz. High-resolution electrospray ionization (HRESI) mass spectra were measured on a Waters Synapt instrument. Chemical reactions were monitored by thin-layer chromatography (TLC) (Merck, Silica gel 60 F_254_). Visualization was achieved using a cerium molybdate stain (5 g (NH_4_)_2_Ce(NO_3_)_6_, 120 g (NH_4_)_6_Mo_7_O_24_·4H_2_O, 80 mL H_2_SO_4_, 720 mL H_2_O) or with UV lamp. All chemicals, unless otherwise stated, were obtained from commercial sources. Reaction products were purified using Geduran Si 60 chromatography (Merck). The preparative reverse-phase high-pressure liquid chromatography (RP-HPLC) system used was an ECOM system equipped with a 5-μm, C-18 Phenomenex Luna Axia column (250 mm × 21.2 mm). The mobile phase was acetonitrile in H_2_O, and the gradient was from 10 to 90% acetonitrile. The flow rate was 20 mL/min. Chiral semi-preparative high-pressure liquid chromatography (HPLC) used was performed on an ECOM system equipped with a 5-μm i-Amylose-3 Phenomenex Lux column (250 mm × 10 mm). The flow rate was 5 mL/min.

### Crystallographic Data

Deposition Numbers 2116277 and 2166299 contain the supplementary crystallographic data for this paper. These data are provided free of charge by the joint Cambridge Crystallographic Data Centre.

#### Azole-Ibuprofen Hybrids (**1** and **4**)

*S*-Ibuprofen (95 mg, 0.46 mmol) was dissolved in dry DMF (2 mL) under argon at 0 °C and then treated with HATU (280 mg, 0.74 mmol) and DIPEA (0.27 mL, 1.55 mmol) and stirred for 10 min at 0 °C. To the reaction mixture, racemate **1b** (103 mg, 0.41 mmol) was added, and the solution was stirred at room temperature. The reaction was monitored by TLC (MeOH/DCM, 1:9). Upon completion after 3 h, the product was extracted with ethyl acetate, washed with H_2_O, dried over MgSO_4_, and concentrated to give the crude diastereomers. The concentrated crude was purified by column chromatography on SiO_2_ using a gradient of MeOH/DCM as eluent to afford the diastereomer mix. The diastereomers were separated by preparative RP-HPLC to afford hybrids **1** and **4**.

#### Azole-Ibuprofen Hybrid (**1**) (65 mg, 73%)

HRESI-MS *m/z* calculated for C_24_H_28_F_2_N_4_O_2_Na, 465.2078; found for [M + Na]^+^, 465.2074. ^1^H NMR (500 MHz, CD_3_OD) δ 8.29 (s, H-2, 1H), 7.76 (s, H-1, 1H), 7.37–7.32 (m, H-3, 1H), 7.01 (s, H-12, H-13, 4H), 6.87–6.83 (m, H-5, 1H), 6.74–6.71 (m, H-4, 1H), 4.65 (d, *J* = 14.3 Hz, H-6, 1H), 4.53 (d, *J* = 14.3 Hz, H-6, 1H), 3.88 (d, *J* = 14.3 Hz, H-7, 1H), 3.55–3.43 (m, H-7, H-10, 2H), 2.42 (d, *J* = 7.2 Hz, H-14, 2H), 1.87–1.75 (m, H-15, 1H), 1.28 (d, *J* = 7.1 Hz, H-11, 3H), 0.88 (d, *J* = 7.4 Hz, H-16, 6H). ^13^C NMR (125 MHz, CD_3_OD) δ 177.4, 162.8 (dd, ^1^*J*_C*-*F_ = 246.2 Hz, ^3^*J*_C*-*F_ = 12.2 Hz), 159.3 (dd, ^1^*J*_C*-*F_ = 245.4 Hz, ^3^*J*_C*-*F_ = 12.0 Hz), 149.9, 144.7, 140.10, 138.3, 130.0, 128.8, 126.6, 123.7, 110.5, 103.5, 75.3, 55.6, 46.3, 45.3, 44.6, 30.0, 21.3, 17.3. ^19^F NMR (470 MHz, CD_3_OD) δ −109.20 (m, F_para_), −113.12 (m, F_ortho_).

#### Azole-Ibuprofen Hybrid (**4**) (49 mg, 55%)

HRESI-MS *m/z* calculated for C_24_H_28_F_2_N_4_O_2_Na, 465.2078; found for [M + Na]^+^, 465.2067. ^1^H NMR (500 MHz, CD_3_OD) δ 8.28 (s, H-2, 1H), 7.76 (s, H-1, 1H), 7.32–7.27 (m, H-3, 1H), 7.00 (s, H-12, H-13, 4H), 6.85–6.80 (m, H-5, 1H), 6.71–6.66 (m, H-4, 1H), 4.57 (d, *J* = 14.3 Hz, H-6, 1H), 4.45 (d, *J* = 14.3 Hz, H-6, 1H), 3.72 (d, *J* = 14.3 Hz, H-7, 1H), 3.64 (d, *J* = 14.3 Hz, H-7, 1H), 3.50 (q, *J* = 7.0 Hz, H-10, 1H), 2.42 (d, *J* = 7.2 Hz, H-14, 2H), 1.86–1.74 (m, H-15, 1H), 1.28 (d, *J* = 7.1 Hz, H-11, 3H), 0.86 (d, *J* = 6.6 Hz, H-16, 6H). ^13^C NMR (125 MHz, CD_3_OD) δ 177.8, 162.8 (dd, ^1^*J*_C*-*F_ = 247.5 Hz, ^3^*J*_C*-*F_ = 12.2 Hz), 159.2 (dd, ^1^*J*_C*-*F_ = 246.6 Hz, ^3^*J*_C*-*F_ = 12.1 Hz), 149.9, 144.8, 140.1, 138.5, 130.0, 128.8, 126.6, 123.8, 110.6, 103.4, 75.6, 55.6, 46.7, 45.2, 44.5, 30.0, 21.3, 17.0. ^19^F NMR (470 MHz, CD_3_OD) δ −109.62 (m, F_para_), −113.16 (m, F_ortho_).

Azole-COX inhibitor hybrids **2**, **3**, **5–18** were prepared in the same manner as hybrids **1** and **4** with the following modifications:

#### Azole-Ibuprofen Hybrids (**2**, **3**)

*R*-Ibuprofen (99 mg, 0.48 mmol), HATU (299 mg, 0.79 mmol), DIPEA (0.27 mL, 1.55 mmol), and racemate **1b** (105 mg, 0.41 mmol).

#### Azole-Ibuprofen Hybrid **2** (60 mg, 66%)

HRESI-MS *m/z* calculated for C_24_H_29_F_2_N_4_O_2_, 443.2259; found for [M + H]^+^, 443.2258. ^1^H NMR (500 MHz, CD_3_OD) δ 8.28 (s, H-2, 1H), 7.76 (s, H-1, 1H), 7.32–7.27 (m, H-3, 1H), 7.00 (s, H-12, H-13, 4H), 6.85–6.80 (m, H-5, 1H), 6.71–6.67 (m, H-4, 1H), 4.57 (d, *J* = 14.3 Hz, H-6, 1H), 4.45 (d, *J* = 14.3 Hz, H-6, 1H), 3.72 (d, *J* = 14.3, H-7, 1H), 3.64 (d, *J* = 14.3, H-7, 1H), 3.50 (q, *J* = 7.1 Hz, H-10, 1H), 2.42 (d, *J* = 7.2 Hz, H-14, 2H), 1.86–1.74 (m, H-15, 1H), 1.28 (d, *J* = 7.1 Hz, H-11, 3H), 0.86 (d, *J* = 6.6, H-16, 6H). ^13^C NMR (125 MHz, CD_3_OD) δ 177.8, 162.82 (dd, ^1^*J*_C*-*F_ = 247.5 Hz, ^3^*J*_C*-*F_ = 12.2 Hz), 159.16 (dd, ^1^*J*_C*-*F_ = 246.5 Hz, ^3^*J*_C*-*F_ = 12.0 Hz), 149.9, 144.8, 140.1, 138.5, 130.0, 128.9, 126.6, 123.8, 110.6, 103.4, 75.6, 55.6, 46.7, 45.2, 44.6, 30.0, 21.3, 17.0. ^19^F NMR (470 MHz, CD_3_OD) δ −109.65 (m, F_para_), −113.19 (m, F_ortho_).

#### Azole-Ibuprofen Hybrid 3 (78 mg, 85%)

HRESI-MS *m/z* calculated for C_24_H_28_F_2_N_4_O_2_Na, 465.2078; found for [M + Na]^+^, 465.2083. ^1^H NMR (500 MHz, CD_3_OD) δ 8.29 (s, H-2, 1H), 7.77 (s, H-1, 1H), 7.37–7.32 (m, H-3, 1H), 7.02 (s, H-12, H-13, 4H), 6.88–6.83 (m, H-5, 1H), 6.75–6.69 (m, H-4, 1H), 4.65 (d, *J* = 14.3 Hz, H-6, 1H), 4.53 (d, *J* = 14.3 Hz, H-6, 1H), 3.89 (d, *J* = 14.3, H-7, 1H), 3.52–3.47 (m, H-7, H-10, 2H), 2.42 (d, *J* = 7.2 Hz, H-14, 2H), 1.85–1.77 (m, H-15, 1H), 1.29 (d, *J* = 7.1 Hz, H-11, 3H), 0.88 (d, *J* = 6.6, H-16, 6H). ^13^C NMR (125 MHz, CD_3_OD) δ 177.4, 162.8 (dd, ^1^*J*_C*-*F_ = 246.1, ^3^*J*_C*-*F_ = 12.2 Hz), 159.3 (dd, ^1^*J*_C*-*F_ = 245.4, ^3^*J*_C*-*F_ = 12.0 Hz), 149.9, 144.7, 140.1, 138.3, 130.0, 128.8, 126.6, 123.7, 110.5, 103.5, 75.3, 55.6, 46.3, 45.4, 44.6, 30.0, 21.3, 17.3. ^19^F NMR (470 MHz, CD_3_OD) δ −109.19 (m, F_para_), −113.14 (m, F_ortho_).

#### Azole-Flurbiprofen Hybrids (**5**, **6**)

Flurbiprofen (127 mg, 0.52 mmol), HATU (311 mg, 0.82 mmol), DIPEA (0.27 mL, 1.55 mmol), and **1b-(*S*)** (100 mg, 0.39 mmol).

#### Azole-Flurbiprofen Hybrid **5** (81 mg, 86%)

HRESI-MS *m/z* calculated for C_26_H_23_F_3_N_4_O_2_Na, 503.1671; found for [M + Na]^+^, 503.1670. ^1^H NMR (500 MHz, CD_3_OD) δ 8.32 (s, H-2, 1H), 7.79 (s, H-1, 1H), 7.52–7.50 (m, H-15, 2H), 7.44–7.41 (m, H-16, 2H), 7.37–7.30 (m, H-3, H-13, H-17, 3H), 7.00 (dd, *J* = 8.0 Hz, 1.7 Hz, H-12, 1H), 6.96 (dd, *J* = 11.9 Hz, 1.6 Hz, H-14, 1H), 6.88–6.83 (m, H-5, 1H), 6.70–6.65 (m, H-4, 1H), 4.67 (d, *J* = 14.4 Hz, H-6, 1H), 4.59 (d, *J* = 14.4 Hz, H-6, 1H), 4.02 (d, *J* = 14.8 Hz, H-7, 1H), 3.58 (q, *J* = 7.1 Hz H-7, 1H), 3.46 (d, *J* = 14.1 Hz, H-7, 1H), 1.33 (d, *J* = 7.1 Hz, H-11, 3H). ^13^C NMR (100 MHz, CD_3_OD) δ 176.1, 162.8 (dd, ^1^*J*_C*-*F_ = 248.4 Hz, ^3^*J*_C*-*F_ = 12.5 Hz), 159.4 (d, ^1^*J*_C*-*F_ = 245.3 Hz), 159.3 (dd, ^1^*J*_C*-*F_ = 247.3 Hz, ^3^*J*_C*-*F_ = 11.4 Hz), 150.0, 144.7, 142.8, 135.5, 130.3, 130.0, 128.5, 128.1, 127.4, 127.3, 123.5, 123.2, 114.4, 110.3, 103.4, 75.2, 55.6, 46.1, 45.1, 17.3. ^19^F NMR (470 MHz, CD_3_OD) δ −109.03 (m, F_para_), −112.82 (m, F_ortho_), −119.72 (m, F_meta_).

#### Azole-Flurbiprofen Hybrid **6** (63 mg, 67%)

HRESI-MS *m/z* calculated for C_26_H_23_F_3_N_4_O_2_Na, 503.1671; found for [M + Na]^+^, 503.1668. ^1^H NMR (500 MHz, CD_3_OD) δ 8.34 (s, H-2, 1H), 7.80 (s, H-1, 1H), 7.53–7.51 (m, H-15, 2H), 7.46–7.42 (m, H-16, 2H), 7.38–7.30 (m, H-3, H-13, H-17, 3H), 7.00 (dd, *J* = 8.0 Hz, 1.8 Hz, H-12, 1H), 6.95 (dd, *J* = 11.8 Hz, 1.6 Hz, H-14, 1H), 6.88–6.83 (m, H-5, 1H), 6.70–6.66 (m, H-4, 1H), 4.60 (d, *J* = 14.2 Hz, H-6, 1H), 4.54 (d, *J* = 14.2 Hz, H-6, 1H), 3.80 (d, *J* = 14.2 Hz, H-7, 1H), 3.65–3.55 (m, H-7, H-10, 2H), 1.33 (d, *J* = 7.0 Hz, H-11, 3H). ^13^C NMR (125 MHz, CD_3_OD) δ 178.5, 164.4 (dd, ^1^*J*_C*-*F_ = 247.9 Hz, ^3^*J*_C*-*F_ = 12.4 Hz), 161.0 (d, ^1^*J*_C*-*F_ = 246.9 Hz), 160.7 (dd, ^1^*J*_C*-*F_ = 246.9 Hz, ^3^*J*_C*-*F_ = 12.4 Hz), 151.5, 146.4, 144.5, 137.0, 131.9, 131.6, 130.1, 129.7, 129.1, 128.9, 125.3, 124.7, 116.0, 112.1, 104.9, 77.2, 57.3, 48.4, 46.5, 18.5. ^19^F NMR (470 MHz, CD_3_OD) δ −109.69 (m, F_para_), −112.86 (m, F_ortho_), −119.73 (m, F_meta_).

#### Azole-Flurbiprofen Hybrid (**7**, **8**)

Flurbiprofen (149 mg, 0.61 mmol), HATU (387 mg, 1.02 mmol), DIPEA (0.40 mL, 2.29 mmol), and **1b-(*R*)** (130 mg, 0.51 mmol).

#### Azole-Flurbiprofen Hybrid **7** (110 mg, 90%)

HRESI-MS *m/z* calculated for C_26_H_23_F_3_N_4_O_2_Na, 503.1671; found for [M + Na]^+^, 503.1670. ^1^H NMR (500 MHz, CD_3_OD) δ 8.32 (s, H-2, 1H), 7.80 (s, H-1, 1H), 7.53–7.51 (m, H-15, 2H), 7.45–7.42 (m, H-16, 2H), 7.38–7.31 (m, H-3, H-13, H-17, 3H), 7.00 (dd, *J* = 8.0 Hz, 1.6 Hz, H-12, 1H), 6.96 (dd, *J* = 11.9 Hz, 1.6 Hz, H-14, 1H), 6.88–6.83 (m, H-5, 1H), 6.70–6.66 (m, H-4, 1H), 4.69 (d, *J* = 14.2 Hz, H-6, 1H), 4.60 (d, *J* = 14.2 Hz, H-6, 1H), 4.02 (d, *J* = 14.2 Hz, H-7, 1H), 3.58 (q, *J* = 7.1 Hz, H-7, 1H), 3.47 (d, *J* = 14.2 Hz, H-7, 1H), 1.33 (d, *J* = 7.1 Hz, H-11, 3H). ^13^C NMR (125 MHz, CD_3_OD) δ 177.6, 164.3 (dd, ^1^*J*_C*-*F_ = 247.9 Hz, ^3^*J*_C*-*F_ = 12.4 Hz), 160.9 (d, ^1^*J*_C*-*F_ = 246.9 Hz), 160.8 (dd, ^1^*J*_C*-*F_ = 246.9 Hz, ^3^*J*_C*-*F_ = 11.4 Hz), 151.5, 146.2, 144.3, 137.0, 131.7, 131.5, 130.0, 129.6, 128.9, 128.8, 125.1, 124.7, 115.9, 111.8, 104.9, 76.7, 57.1, 47.6, 46.6, 18.8. ^19^F NMR (470 MHz, CD_3_OD) δ −109.03 (m, F_para_), −112.86 (m, F_ortho_), −119.75 (m, F_meta_).

#### Azole-Flurbiprofen Hybrid **8** (107 mg, 87%)

HRESI-MS *m/z* calculated for C_26_H_23_F_3_N_4_O_2_Na, 503.1671; found for [M + Na]^+^, 503.16680. ^1^H NMR (400 MHz, CD_3_OD) δ 8.34 (s, H-2, 1H), 7.80 (s, H-1, 1H), 7.54–7.51 (m, H-15, 2H), 7.46–7.42 (m, H-16, 2H), 7.39–7.30 (m, H-3, H-13, H-17, 3H), 7.00 (dd, *J* = 7.9 Hz, 1.7 Hz, H-12, 1H), 6.95 (dd, *J* = 11.9 Hz, 1.7 Hz, H-14, 1H), 6.89–6.83 (m, H-5, 1H), 6.71–6.66 (m, H-4, 1H), 4.61 (d, *J* = 14.3 Hz, H-6, 1H), 4.54 (d, *J* = 14.3 Hz, H-6, 1H), 3.81 (d, *J* = 14.1 Hz, H-7, 1H), 3.65–3.55 (m, H-7, H-10, 2H), 1.33 (d, *J* = 7.1 Hz, H-11, 3H). ^13^C NMR (100 MHz, CD_3_OD) δ 178.5, 165.9 (dd, ^1^*J*_C*-*F_ = 247.3 Hz, ^3^*J*_C*-*F_ = 11.9 Hz), 161.0 (d, ^1^*J*_C*-*F_ = 246.2 Hz), 160.7 (dd, ^1^*J*_C*-*F_ = 247.3 Hz, ^3^*J*_C*-*F_ = 11.9 Hz), 151.5, 146.4, 144.4, 137.0, 131.9, 131.5, 130.1, 129.7, 129.1, 128.9, 125.3, 124.7, 116.0, 112.0, 104.9, 77.2, 57.3, 47.6, 46.5, 18.5. ^19^F NMR (375 MHz, CD_3_OD) δ −109.87 (m, F_para_), −113.01 (m, F_ortho_), −119.87 (m, F_meta_).

#### Azole-Naproxen Hybrids (**9**, **12**)

*S*-Naproxen (118 mg, 0.51 mmol), HATU (330 mg, 0.87 mmol), DIPEA (0.30 mL, 1.72 mmol), and **1b** (106 mg, 0.42 mmol).

#### Azole-Naproxen Hybrid **9** (60 mg, 62%)

HRESI-MS *m/z* calculated for C_25_H_25_F_2_N_4_O_3_, 467.1895; found for [M + H]^+^, 467.1894. ^1^H NMR (500 MHz, CD_3_OD) δ 8.28 (s, H-2, 1H), 7.78 (s, H-1, 1H), 7.68 (d, *J* = 9.0 Hz, H-14, 1H), 7.65 (d, *J* = 8.6 Hz, H-15, 1H), 7.56 (s, H-12, 1H), 7.30–7.24 (m, H-3, 1H), 7.22–7.19 (m, H-13, H-17, 2H), 7.13 (dd, *J* = 9.2 Hz, 2.5 Hz, H-16, 1H), 6.83–6.78 (m, H-5, 1H), 6.53–6.49 (m, H-4, 1H), 4.66 (d, *J* = 14.3 Hz, H-6, 1H), 4.55 (d, *J* = 14.4 Hz, H-6, 1H), 3.95–3.91 (m, H-7, H-18, 4H), 3.37 (q, *J* = 7.1 Hz, H-10, 1H), 3.51 (d, *J* = 14.1 Hz, H-7, 1H), 1.40 (d, *J* = 7.1 Hz, H-11, 3H). ^13^C NMR (125 MHz, CD_3_OD) δ 178.6, 163.2 (dd, ^1^*J*_C*-*F_ = 245.0 Hz, ^3^*J*_C*-*F_ = 11.1 Hz), 160.5 (dd, ^1^*J*_C*-*F_ = 245.9 Hz, ^3^*J*_C*-*F_ = 13.2 Hz), 159.2, 151.4, 146.2, 137.6, 135.3, 131.4, 130.4, 130.3, 128.2, 127.0, 126.8, 125.0, 120.0, 111.8, 106.7, 104.9, 76.7, 57.1, 55.8, 47.7, 47.2, 18.7. ^19^F NMR (470 MHz, CD_3_OD) δ −109.29 (m, F_para_), −112.99 (m, F_ortho_).

#### Azole-Naproxen Hybrid **12** (42 mg, 43%)

HRESI-MS *m/z* calculated for C_25_H_25_F_2_N_4_O_3_, 467.1895; found for [M + H]^+^, 467.1892. ^1^H NMR (500 MHz, CD_3_OD) δ 8.27 (s, H-2, 1H), 7.78 (s, H-1, 1H), 7.68 (d, *J* = 9.1 Hz, H-14, 1H), 7.64 (d, *J* = 8.5 Hz, H-15, 1H), 7.54 (s, H-12, 1H), 7.22–7.12 (m, H-3, H-13, H-16, H-17, 4H), 6.80–6.74 (m, H-5, 1H), 6.44–6.39 (m, H-4, 1H), 4.57 (d, *J* = 14.2 Hz, H-6, 1H), 4.49 (d, *J* = 14.3 Hz, H-6, 1H), 3.93 (s, H-18, 3H), 3.75 (d, *J* = 14.3 Hz, H-7, 1H), 3.70–3.65 (m, H-7, H-10, 2H), 1.41 (d, *J* = 7.1 Hz, H-11, 3H). ^13^C NMR (125 MHz, CD_3_OD) δ 179.3, 164.2 (dd, ^1^*J*_C*-*F_ = 246.8 Hz, ^3^*J*_C*-*F_ = 11.4 Hz), 160.5 (dd, ^1^*J*_C*-*F_ = 246.8 Hz, ^3^*J*_C*-*F_ = 12.6 Hz), 159.3, 151.4, 146.3, 137.7, 135.3, 131.4, 130.4, 130.3, 128.3, 127.0, 126.8, 125.1, 120.0, 111.9, 106.7, 104.8, 77.2, 57.1, 55.8, 48.1, 47.0, 18.3. ^19^F NMR (470 MHz, CD_3_OD) δ −109.48 (m, F_para_), −113.05 (m, F_ortho_).

#### Azole-Naproxen Hybrids (**10**, **11**)

*R*-Naproxen (138 mg, 0.60 mmol), HATU (375 mg, 0.99 mmol), DIPEA (0.34 mL, 1.95 mmol), and racemate **1b** (122 mg, 0.48 mmol).

#### Azole-Naproxen Hybrid **10** (41 mg, 35%)

HRESI-MS *m/z* calculated for C_25_H_25_F_2_N_4_O_3_, 467.1895; found for [M + H]^+^, 467.1896. ^1^H NMR (500 MHz, CD_3_OD) δ 8.25 (s, H-2, 1H), 7.77 (s, H-1, 1H), 7.65 (d, *J* = 9.0 Hz, H-14, 1H), 7.62 (d, *J* = 8.5 Hz, H-15, 1H), 7.52 (s, H-12, 1H), 7.20–7.11 (m, H-3, H-13, H-16, H-17, 4H), 6.78–6.73 (m, H-5, 1H), 6.42–6.37 (m, H-4, 1H), 4.55 (d, *J* = 14.5 Hz, H-6, 1H), 4.47 (d, *J* = 14.5 Hz, H-6, 1H), 3.91 (s, H-18, 3H), 3.73 (d, *J* = 14.5 Hz, H-7, 1H), 3.68–3.63 (m, H-7, H-10, 2H), 1.39 (d, *J* = 7.1 Hz, H-11, 3H). ^13^C NMR (125 MHz, CD_3_OD) δ 179.2, 164.2 (dd, ^1^*J*_C*-*F_ = 247.3 Hz, ^3^*J*_C*-*F_ = 12.3 Hz), 160.4 (dd, ^1^*J*_C*-*F_ = 246.8 Hz, ^3^*J*_C*-*F_ = 12.3 Hz), 159.2, 151.4, 146.2, 137.7, 135.3, 131.4, 130.4, 130.3, 128.2, 127.0, 126.8, 125.1, 120.0, 111.9, 106.7, 104.7, 77.1, 57.1, 55.8, 48.1, 47.0, 18.3. ^19^F NMR (470 MHz, CD_3_OD) δ −109.81 (m, F_para_), −113.01 (m, F_ortho_).

#### Azole-Naproxen Hybrid **11** (59 mg, 50%)

HRESI-MS *m/z* calculated for C_25_H_25_F_2_N_4_O_3_, 467.1895; found for [M + H]^+^, 467.1893. ^1^H NMR (500 MHz, CD_3_OD) δ 8.27 (s, H-2, 1H), 7.77 (s, H-1, 1H), 7.67 (d, *J* = 8.9 Hz, H-14, 1H), 7.63 (d, *J* = 8.6 Hz, H-15, 1H), 7.54 (s, H-12, 1H), 7.28–7.23 (m, H-3, 1H), 7.20–7.18 (m, H-13, H-17, 2H), 7.12 (dd, *J* = 8.9 Hz, 2.3 Hz, H-16, 1H), 6.82–6.77 (m, H-5, 1H), 6.52–6.47 (m, H-4, 1H), 4.64 (d, *J* = 13.8 Hz, H-6, 1H), 4.54 (d, *J* = 14.2 Hz, H-6, 1H), 3.90–3.93 (m, H-7, H-18, 4H), 3.66 (q, *J* = 6.9 Hz, H-10, 1H), 3.50 (d, *J* = 14.4 Hz, H-7, 1H), 1.39 (d, *J* = 7.1 Hz, H-11, 3H). ^13^C NMR (125 MHz, CD_3_OD) δ 178.6, 164.2 (dd, ^1^*J*_C*-*F_ = 247.6 Hz, ^3^*J*_C*-*F_ = 11.8 Hz), 160.7 (dd, ^1^*J*_C*-*F_ = 247.6 Hz, ^3^*J*_C*-*F_ = 11.8 Hz), 159.2, 151.4, 146.2, 137.6, 135.3, 131.4, 130.4, 130.3, 128.2, 127.0, 126.8, 125.0, 120.0, 111.8, 106.7, 104.9, 76.7, 57.1, 55.8, 47.7, 47.2, 18.7. ^19^F NMR (470 MHz, CD_3_OD) δ −109.27 (m, F_para_), −112.96 (m, F_ortho_).

#### Azole-Ketoprofen Hybrids (**13**, **14**)

Ketoprofen (122 mg, 0.48 mmol), HATU (311 mg, 0.82 mmol), DIPEA (0.28 mL, 1.61 mmol), and **1b-(*S*)** (102 mg, 0.40 mmol).

#### Azole-Ketoprofen Hybrid **13** (62 mg, 63%)

HRESI-MS *m/z* calculated for C_27_H_24_F_2_N_4_O_3_Na, 513.1714; found for [M + Na]^+^, 513.1713. ^1^H NMR (500 MHz, CD_3_OD) δ 8.31 (s, H-2, 1H), 7.78–7.75 (m, H-1, H-16, 3H), 7.67–7.60 (m, H-12, H-15, H-18, 3H), 7.55–7.52 (m, H-17, 2H), 7.44–7.39 (m, H-13, H-14, 2H), 7.36–7.31 (m, H-3, 1H), 6.85–6.80 (m, H-5, 1H), 6.69–6.65 (m, H-4, 1H), 4.67 (d, *J* = 14.3 Hz, H-6, 1H), 4.57 (d, *J* = 14.2 Hz, H-6, 1H), 3.94 (d, *J* = 14.2 Hz, H-7, 1H), 3.64 (q, *J* = 7.1 Hz H-10, 1H), 3.49 (d, *J* = 14.1 Hz, H-7, 1H), 1.34 (d, *J* = 7.1 Hz, H-11, 3H). ^13^C NMR (125 MHz, CD_3_OD) δ 198.5, 177.9, 164.3 (dd, ^1^*J*_C*-*F_ = 248.6 Hz, ^3^*J*_C*-*F_ = 12.4 Hz), 160.9 (dd, ^1^*J*_C*-*F_ = 246.6 Hz, ^3^*J*_C*-*F_ = 11.9 Hz), 151.6, 146.3, 143.3, 139.1, 139.0, 134.0, 132.9, 131.5, 131.2, 130.2, 130.0, 129.8, 129.7, 125.2, 112.0, 105.0, 76.8, 57.2, 47.8, 47.0, 19.0. ^19^F NMR (470 MHz, CD_3_OD) δ −109.08 (m, F_para_), −112.92 (m, F_ortho_).

#### Azole-Ketoprofen Hybrid **14** (61 mg, 62%)

HRESI-MS *m/z* calculated for C_27_H_25_F_2_N_4_O_3_, 491.1895; found for [M + H]^+^, 491.1890. ^1^H NMR (500 MHz, CD_3_OD) δ 8.32 (s, H-2, 1H), 7.78–7.74 (m, H-1, H-16, 3H), 7.67–7.60 (m, H-12, H-15, H-18, 3H), 7.55–7.52 (m, H-17, 2H), 7.44–7.38 (m, H-13, H-14, 2H), 7.29–7.24 (m, H-3, 1H), 6.85–6.80 (m, H-5, 1H), 6.66–6.61 (m, H-4, 1H), 4.60 (d, *J* = 14.2 Hz, H-6, 1H), 4.51 (d, *J* = 14.2 Hz, H-6, 1H), 3.76 (d, *J* = 14.2 Hz, H-7, 1H), 3.67–3.61 (m, H-7, H-10, 2H), 1.34 (d, *J* = 7.1 Hz, H-11, 3H). ^13^C NMR (125 MHz, CD_3_OD) δ 198.5, 178.6, 164.3 (dd, ^1^*J*_C*-*F_ = 247.2 Hz, ^3^*J*_C*-*F_ = 12.6 Hz), 160.7 (dd, ^1^*J*_C*-*F_ = 246.4 Hz, ^3^*J*_C*-*F_ = 11.7 Hz), 151.5, 146.4, 143.4, 139.1, 139.0, 134.0, 132.8, 131.5, 131.2, 130.1, 130.0, 129.9, 129.7, 125.3, 112.1, 105.0, 77.2, 57.2, 48.2, 46.9, 18.6. ^19^F NMR (470 MHz, CD_3_OD) δ −109.57 (m, F_para_), −112.92 (m, F_ortho_).

#### Azole-Ketoprofen Hybrids (**15**, **16**)

Ketoprofen (122 mg, 0.48 mmol), HATU (303 mg, 0.80 mmol), DIPEA (0.30 mL, 1.72 mmol), and **1b-(*R*)** (102 mg, 0.40 mmol).

#### Azole-Ketoprofen Hybrid **15** (84 mg, 86%)

HRESI-MS *m/z* calculated for C_27_H_25_F_2_N_4_O_3_, 491.1895; found for [M + H]^+^, 491.1897. ^1^H NMR (500 MHz, CD_3_OD) δ 8.31 (s, H-2, 1H), 7.79–7.76 (m, H-1, H-16, 3H), 7.68–7.61 (m, H-12, H-15, H-18, 3H), 7.56–7.53 (m, H-17, 2H), 7.44–7.39 (m, H-13, H-14, 2H), 7.36–7.31 (m, H-3, 1H), 6.86–6.81 (m, H-5, 1H), 6.70–6.66 (m, H-4, 1H), 4.68 (d, *J* = 14.3 Hz, H-6, 1H), 4.58 (d, *J* = 14.3 Hz, H-6, 1H), 3.94 (d, *J* = 14.2 Hz, H-7, 1H), 3.64 (q, *J* = 7.0 Hz, H-10, 1H), 3.50 (d, *J* = 14.1 Hz, H-7, 1H), 1.34 (d, *J* = 7.0 Hz, H-11, 3H). ^13^C NMR (125 MHz, CD_3_OD) δ 198.4, 177.9, 164.2 (dd, ^1^*J*_C*-*F_ = 247.2 Hz, ^3^*J*_C*-*F_ = 12.5 Hz), 160.8 (dd, ^1^*J*_C*-*F_ = 247.3 Hz, ^3^*J*_C*-*F_ = 11.5 Hz), 151.5, 146.2, 143.2, 139.0, 138.9, 134.0, 132.9, 131.4, 131.1, 130.1, 129.9, 129.6, 129.6, 125.1, 112.0, 105.0, 76.7, 57.1, 47.7, 46.9, 18.9. ^19^F NMR (470 MHz, CD_3_OD) δ −109.10 (m, F_para_), −112.94 (m, F_ortho_).

#### Azole-Ketoprofen Hybrid **16** (72 mg, 73%)

HRESI-MS *m/z* calculated for C_27_H_24_F_2_N_4_O_3_Na, 513.1714; found for [M + Na]^+^, 513.1717. ^1^H NMR (400 MHz, CD_3_OD) δ 8.32 (s, H-2, 1H), 7.79–7.74 (m, H-1, H-16, 3H), 7.68–7.60 (m, H-12, H-15, H-18, 3H), 7.56–7.51 (m, H-17, 2H), 7.45–7.38 (m, H-13, H-14, 2H), 7.30–7.24 (m, H-3, 1H), 6.86–6.80 (m, H-5, 1H), 6.66–6.61 (m, H-4, 1H), 4.60 (d, *J* = 14.3 Hz, H-6, 1H), 4.51 (d, *J* = 14.3 Hz, H-6, 1H), 3.76 (d, *J* = 14.3 Hz, H-7, 1H), 3.67–3.61 (m, H-7, H-10, 2H), 1.34 (d, *J* = 7.1 Hz, H-11, 3H). ^13^C NMR (100 MHz, CD_3_OD) δ 198.4, 178.5, 164.2 (dd, ^1^*J*_C*-*F_ = 247.2 Hz, ^3^*J*_C*-*F_ = 12.3 Hz), 160.6 (dd, ^1^*J*_C*-*F_ = 247.2 Hz, ^3^*J*_C*-*F_ = 12.3 Hz), 151.4, 146.3, 143.3, 139.0, 138.9, 133.9, 132.7, 131.4, 131.1, 130.0, 129.9, 129.8, 129.6, 125.2, 111.9, 104.9, 77.1, 57.1, 48.1, 46.8, 18.5. ^19^F NMR (375 MHz, CD_3_OD) δ −109.58 (m, F_para_), −112.95 (m, F_ortho_).

#### Azole-Niflumic Acid Hybrid (**17**)

Niflumic acid (72 mg, 0.26 mmol), HATU (152 mg, 0.40 mmol), DIPEA (0.14 mL, 0.80 mmol), and **1b-(*S*)** (50 mg, 0.20 mmol). Hybrid **17** (83 mg, 81%). HRESI-MS *m/z* calculated for C_24_H_19_F_5_N_6_O_2_Na, 541.1387; found for [M + Na]^+^, 541.1383. ^1^H NMR (400 MHz, CD_3_OD) δ 8.36 (s, H-2, 1H), 8.27 (dd, *J* = 4.9, 1.8 Hz, H-12, 1H), 8.16 (s, H-14, 1H), 7.84 (dd, *J* = 7.8, 1.8 Hz, H-10, 1H), 7.78 (s, H-1, 1H), 7.68 (d, *J* = 8.2 Hz, H-17, 1H), 7.53–7.46 (m, H-3, 1H), 7.42 (t, *J* = 8.0 Hz, H-16, 1H), 7.21 (d, *J* = 7.7 Hz, H-15, 1H), 6.97–6.91 (m, H-5, 1H), 6.82–6.77 (m, H-4, H-11, 2H), 4.82 (d, *J* = 14.4 Hz, H-6, 1H), 4.69 (d, *J* = 14.4 Hz, H-6, 1H), 3.98 (d, *J* = 14.2 Hz, H-7, 1H), 3.87 (d, *J* = 14.1 Hz, H-7, 1H). ^13^C NMR (100 MHz, CD_3_OD) δ 169.8, 163.0 (dd, ^1^*J*_C*-*F_ = 247.7 Hz, ^3^*J*_C*-*F_ = 12.3 Hz), 159.6 (dd, ^1^*J*_C*-*F_ = 246.8 Hz, ^3^*J*_C*-*F_ = 12.3 Hz), 154.3, 150.6, 150.1, 144.9, 141.0, 136.7, 130.7 (q, ^3^*J*_CF*3*_ = 31.7 Hz), 130.0, 129.1, 124.4 (d, ^1^*J*_CF*3*_ = 272.0 Hz), 124.1, 122.6, 117.8, 115.6, 113.9, 111.8, 110.6, 103.7, 75.6, 55.6, 46.6. ^19^F NMR (375 MHz, CD_3_OD) δ −64.16 (s, CF_3_), −108.55 (m, F_para_), −112.86 (m, F_ortho_).

#### Azole-Niflumic Acid Hybrid (**18**)

Niflumic acid (67 mg, 0.24 mmol), HATU (152 mg, 0.40 mmol), DIPEA (0.14 mL, 0.80 mmol), and **1b-(*R*)** (50 mg, 0.20 mmol). Hybrid **18** (90 mg, 89%). HRESI-MS *m/z* calculated for C_24_H_20_F_5_N_6_O_2_, 519.1568; found for [M + H]^+^, 519.1564. ^1^H NMR (500 MHz, CD_3_OD) δ 8.39 (s, H-2, 1H), 8.30 (dd, *J* = 4.8, 1.7 Hz, H-12, 1H), 8.19 (s, H-14, 1H), 7.87 (dd, *J* = 7.7, 1.6 Hz, H-10, 1H), 7.81 (s, H-1, 1H), 7.72 (d, *J* = 8.1 Hz, H-17, 1H), 7.55–7.50 (m, H-3, 1H), 7.46 (t, *J* = 8.0 Hz, H-16, 1H), 7.25 (d, *J* = 7.7 Hz, H-15, 1H), 7.00–6.95 (m, H-5, 1H), 6.85–6.81 (m, H-4, H-11, 2H), 4.85 (d, *J* = 14.3 Hz, H-6, 1H), 4.73 (d, *J* = 14.3 Hz, H-6, 1H), 4.02 (d, *J* = 14.1 Hz, H-7, 1H), 3.90 (d, *J* = 14.1 Hz, H-7, 1H). ^13^C NMR (125 MHz, CD_3_OD) δ 171.7, 164.8 (dd, ^1^*J*_C*-*F_ = 248.4 Hz, ^3^*J*_C*-*F_ = 12.7 Hz), 161.5 (dd, ^1^*J*_C*-*F_ = 246.8 Hz, ^3^*J*_C*-*F_ = 12.1 Hz), 156.2, 152.4, 151.9, 146.7, 142.8, 138.6, 132.5 (q, ^3^*J*_CF*3*_ = 31.9 Hz), 131.9, 130.9, 126.3 (d, ^1^*J*_CF*3*_ = 271.6 Hz), 125.9, 124.4, 119.6, 117.5, 115.7, 113.6, 112.5, 105.5, 77.4, 57.5, 48.4. ^19^F NMR (470 MHz, CD_3_OD) δ −64.19 (s, CF_3_), −108.57 (m, F_para_), −112.88 (m, F_ortho_).

#### Azole-Diflunisal Hybrid (**19**)

Diflunisal (63 mg, 0.25 mmol) was dissolved in dry DMF (2 mL) under argon at 0 °C and then treated with HATU (151 mg, 0.40 mmol) and stirred for 10 min at 0 °C. To the reaction mixture, **1b-(*S*)** (50 mg, 0.20 mmol) was added, and the solution was stirred at room temperature. The reaction was monitored using TLC (MeOH/DCM, 1:9). Upon completion at 3 h, the product was extracted with ethyl acetate, washed with H_2_O, dried over MgSO_4_, and concentrated to give the crude enantiomer. The concentrated crude was first purified by flash column chromatography on SiO_2_ using a gradient of MeOH/DCM as eluent and then by preparative RP-HPLC to afford hybrid **19** (22 mg, 23%). HRESI-MS *m/z* calculated for C_24_H_18_F_4_N_4_O_3_Na, 509.1213; found for [M + Na]^+^, 509.1207. ^1^H NMR (500 MHz, CD_3_OD) δ 8.35 (s, H-2, 1H), 7.90 (dd, *J* = 2.2, 1.0 Hz, H-13, 1H), 7.77 (s, H-1, 1H), 7.53–7.48 (m, H-12, H-14, 2H), 7.41–7.46 (m, H-3, 1H), 7.03–6.98 (m, H-15, H-16, 2H), 6.97–6.92 (m, H-5, H-11, 2H), 6.86–6.82 (m, H-4, 1H), 4.81 (d, *J* = 14.4 Hz, H-6, 1H), 4.68 (d, *J* = 14.4 Hz, H-6, 1H), 4.02 (d, *J* = 14.1 Hz, H-7, 1H), 3.94 (d, *J* = 14.4 Hz, H-7, 1H). ^13^C NMR (125 MHz, CD_3_OD) δ 171.0, 164.6 (dd, ^1^*J*_C*-*F_ = 248.0 Hz, ^3^*J*_C*-*F_ = 12.4 Hz), 163.8 (dd, ^1^*J*_C*-*F_ = 247.5 Hz, ^3^*J*_C*-*F_ = 12.0 Hz), 161.3 (dd, ^1^*J*_C*-*F_ = 248.8 Hz, ^3^*J*_C*-*F_ = 12.0 Hz), 161.1 (dd, ^1^*J*_C*-*F_ = 247.1 Hz, ^3^*J*_C*-*F_ = 12.0 Hz), 160.0, 151.6, 146.4, 135.4, 132.7, 131.6, 130.8, 127.6, 126.1, 125.6, 118.7, 118.0, 112.8, 112.3, 105.2, 76.9, 57.3, 48.0. ^19^F NMR (470 MHz, CD_3_OD) δ −109.05 (m, F_para_), −112.97 (m, F_ortho_), −113.84 (m, F_para_), −115.49 (m, F_ortho_).

Azole-COX inhibitor hybrids **20–24** were prepared in the same manner as hybrid **19** with the following modifications:

#### Azole-Diflunisal Hybrid (**20**)

Diflunisal (60 mg, 0.24 mmol), HATU (152 mg, 0.40 mmol), and **1b-(*R*)** (50 mg, 0.20 mmol). Hybrid **20** (31 mg, 32%). HRESI-MS *m/z* calculated for C_24_H_18_F_4_N_4_O_3_Na, 509.1213; found for [M + Na]^+^, 509.1204. ^1^H NMR (400 MHz, CD_3_OD) δ 8.36 (s, H-2, 1H), 7.92–7.91 (m, H-13, 1H), 7.79 (s, H-1, 1H), 7.55–7.42 (m, H-3, H-12, H-14, 3H), 7.04–6.92 (m, H-5, H-11, H-15, H-16, 4H), 6.88–6.82 (m, H-4, 1H), 4.82 (d, *J* = 14.3 Hz, H-6, 1H), 4.69 (d, *J* = 14.3 Hz, H-6, 1H), 4.02 (d, *J* = 14.0 Hz, H-7, 1H), 3.96 (d, *J* = 14.2 Hz, H-7, 1H). ^13^C NMR (100 MHz, CD_3_OD) δ 170.7, 164.5 (dd, ^1^_C*-*F_ = 247.6 Hz, ^3^*J*_C*-*F_ = 12.5 Hz), 163.7 (dd, ^1^*J*_C*-*F_ = 247.6 Hz, ^3^*J*_C*-*F_ = 11.7 Hz), 161.0 (d, ^1^*J*_C*-*F_ = 247.0 Hz), 160.9 (d, ^1^*J*_C*-*F_ = 247.0 Hz), 159.6, 151.5, 146.2, 135.4, 132.7, 132.1, 130.7, 127.6, 125.8, 125.5, 118.4, 117.9, 112.7, 112.2, 105.2, 76.7, 57.2, 48.8. ^19^F NMR (375 MHz, CD_3_OD) δ −109.22 (m, F_para_), −113.12 (m, F_ortho_), −113.95 (m, F_para_), −115.66 (m, F_ortho_).

#### Azole-Salicylic Acid Hybrid (**21**)

Salicylic acid (35 mg, 0.25 mmol), HATU (152 mg, 0.40 mmol), and **1b-(*S*)** (50 mg, 0.20 mmol). Hybrid **21** (24 mg, 32%). HRESI-MS *m/z* calculated for C_18_H_16_F_2_N_4_O_3_Na, 397.1088; found for [M + Na]^+^, 397.1081. ^1^H NMR (400 MHz, CD_3_OD) δ 8.36 (s, H-2, 1H), 7.78 (s, H-1, 1H), 7.73 (dd, *J* = 8.3, 1.7 Hz, H-10, 1H), 7.54–7.47 (m, H-3, 1H), 7.37–7.32 (m, H-12, 1H), 6.98–6.92 (m, H-5, 1H), 6.89–6.81 (m, H-4, H-11, H-13, 3H), 4.82 (d, *J* = 14.4 Hz, H-6, 1H), 4.68 (d, *J* = 14.4 Hz, H-6, 1H), 4.00 (d, *J* = 14.0 Hz, H-7, 1H), 3.94 (d, *J* = 14.2 Hz, H-7, 1H). ^13^C NMR (100 MHz, CD_3_OD) δ 169.9, 163.1 (dd, ^1^*J*_C*-*F_ = 248.4 Hz, ^3^*J*_C*-*F_ = 12.6 Hz), 159.6 (dd, ^1^*J*_C*-*F_ = 246.8 Hz, ^3^*J*_C*-*F_ = 11.8 Hz), 158.8, 150.1, 144.9, 133.6, 130.1, 128.7, 124.2, 119.1, 116.9, 116.2, 110.8, 103.7, 75.4, 55.8, 46.4. ^19^F NMR (375 MHz, CD_3_OD) δ −109.25 (m, F_para_), −113.21 (m, F_ortho_).

#### Azole-Salicylic Acid Hybrid (**22**)

Salicylic acid (33 mg, 0.24 mmol), HATU (150 mg, 0.40 mmol), and **1b-(*R*)** (50 mg, 0.20 mmol). Hybrid **22** (24 mg, 32%). HRESI-MS *m/z* calculated for C_18_H_16_F_2_N_4_O_3_Na, 397.1088; found for [M + Na]^+^, 397.1089. ^1^H NMR (400 MHz, CD_3_OD) δ 8.36 (s, H-2, 1H), 7.78 (s, H-1, 1H), 7.73 (dd, *J* = 8.2, 1.7 Hz, H-10, 1H), 7.54–7.47 (m, H-3, 1H), 7.37–7.32 (m, H-12, 1H), 6.98–6.92 (m, H-5, 1H), 6.89–6.81 (m, H-4, H-11, H-13, 3H), 4.82 (d, *J* = 14.3 Hz, H-6, 1H), 4.68 (d, *J* = 14.4 Hz, H-6, 1H), 4.00 (d, *J* = 14.2 Hz, H-7, 1H), 3.94 (d, *J* = 14.1 Hz, H-7, 1H). ^13^C NMR (100 MHz, CD_3_OD) δ 171.2, 164.4 (dd,^1^*J*_C*-*F_ = 248.2 Hz,^3^*J*_C*-*F_ = 12.8 Hz), 160.9 (dd,^1^*J*_C*-*F_ = 247.1 Hz,^3^*J*_C*-*F_ = 12.1 Hz), 160.1, 151.4, 146.2, 135.0, 131.5, 130.1, 125.5, 120.4, 118.2, 117.5, 112.1, 105.1, 76.7, 57.2, 47.8. ^19^F NMR (375 MHz, CD_3_OD) δ −109.23 (m, F_para_), −113.19 (m, F_ortho_).

#### Azole-Diclofenac Hybrid (**23**)

Diclofenac (72 mg, 0.24 mmol), HATU (152 mg, 0.40 mmol), and **1b-(*S*)** (51 mg, 0.20 mmol). Hybrid **23** (50 mg, 48%). HRESI-MS *m/z* calculated for C_25_H_21_Cl_2_F_2_N_5_O_2_Na, 554.0938; found for [M + Na]^+^, 554.0944. ^1^H NMR (400 MHz, CD_3_OD) δ 8.33 (s, H-2, 1H), 7.79 (s, H-1, 1H), 7.41 (d, *J* = 8.2 Hz, H-16, 2H), 7.37–7.30 (m, H-3, 1H), 7.10–6.98 (m, H-11, H-13, H-17, 3H), 6.87–6.78 (m, H-5, H-12, 2H), 6.61–6.55 (m, H-4, 1H), 6.33 (d, *J* = 7.9 Hz, H-14, 1H), 4.66 (d, *J* = 14.3 Hz, H-6, 1H), 4.59 (d, *J* = 14.4 Hz, H-6, 1H), 3.81 (d, *J* = 14.3 Hz, H-7, 1H), 3.67 (d, *J* = 14.4 Hz, H-7, 1H), 3.58 (d, *J* = 13.8 Hz, H-10, 1H), 3.53 (d, *J* = 13.6 Hz, H-10, 1H). ^13^C NMR (100 MHz, CD_3_OD) δ 176.5, 164.2 (dd, ^1^*J*_C*-*F_ = 248.3 Hz, ^3^*J*_C*-*F_ = 12.3 Hz), 160.6 (dd, ^1^*J*_C*-*F_ = 246.4 Hz, ^3^*J*_C*-*F_ = 12.3 Hz), 151.4, 146.3, 144.4, 139.2, 131.5, 131.3, 130.1, 128.7, 126.4, 125.7, 125.0, 122.5, 118.1, 112.0, 104.9, 76.9, 57.1, 48.2, 40.4. ^19^F NMR (375 MHz, CD_3_OD) δ −109.69 (m, F_para_), −113.00 (m, F_ortho_).

#### Azole-Diclofenac Hybrid (**24**)

Diclofenac (67 mg, 0.23 mmol), HATU (144 mg, 0.38 mmol), and **1b-(*R*)** (50 mg, 0.20 mmol). Hybrid **24** (52 mg, 50%). HRESI-MS *m/z* calculated for C_25_H_21_Cl_2_F_2_N_5_O_2_Na, 554.0938; found for [M + Na]^+^, 554.0940. ^1^H NMR (400 MHz, CD_3_OD) δ 8.33 (s, H-2, 1H), 7.79 (s, H-1, 1H), 7.41 (d, *J* = 8.1 Hz, H-16, 2H), 7.37–7.30 (m, H-3, 1H), 7.10–6.98 (m, H-11, H-13, H-17, 3H), 6.87–6.78 (m, H-5, H-12, 2H), 6.61–6.55 (m, H-4, 1H), 6.33 (d, *J* = 7.9 Hz, H-14, 1H), 4.67 (d, *J* = 14.3 Hz, H-6, 1H), 4.59 (d, *J* = 14.3 Hz, H-6, 1H), 3.81 (d, *J* = 14.3 Hz, H-7, 1H), 3.67 (d, *J* = 14.3 Hz, H-7, 1H), 3.58 (d, *J* = 13.7 Hz, H-10, 1H), 3.53 (d, *J* = 13.7 Hz, H-10, 1H).^13^C NMR (100 MHz, CD_3_OD) δ 176.5, 164.2 (dd, ^1^*J*_C*-*F_ = 247.2 Hz, ^3^*J*_C*-*F_ = 12.0 Hz), 160.6 (dd, ^1^*J*_C*-*F_ = 246.0 Hz, ^3^*J*_C*-*F_ = 12.03 Hz), 151.4, 146.3, 144.4, 139.2, 131.5, 131.3, 130.1, 128.7, 126.4, 125.7, 125.0, 122.5, 118.1, 112.0, 104.9, 76.9, 57.1, 48.2, 40.4. ^19^F NMR (375 MHz, CD_3_OD) δ −109.68 (m, F_para_), −112.94 (m, F_ortho_).

### Biological Assays

#### Preparation of Stock Solutions of the Tested Compounds

Hybrids **1**–**24** were dissolved in anhydrous DMSO to final concentrations of 5 mg/mL. The antifungal drugs FLC and VOR were purchased from Sigma Aldrich were dissolved in anhydrous DMSO to final concentrations of 5 mg/mL.

#### Minimal Inhibitory Concentration Broth Double-Dilution Assay

*C. auris* minimal inhibitory concentrations (MICs) were determined using CLSI M27-A3 guidelines with minor modifications. Starter cultures were streaked from glycerol stock onto YPAD agar plates and grown for 24 h at 37 °C. Colonies were suspended in 1 mL of PBS and diluted to 1 × 10^–3^ optical density at 600 nm (OD_600_) and then diluted 1:100 into fresh medium. Hybrids dissolved in DMSO were added to YPAD broth (32 μL of stock solution in 1218 μL of YPAD broth), and serial double dilutions of hybrids in YPAD were prepared in flat-bottomed 96-well microplates (Corning) to enable testing of concentrations ranging from 64 to 0.007 μg/mL. Control wells with yeast cells but no-drug and blank wells containing only YPAD were prepared. An equal volume (100 μL) of yeast suspension in YPAD broth was added to each well with the exceptions of the blank wells. After incubation for 24 h at 37 °C, MTT (50 μL of a 1 mg/mL solution in ddH_2_O) was added to each well followed by additional incubation at 37 °C for 2 h. MIC values (Table S3) were defined as the lowest concentration of an antifungal agent that caused a specified reduction in visible growth as per the CLSI M27-A3 protocol. The magnitude of reduction in visible growth was assessed using the following numerical scale: 0, optically clear; 1, slightly hazy; 2, prominent decrease (∼50%) in visible growth; 3, slight reduction in visible growth; and 4, no reduction in visible growth. The MIC was defined based on a reduction in growth to 0 or 1. Results were confirmed in two independent experiments, and each concentration was tested in triplicate. FLC and VOR were used as control drugs.

*C. albicans*, *C. glabrata*, *C. parapsilosis*, *C. guilliermondii*, *C. tropicalis*, and *C. dubliniensis* MICs were determined using CLSI M27-A3 guidelines with minor modifications. Starter cultures were streaked from glycerol stock onto YPAD agar plates and grown for 24 h at 30 °C. Colonies were suspended in 1 mL PBS and diluted to 1 × 10^–3^ OD_600_ and then diluted 1:100 into fresh medium. Hybrids dissolved in DMSO were added to YPAD broth (32 μL of stock solution in 1218 μL of YPAD broth), and serial double dilutions of hybrids in YPAD were prepared in flat-bottomed 96-well microplates (Corning) to enable testing of concentrations ranging from 64 to 0.003 μg/mL. Control wells with yeast cells but no-drug and blank wells containing only YPAD were prepared. An equal volume (100 μL) of yeast suspensions in YPAD broth was added to each well with the exceptions of the blank wells. MIC values (Tables S4–S6) were determined after 24 h at 30 °C by measuring the OD_600_ using a plate reader (Infinite M200 PRO, Tecan). MIC values were defined as the point at which the OD_600_ was reduced by ≥80% compared to the no-drug wells. Each concentration was tested in triplicate, and results were confirmed by two independent sets of experiments. FLC and VOR were used as control drugs.

#### Disk Diffusion Assay

Antifungal activities of select hybrids against *C. albicans* SN152, *C. parapsilosis* ATCC 22019, and *C. tropicalis* 660 were confirmed by the disk diffusion assay. Strains were streaked from frozen culture onto YPAD agar and incubated for 24 h at 30 °C. Two or three colonies were placed into 1 mL of PBS solution, and OD_600_ was determined with a TECAN Infinite. OD_600_ was adjusted to 0.02 for *C. albicans* SN152 and to 0.025 for *C. parapsilosis* ATCC 22019 and *C. tropicalis* 660 by dilution with PBS. Aliquots of 200 μL of the diluted cultures of each strain were plated onto 15-mL casitone agar plates and spread using sterile beads (3 mm, Fisher Scientific). After the plates dried, a single disk (6-mm diameter, Becton Dickinson) with 25 μg of the hybrid being tested was placed in the center of each plate. Plates were then incubated at 30 °C and photographed under the same imaging conditions after 24 and 48 h. FLC and VOR were used as control drugs.

#### Growth Curve Analyses

Growth curves were determined using the double-dilution method in 96-well plates. Starter cultures were streaked from glycerol stock onto YPAD agar plates and grown for 24 h at 30 °C. Colonies were suspended in 1 mL of PBS and diluted to 1 × 10^–3^ OD_600_ and then diluted 1:100 into fresh medium. Hybrids dissolved in DMSO were added to YPAD broth (32 μL of stock solution in 1218 μL of YPAD broth), and serial double dilutions of hybrids in YPAD were prepared in flat-bottomed 96-well microplates (Corning) to enable testing of concentrations ranging from 64 to 1 μg/mL. Control wells with yeast cells but no-drug (100% growth) and blank wells containing only YPAD (0% growth) were prepared. An equal volume (100 μL) of yeast suspensions in YPAD broth was added to each well with the exceptions of the blank wells. Growth was determined at 30 °C by measuring the OD_600_ using a plate reader (Infinite M200 PRO, Tecan) every 40 min over 48 h. Each concentration was tested in triplicate, and results were confirmed by two independent sets of experiments. FLC and VOR were used as control drugs.

## Supporting Information Available

Proton numbering systems, infromation and procedures for the preparation of intermediate compounds and the crystallized compounds, chiral semi-preparative HPLC data; yeast strains, MIC tables, NMR spectra (PDF)
SMILES dataset (CSV)

## Abbreviations

COX: cyclooxygenase
CYP51: cytochrome P450 (Lanosterol 14α-demethylase)
DCM: dichloromethane
DIPEA: diisopropylethylamine
DMF: dimethylformamide
DMSO: dimethylsulfoxide
FDA: food and drug administration
FLC: fluconazole
HATU: hexafluorophosphate azabenzotriazole tetramethyl uranium
MIC: minimal inhibitory concentration
MTT: 3-(4,5-dimethylthiazol-2-yl)-2,5-diphenyl-2H-tetrazolium bromide
OD: optical density
PGE_2_: prostaglandin E2
PBS: phosphate buffered saline
VOR: voriconazole
TFA: trifluoroacetic acid
YPAD: yeast extract peptone (adenine) dextrose
