# Supplementary material for: Fluconazole-COX Inhibitor Hybrids: A Dual-Acting Class of Antifungal Azoles
Source: J Med Chem. 2022 Jan 27;65(3):2361–73. doi: 10.1021/acs.jmedchem.1c01807 (PMC8842223; doi:10.1021/acs.jmedchem.1c01807)
Supplement: Supplementary file 1 — jm1c01807_si_001.pdf [file jm1c01807_si_001.pdf]

Supporting Information

Fluconazole-COX Inhibitor Hybrids: A Dual-Acting Class of Antifungal Azoles

Rebecca Elias,[a] *Pallabita Basu,*[a] *and Micha Fridman*[a,*]

[a] School of Chemistry, Raymond & Beverly Sackler Faculty of Exact Sciences, Tel Aviv University, Tel Aviv, 6997801, Israel.

*Correspondence to: mfridman@tauex.tau.ac.il, +972-3-6408687.

Table of content

1. Proton numbering systems 3

2. Synthetic procedures 4

3. Analytical chiral HPLC chromatograms 5

4. Compound purity information 19

5. Biological information 20

   A. Yeast strains 20

   B. Minimal inhibitory concentrations (MICs) tables 21

6. References 25

7. NMR spectra 27

1. Proton numbering systems

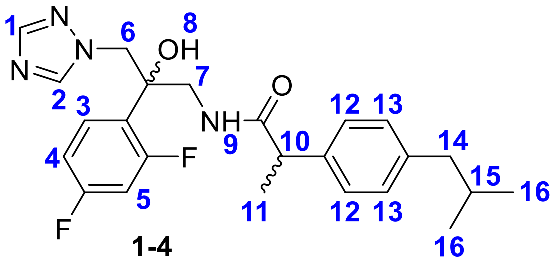

1-4

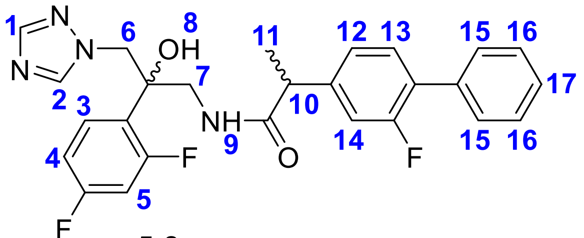

5-8

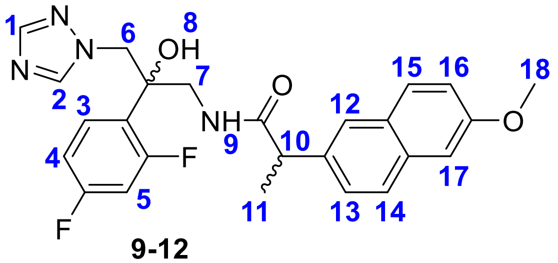

9-12

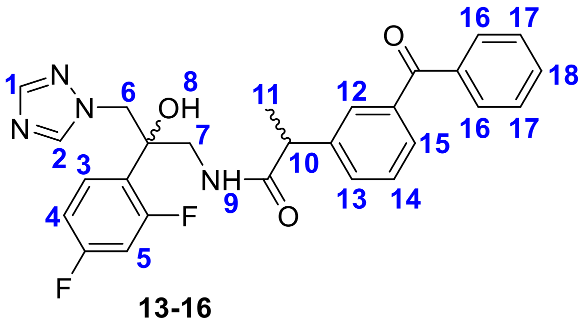

13-16

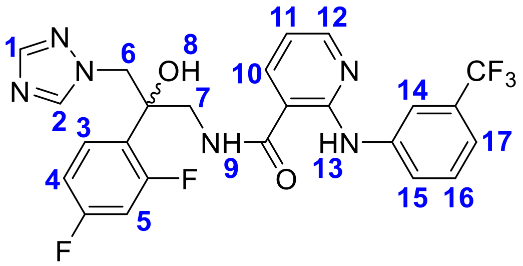

17, 18

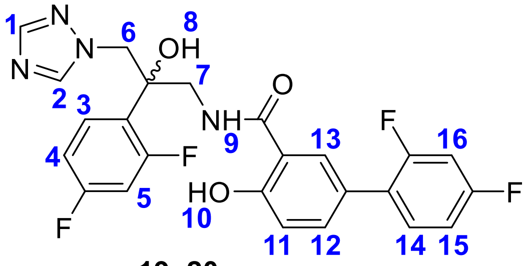

19, 20

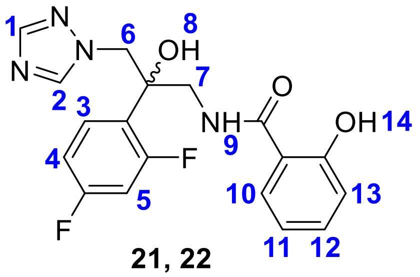

21, 22

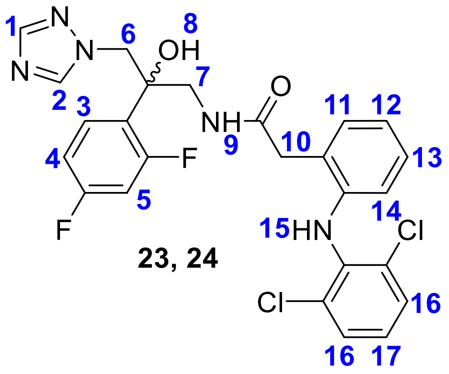

23, 24

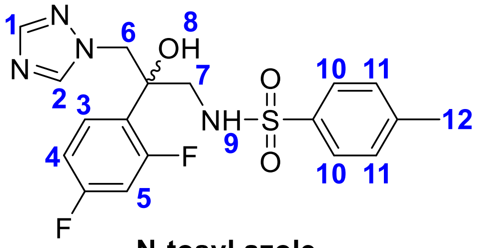

N-tosyl azole

Figure S1. Synthesized hybrids with protons numbered.

2. Synthetic procedures

Racemates 1a, 1b. The compounds were synthesized following a previously reported procedure.[1]

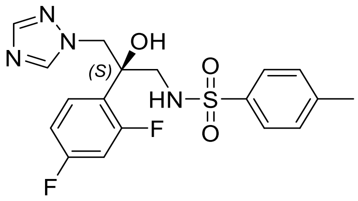

N-tosyl azole (*S*). 1b-(*R*) (40 mg, 0.16 mmol) was dissolved in dry pyridine (2 mL) under argon at 0 °C then treated with tosyl chloride (39 mg, 0.20 mmol) and stirred at 0 °C. The reaction was monitored by TLC (petroleum ether/ethyl acetate, 1:1). Upon completion at 2 h, the product was extracted with ethyl acetate, washed with H_2O, dried over $MgSO_4$, and concentrated to give the crude enantiomer. The concentrated crude was purified by preparative RP-HPLC to afford the compound **N-tosyl azole (*S*)** (51 mg, 80%). ^{1}H NMR (400 MHz, CD_3OD) δ 8.30 (s, H-2, 1H), 7.74 (s, H-1, 1H), 7.66 (d, *J* = 8.5, H-10, 2H), 7.43 - 7.32 (m, H-3, H-11, 3H), 6.86 - 6.78 (m, H-4, H-5, 2H), 4.72 (d, *J* = 14.4 Hz, H-6, 1H), 4.64 (d, *J* = 14.4 Hz, H-6, 1H), 3.38 (d, *J* = 13.7 Hz, H-7, 1H), 3.33 (d, *J* = 13.7 Hz, H-7, 1H), 2.42 (s, H-12, 3H). ^{13}C NMR (100 MHz, CD_3OD) δ 164.5 (dd, $^1J_{C\text{-}F}$ = 247.7 Hz, $^3J_{C\text{-}F}$ = 13.2 Hz), 160.8 (dd, $^1J_{C\text{-}F}$ = 246.0 Hz, $^3J_{C\text{-}F}$ = 11.6 Hz), 151.5, 146.3, 145.0, 138.9, 131.6, 131.0, 128.2, 125.1, 112.2, 105.2, 75.8, 56.7, 50.7, 21.6. ^{19}F NMR (375 MHz, CD_3OD) δ -109.30 (m, F_{para}), -113.00 (m, F_{ortho}).

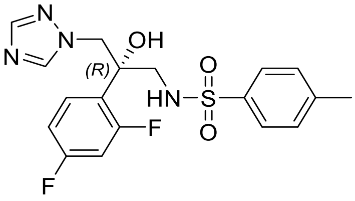

N-tosyl azole (*R*). 1b-(*S*) (20 mg, 0.08 mmol) was dissolved in dry pyridine (1 mL) under argon at 0 °C then treated with tosyl chloride (20 mg, 0.10 mmol) and stirred at 0 °C. The reaction was monitored by TLC (petroleum ether/ethyl acetate, 1:1). Upon completion at 2 h, the product was extracted with ethyl acetate, washed with H_2O, dried over $MgSO_4$, and concentrated to give the crude enantiomer. The concentrated crude was purified by preparative RP-HPLC to afford the compound **N-tosyl azole (*R*)** (26 mg, 81%). ^{1}H NMR (400 MHz, CD_3OD) δ 8.39 (s, H-2, 1H), 7.80 (s, H-1, 1H), 7.66 (d, *J* = 8.1, H-10, 2H), 7.43 -

7.34 (m, H-3, H-11, 3H), 6.88 - 6.80 (m, H-4, H-5, 2H), 4.72 (d, J = 14.6 Hz, H-6, 1H), 4.64 (d, J = 14.6 Hz, H-6, 1H), 3.38 (d, J = 13.6 Hz, H-7, 1H), 3.33 (d, J = 13.6 Hz, H-7, 1H), 2.43 (s, H-12, 3H). ^{13}C NMR (100 MHz, CD_3OD) δ 164.5 (dd, $^{1}J_{C-F}$ = 248.6 Hz, $^{3}J_{C-F}$ = 12.52 Hz), 160.7 (dd, $^{1}J_{C-F}$ = 247.0 Hz, $^{3}J_{C-F}$ = 12.5 Hz), 151.1, 146.1, 145.0, 138.9, 131.6, 130.9, 128.2, 125.1, 112.2, 105.2, 75.7, 56.8, 50.7, 21.6. ^{19}F NMR (375 MHz, CD_3OD) δ -109.34 (m, F_{para}), -113.04 (m, F_{ortho}).

3. Analytical chiral HPLC chromatograms

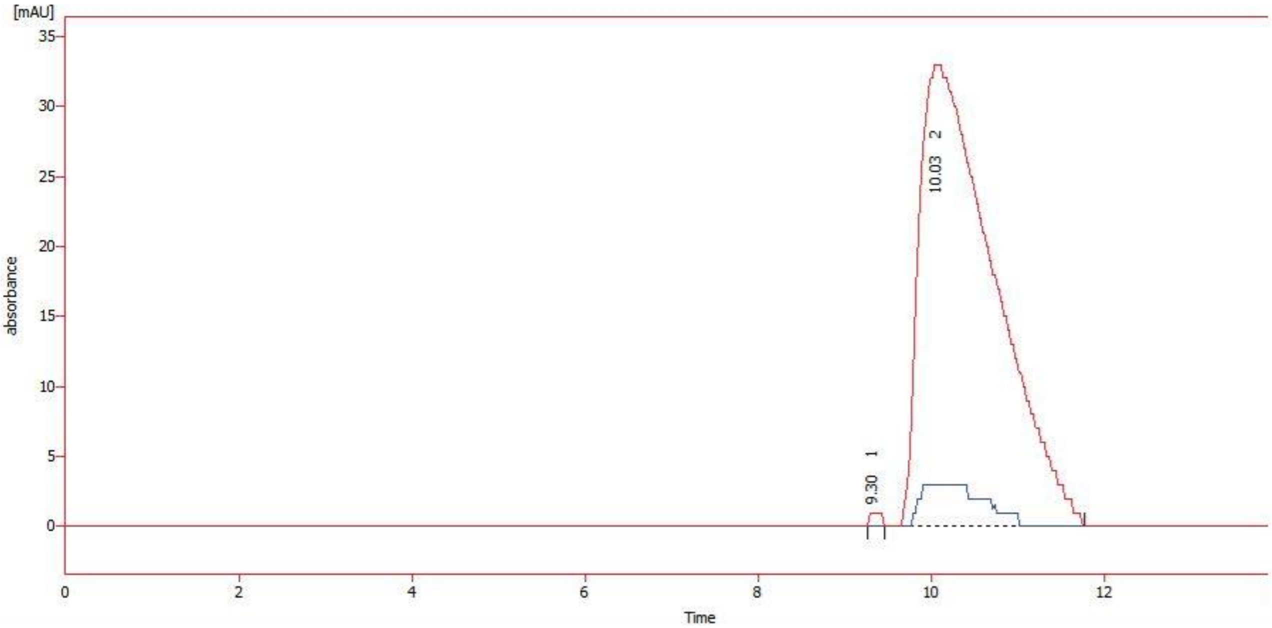

Figure S2. Chiral semi-preparative chromatogram of hybrid **1**. The mobile phase was 60% acetonitrile and 40% H_2O (isocratic). Red line corresponds to UV 210 nm. Blue line corresponds to UV 260 nm.

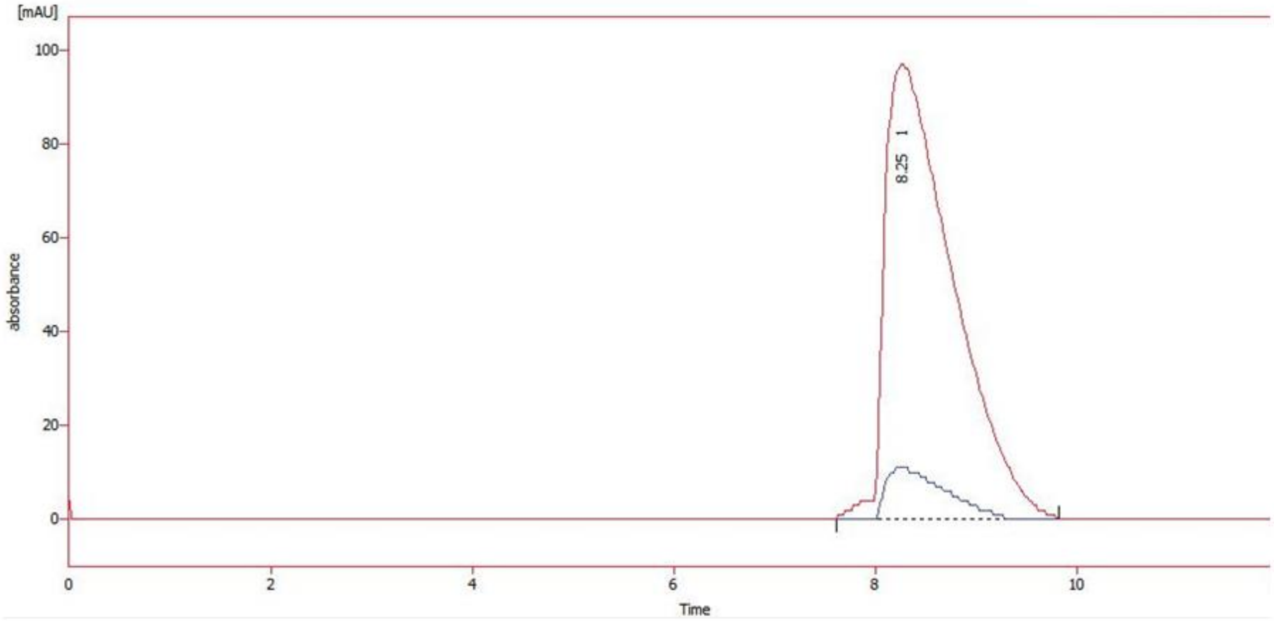

Figure S3. Chiral semi-preparative chromatogram of hybrid **2**. The mobile phase was 60% acetonitrile and 40% H_2O (isocratic). Red line corresponds to UV 210 nm. Blue line corresponds to UV 260 nm.

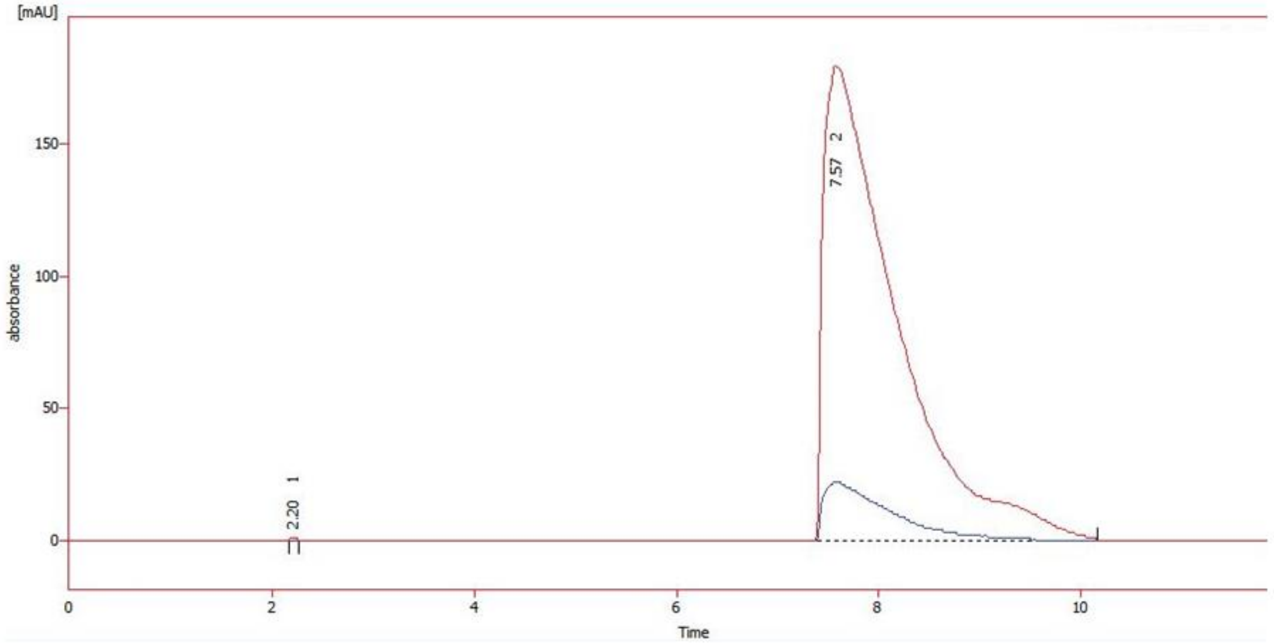

Figure S4. Chiral semi-preparative chromatogram of hybrid **3**. The mobile phase was 60% acetonitrile and 40% H_2O (isocratic). Red line corresponds to UV 210 nm. Blue line corresponds to UV 260 nm.

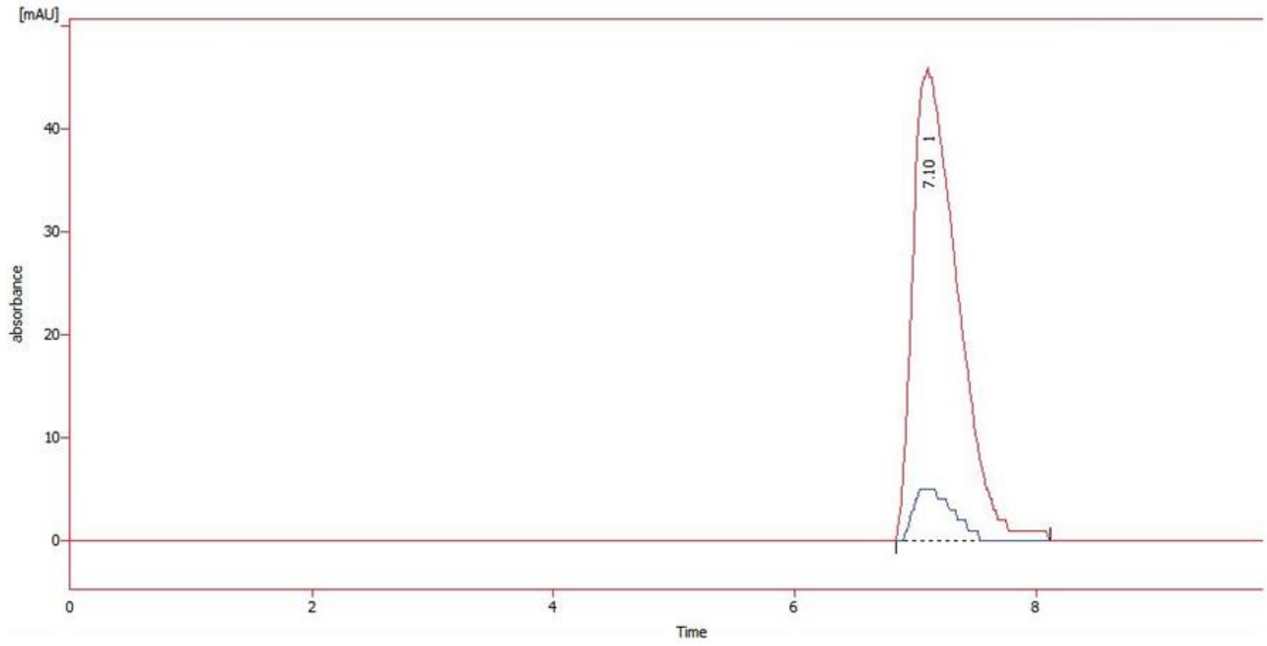

Figure S5. Chiral semi-preparative chromatogram of hybrid **4**. The mobile phase was 60% acetonitrile and 40% H_2O (isocratic). Red line corresponds to UV 210 nm. Blue line corresponds to UV 260 nm.

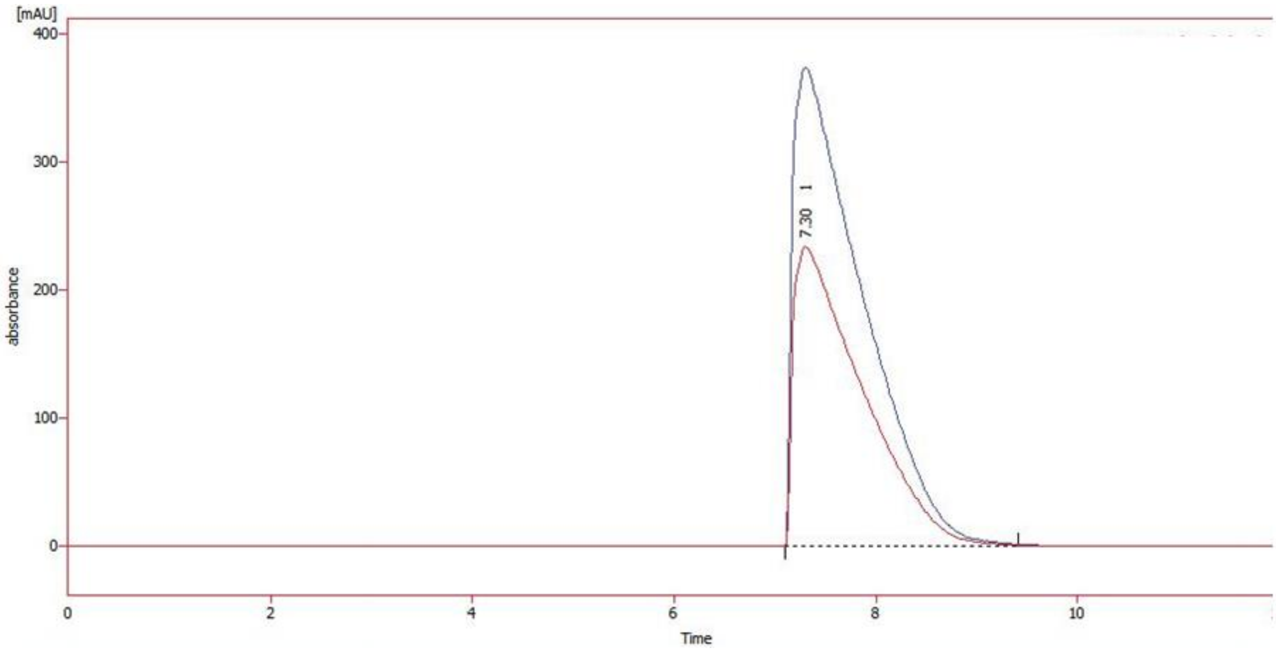

Figure S6. Chiral semi-preparative chromatogram of hybrid **5**. The mobile phase was 60% acetonitrile and 40% H_2O (isocratic). Red line corresponds to UV 210 nm. Blue line corresponds to UV 260 nm.

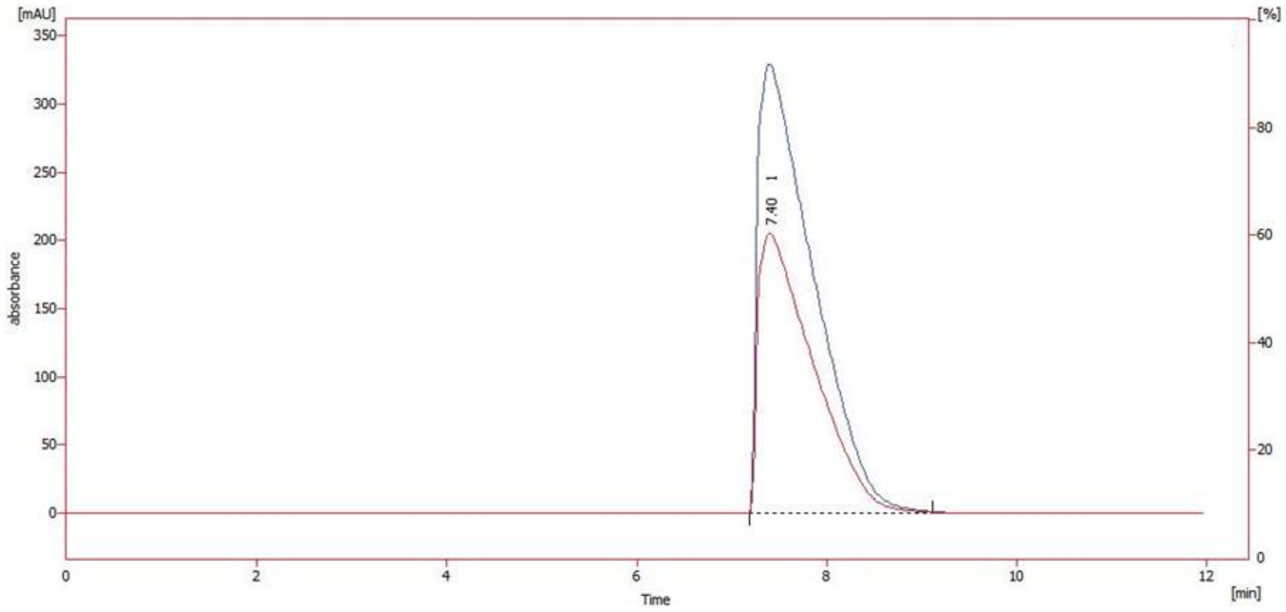

Figure S7. Chiral semi-preparative chromatogram of hybrid **6**. The mobile phase was 60% acetonitrile and 40% H_2O (isocratic). Red line corresponds to UV 210 nm. Blue line corresponds to UV 260 nm.

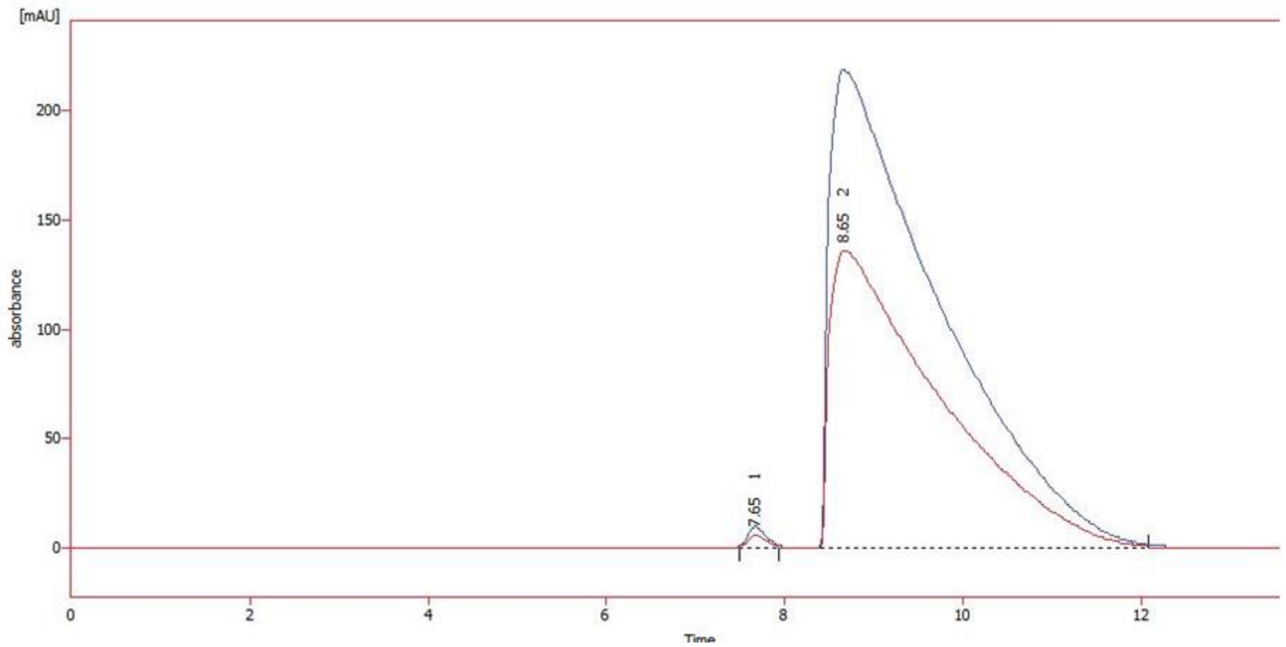

Figure S8. Chiral semi-preparative chromatogram of hybrid **7**. The mobile phase was 60% acetonitrile and 40% H_2O (isocratic). Red line corresponds to UV 210 nm. Blue line corresponds to UV 260 nm.

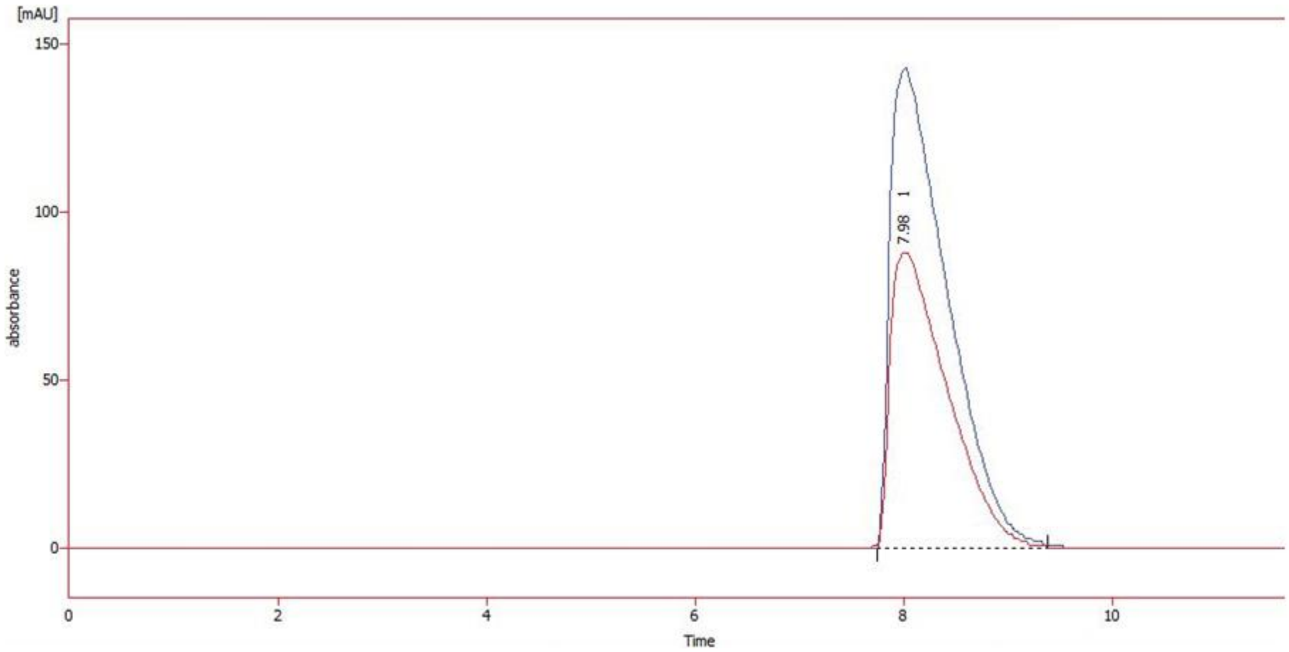

Figure S9. Chiral semi-preparative chromatogram of hybrid **8**. The mobile phase was 60% acetonitrile and 40% H_2O (isocratic). Red line corresponds to UV 210 nm. Blue line corresponds to UV 260 nm.

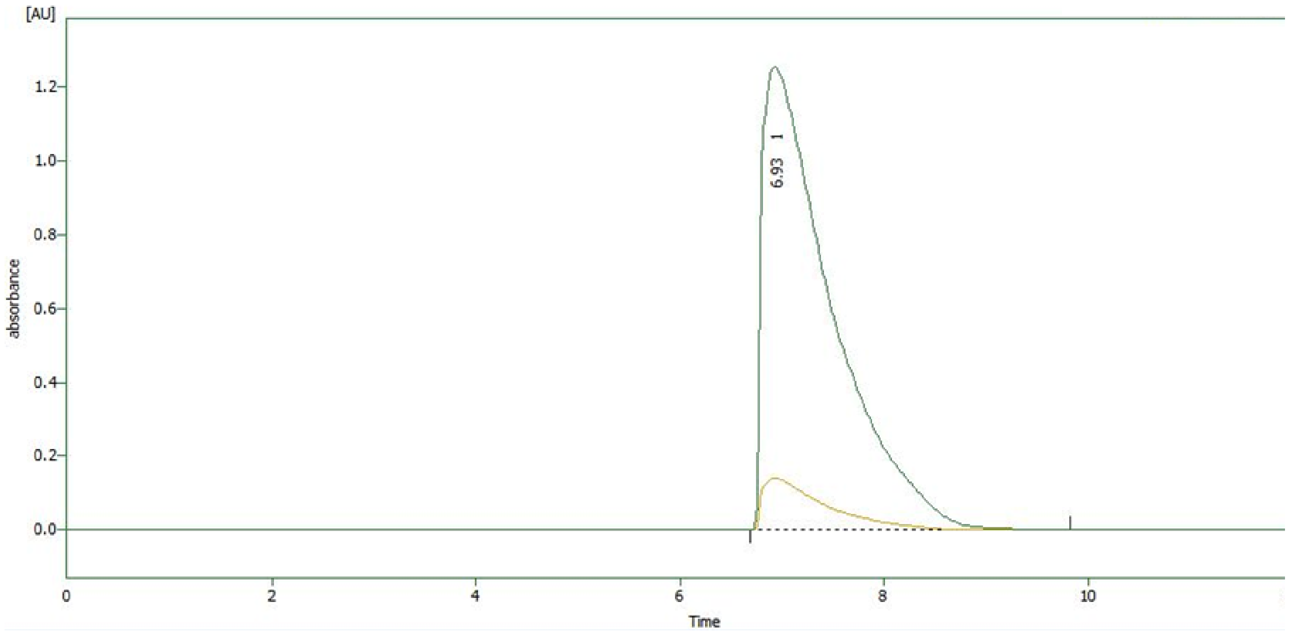

Figure S10. Chiral semi-preparative chromatogram of hybrid **9**. The mobile phase was 60% acetonitrile and 40% H_2O (isocratic). Green line corresponds to UV 210 nm. Yellow line corresponds to UV 260 nm.

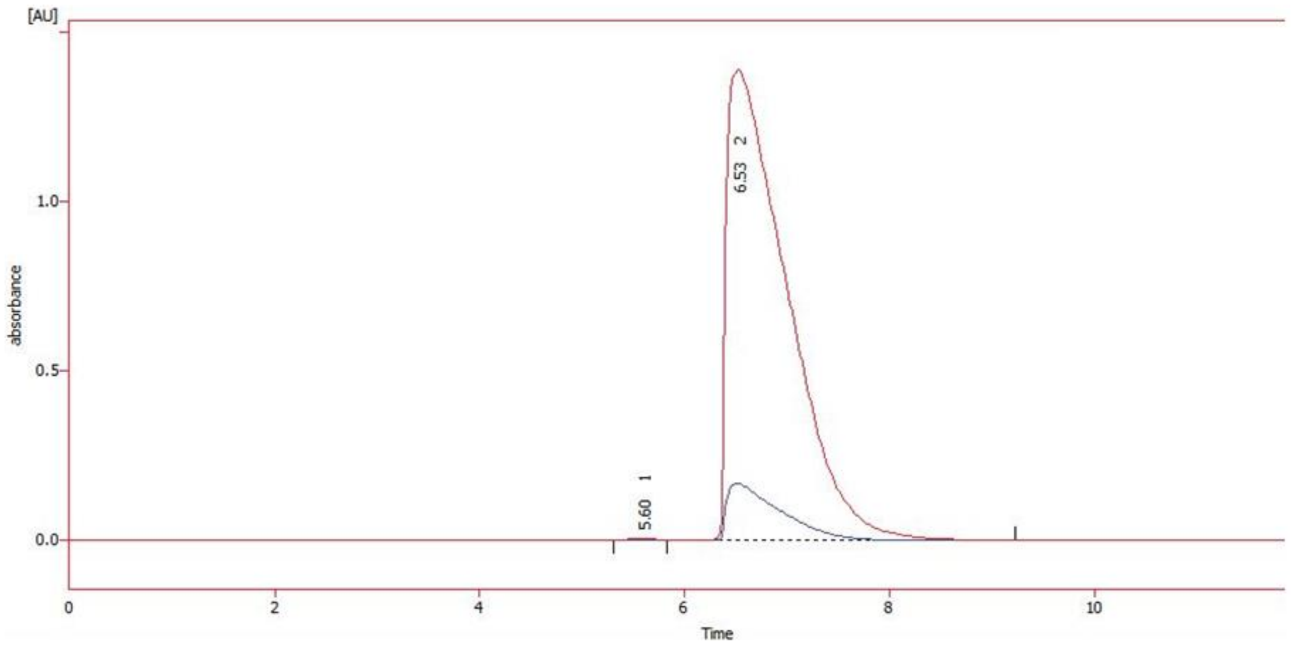

Figure S11. Chiral semi-preparative chromatogram of hybrid **10**. The mobile phase was 60% acetonitrile and 40% H_2O (isocratic). Red line corresponds to UV 210 nm. Blue line corresponds to UV 260 nm.

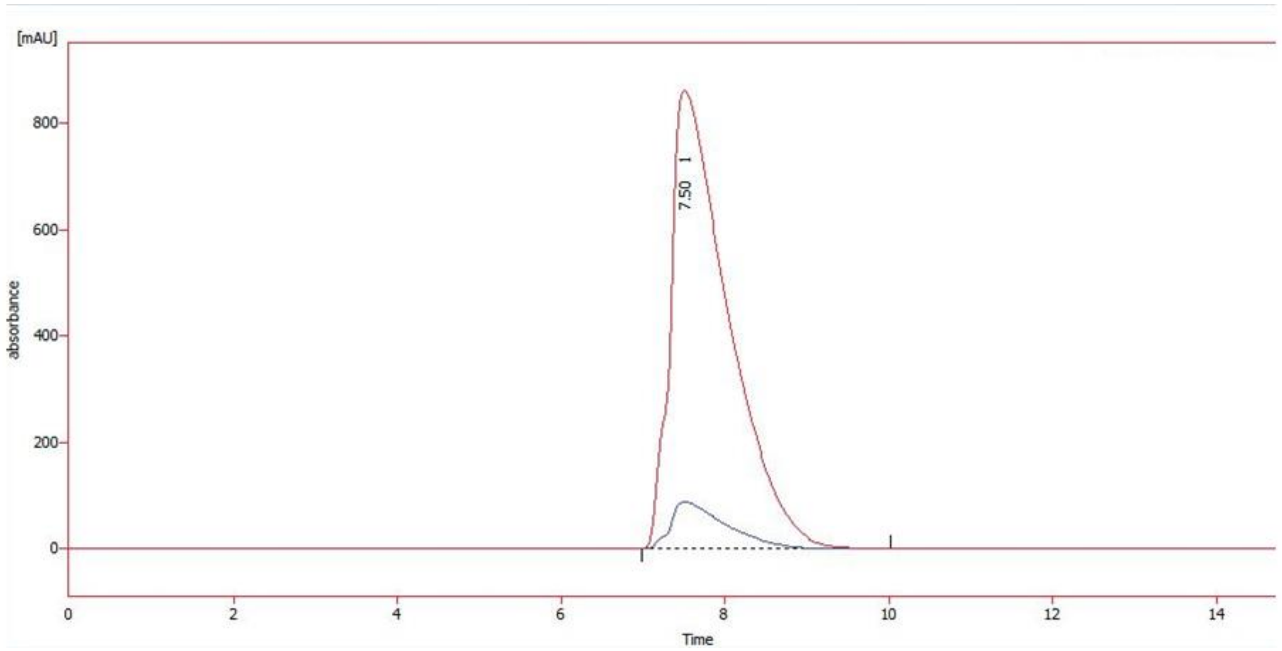

Figure S12. Chiral semi-preparative chromatogram of hybrid **11**. The mobile phase was 60% acetonitrile and 40% H_2O (isocratic). Red line corresponds to UV 210 nm. Blue line corresponds to UV 260 nm.

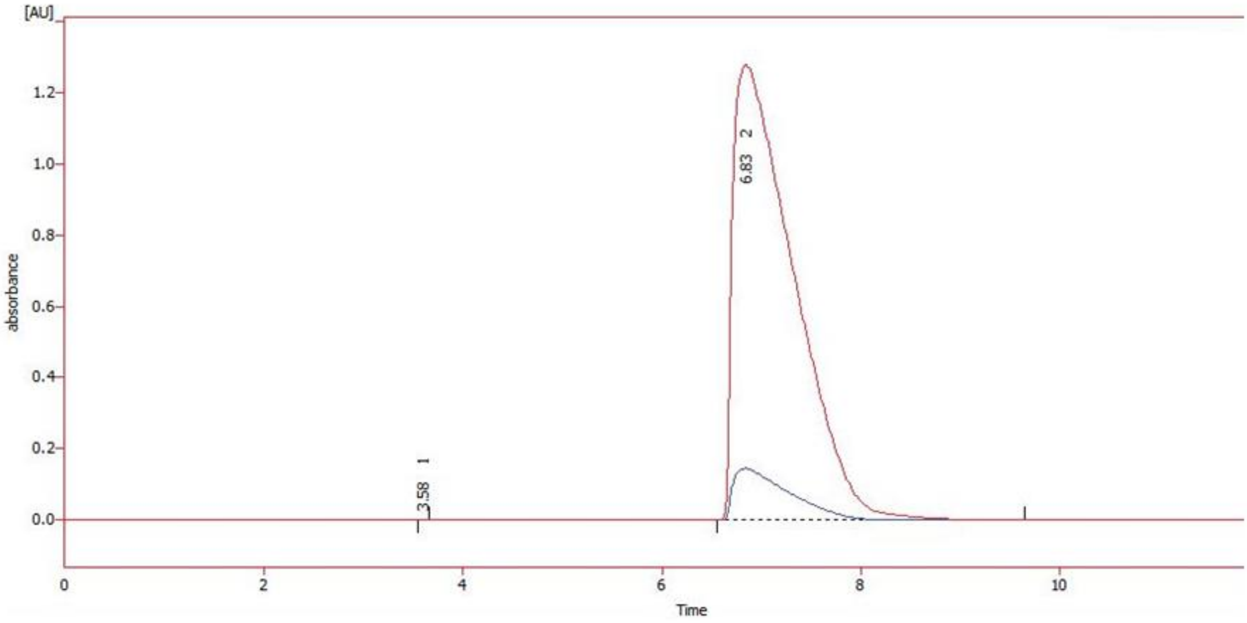

Figure S13. Chiral semi-preparative chromatogram of hybrid **12**. The mobile phase was 60% acetonitrile and 40% H_2O (isocratic). Red line corresponds to UV 210 nm. Blue line corresponds to UV 260 nm.

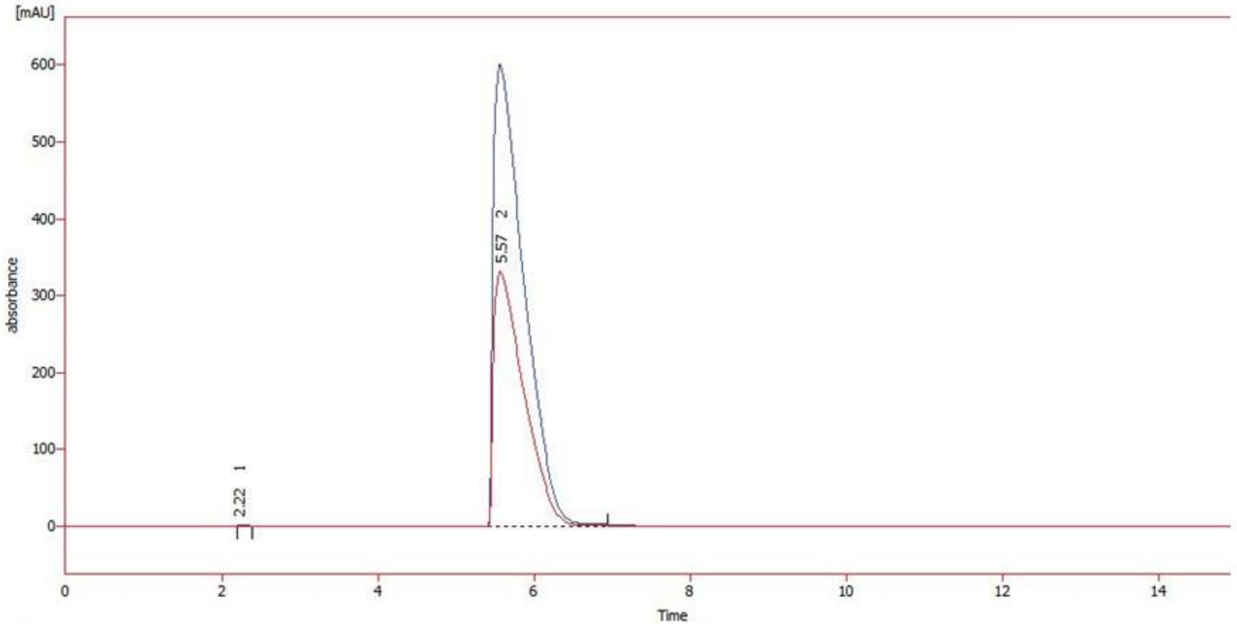

Figure S14. Chiral semi-preparative chromatogram of hybrid **13**. The mobile phase was 60% acetonitrile and 40% H_2O (isocratic). Red line corresponds to UV 210 nm. Blue line corresponds to UV 260 nm.

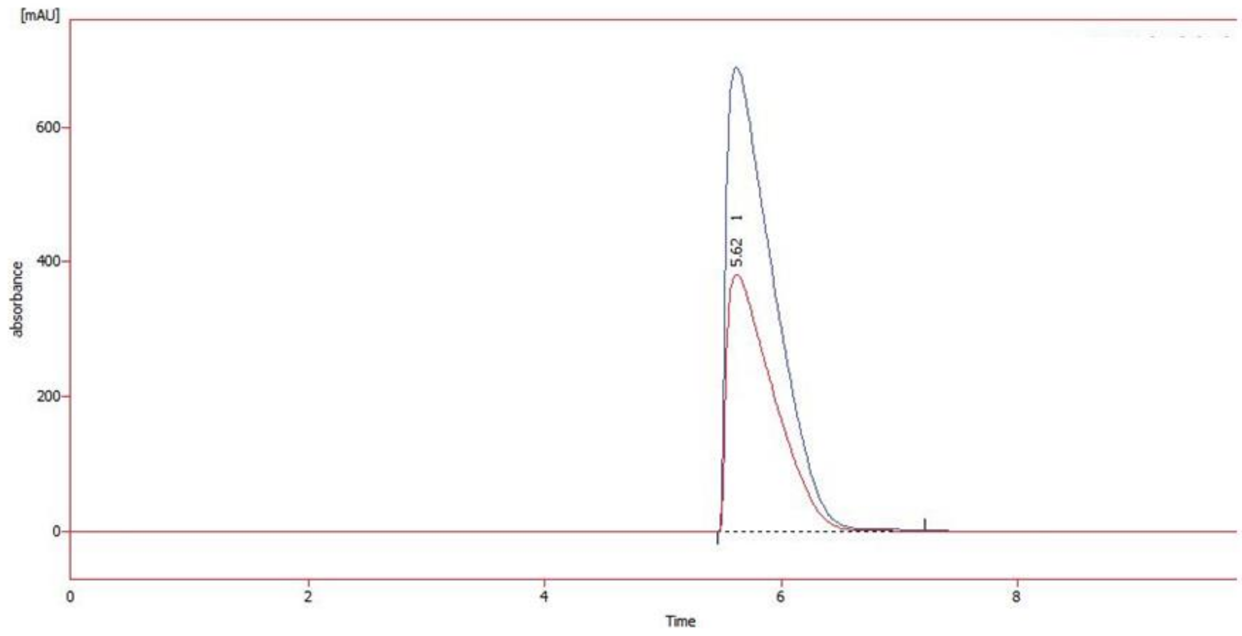

Figure S15. Chiral semi-preparative chromatogram of hybrid **14**. The mobile phase was 60% acetonitrile and 40% H_2O (isocratic). Red line corresponds to UV 210 nm. Blue line corresponds to UV 260 nm.

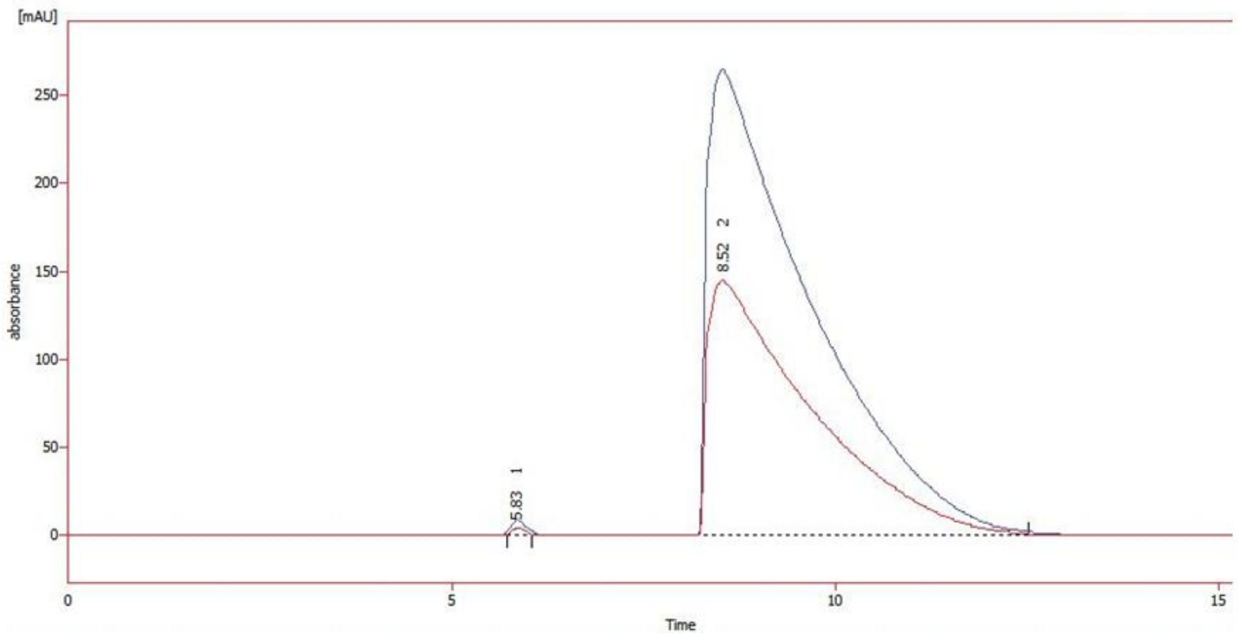

Figure S16. Chiral semi-preparative chromatogram of hybrid **15**. The mobile phase was 60% acetonitrile and 40% H_2O (isocratic). Red line corresponds to UV 210 nm. Blue line corresponds to UV 260 nm.

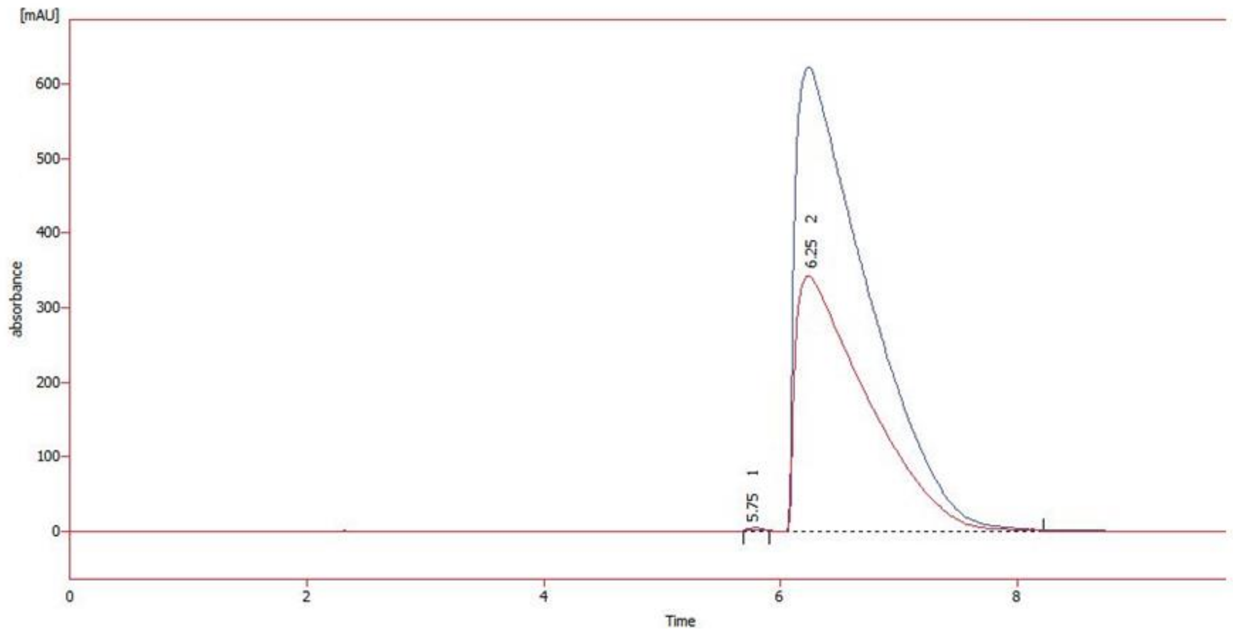

Figure S17. Chiral semi-preparative chromatogram of hybrid **16**. The mobile phase was 60% acetonitrile and 40% H_2O (isocratic). Red line corresponds to UV 210 nm. Blue line corresponds to UV 260 nm.

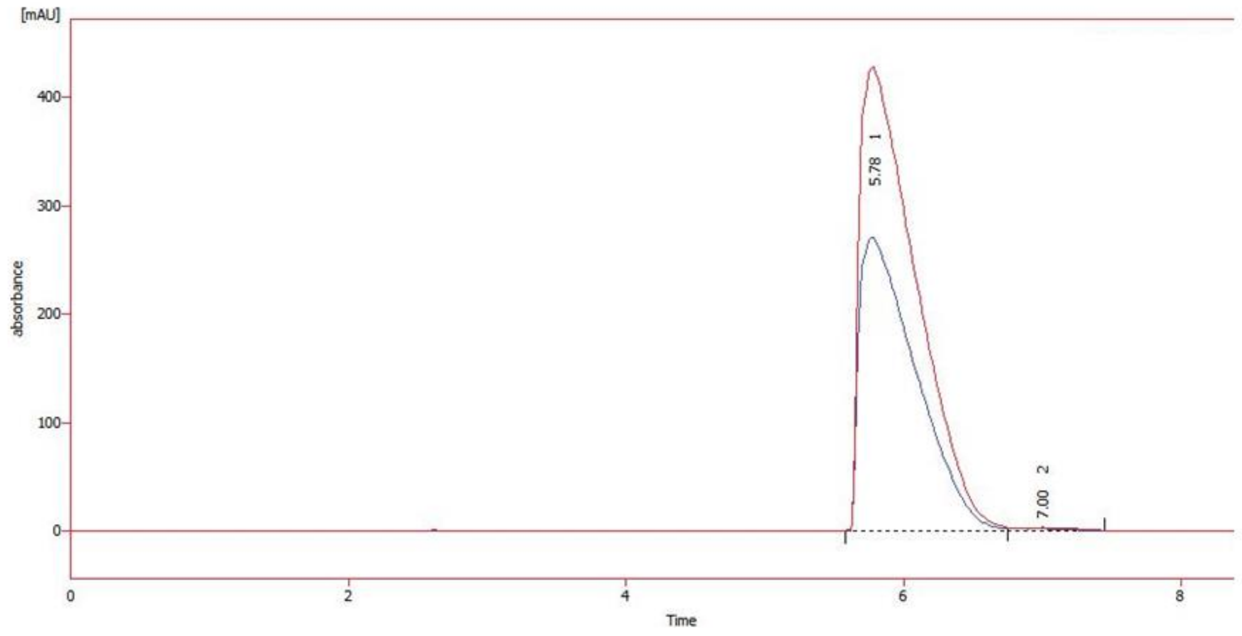

Figure S18. Chiral semi-preparative chromatogram of hybrid **17**. The mobile phase was 60% acetonitrile and 40% H_2O (isocratic). Red line corresponds to UV 210 nm. Blue line corresponds to UV 260 nm.

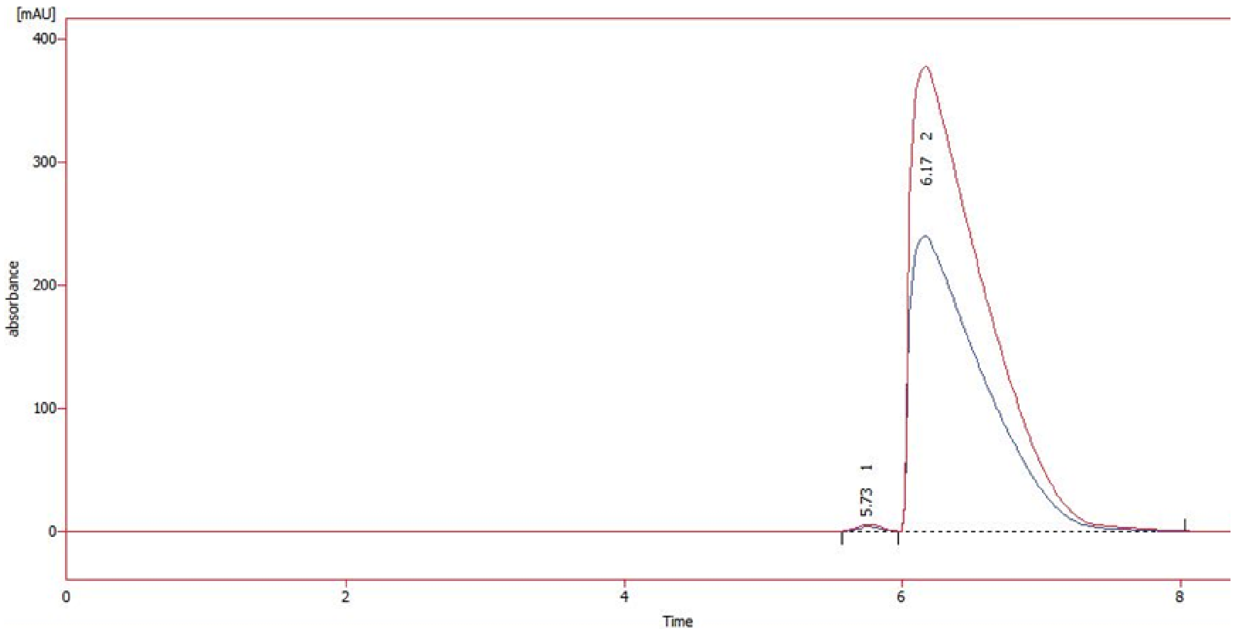

Figure S19. Chiral semi-preparative chromatogram of hybrid **18**. The mobile phase was 60% acetonitrile and 40% H_2O (isocratic). Red line corresponds to UV 210 nm. Blue line corresponds to UV 260 nm.

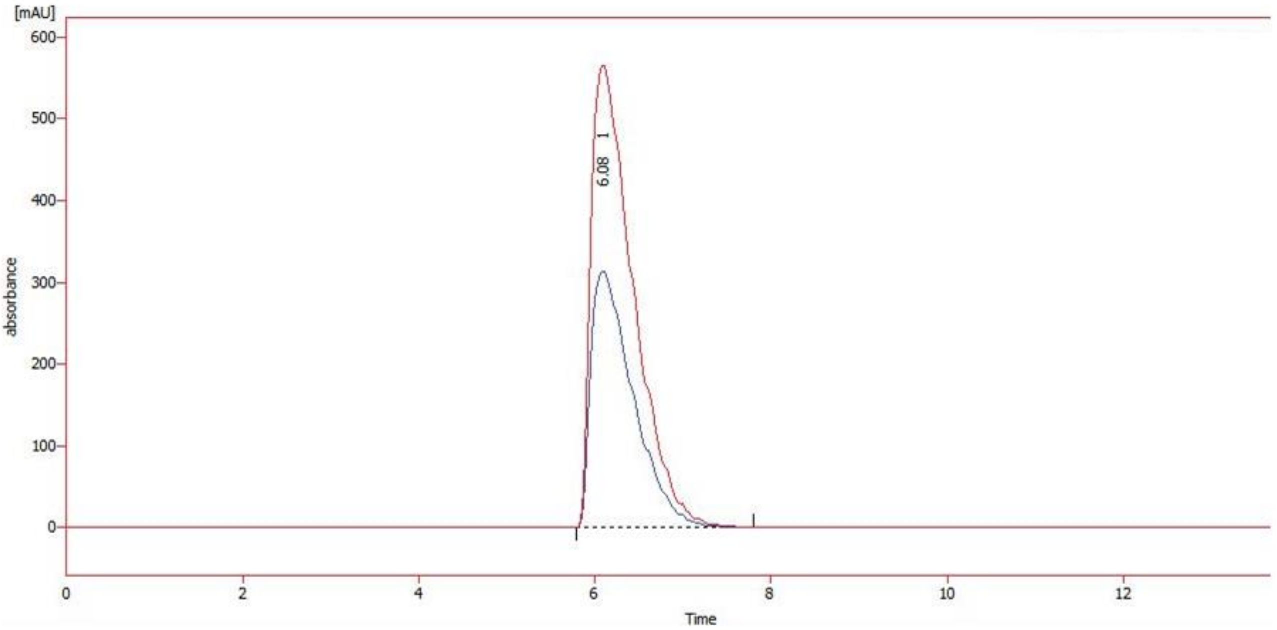

Figure S20. Chiral semi-preparative chromatogram of hybrid **19**. The mobile phase was 60% acetonitrile and 0.1% TFA in 40% H_2O (isocratic). Red line corresponds to UV 210 nm. Blue line corresponds to UV 260 nm.

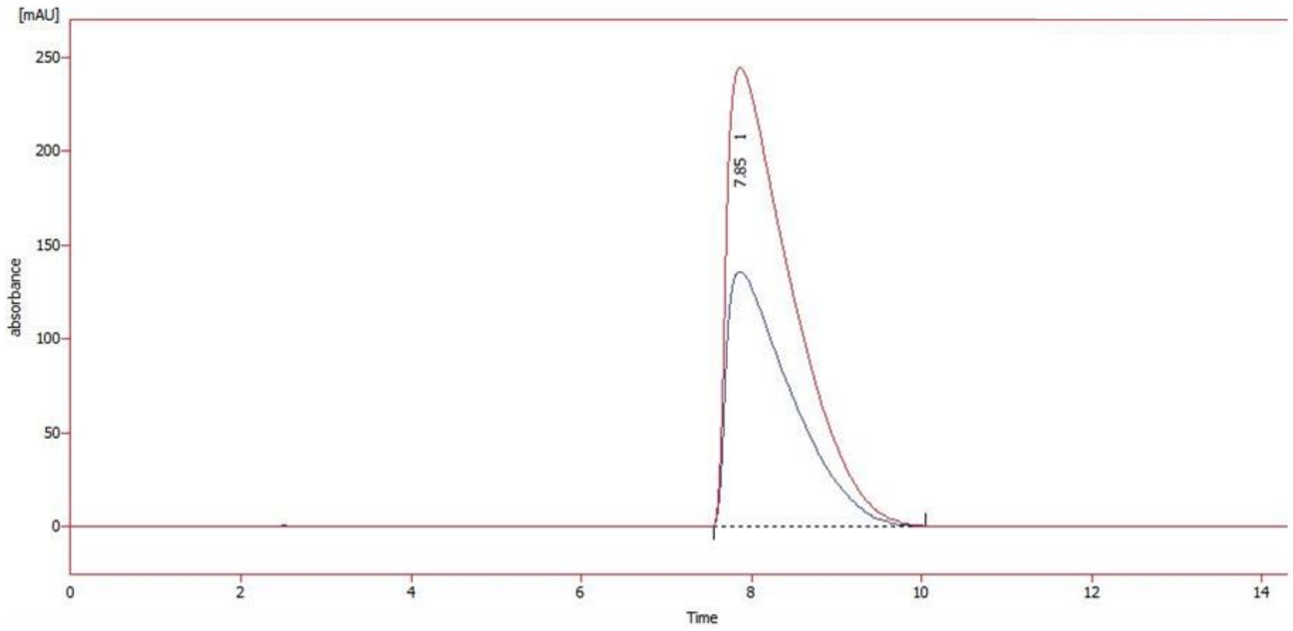

Figure S21. Chiral semi-preparative chromatogram of hybrid **20**. The mobile phase was 60% acetonitrile and 0.1% TFA in 40% H_2O (isocratic). Red line corresponds to UV 210 nm. Blue line corresponds to UV 260 nm.

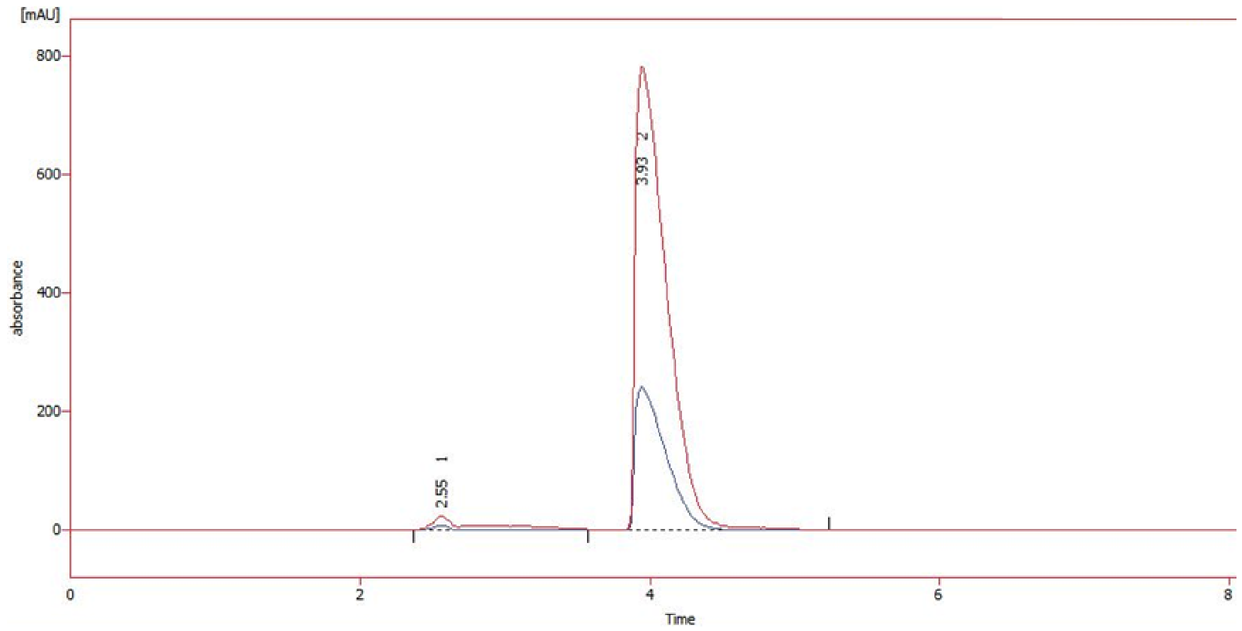

Figure S22. Chiral semi-preparative chromatogram of hybrid **21**. The mobile phase was 60% acetonitrile and 40% H_2O (isocratic). Red line corresponds to UV 210 nm. Blue line corresponds to UV 260 nm.

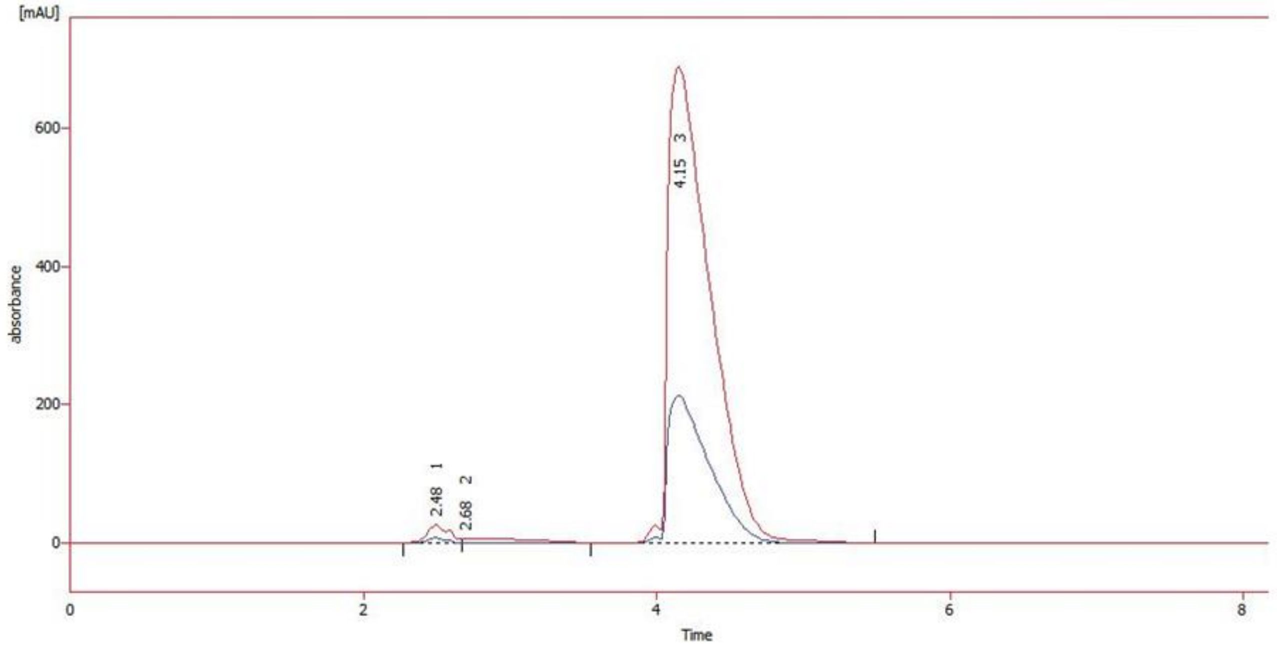

Figure S23. Chiral semi-preparative chromatogram of hybrid **22**. The mobile phase was 60% acetonitrile and 40% H_2O (isocratic). Red line corresponds to UV 210 nm. Blue line corresponds to UV 260 nm.

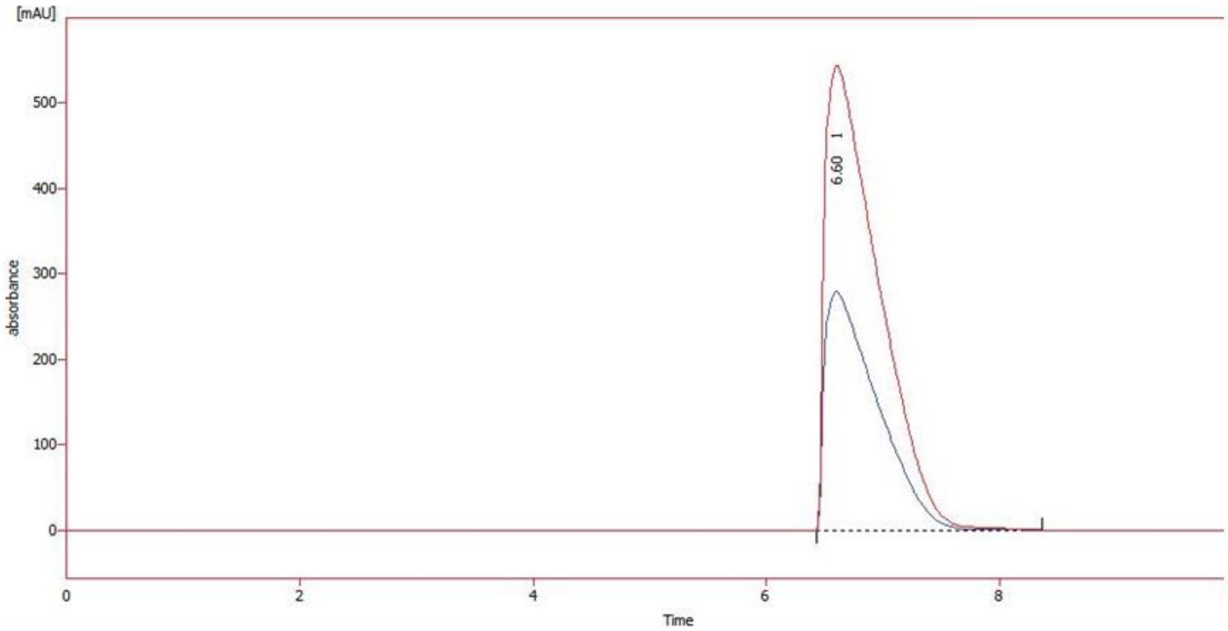

Figure S24. Chiral semi-preparative chromatogram of hybrid **23**. The mobile phase was 60% acetonitrile and 40% H_2O (isocratic). Red line corresponds to UV 210 nm. Blue line corresponds to UV 260 nm.

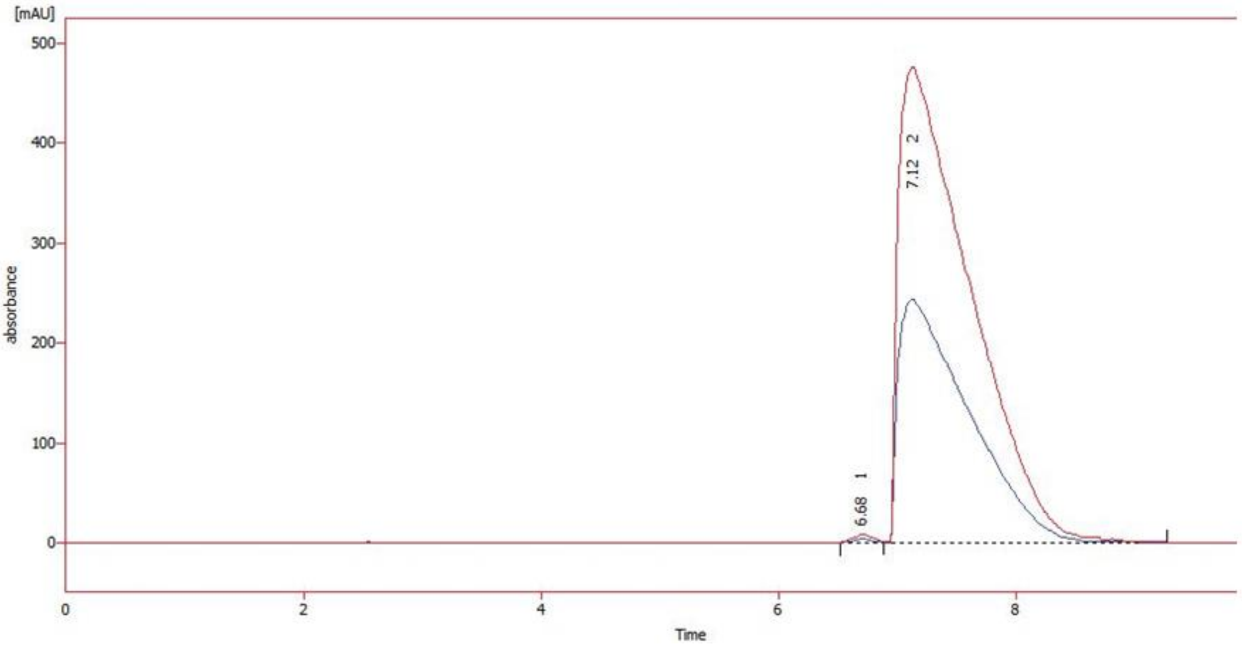

Figure S25. Chiral semi-preparative chromatogram of hybrid **24**. The mobile phase was 60% acetonitrile and 40% H_2O (isocratic). Red line corresponds to UV 210 nm. Blue line corresponds to UV 260 nm.

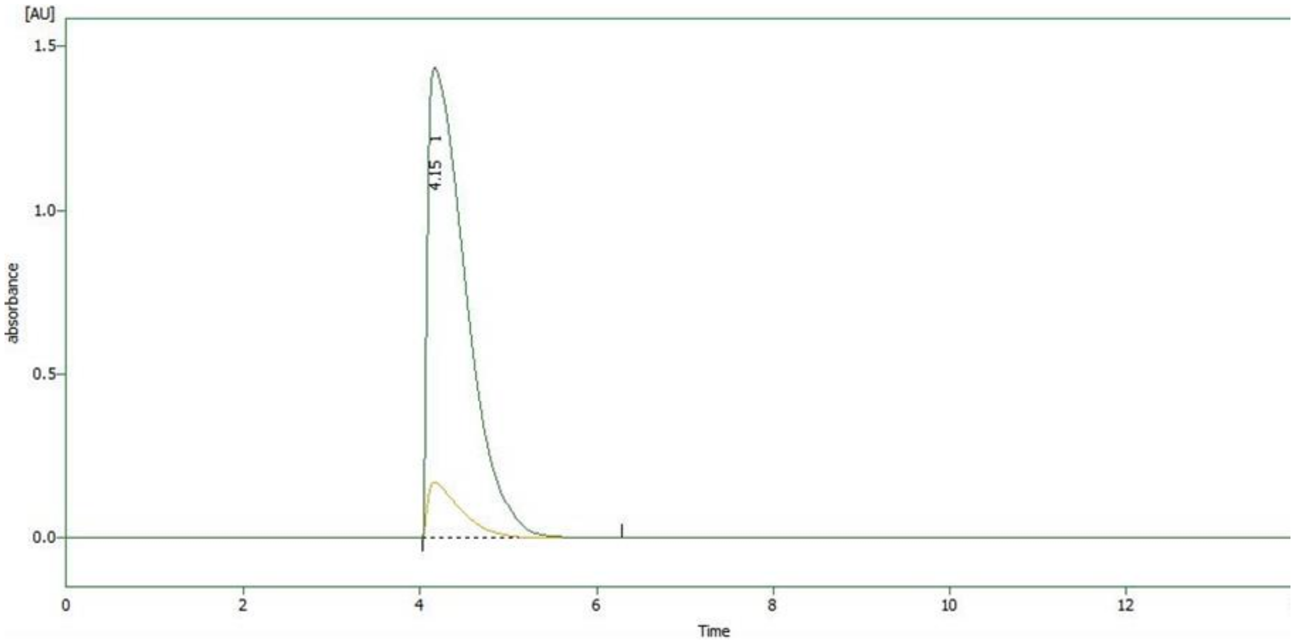

Figure S26. Chiral semi-preparative chromatogram of compound **N-tosyl azole (*S*)**. The mobile phase was 60% acetonitrile and 40% H_2O (isocratic). Green line corresponds to UV 210 nm. Yellow line corresponds to UV 260 nm.

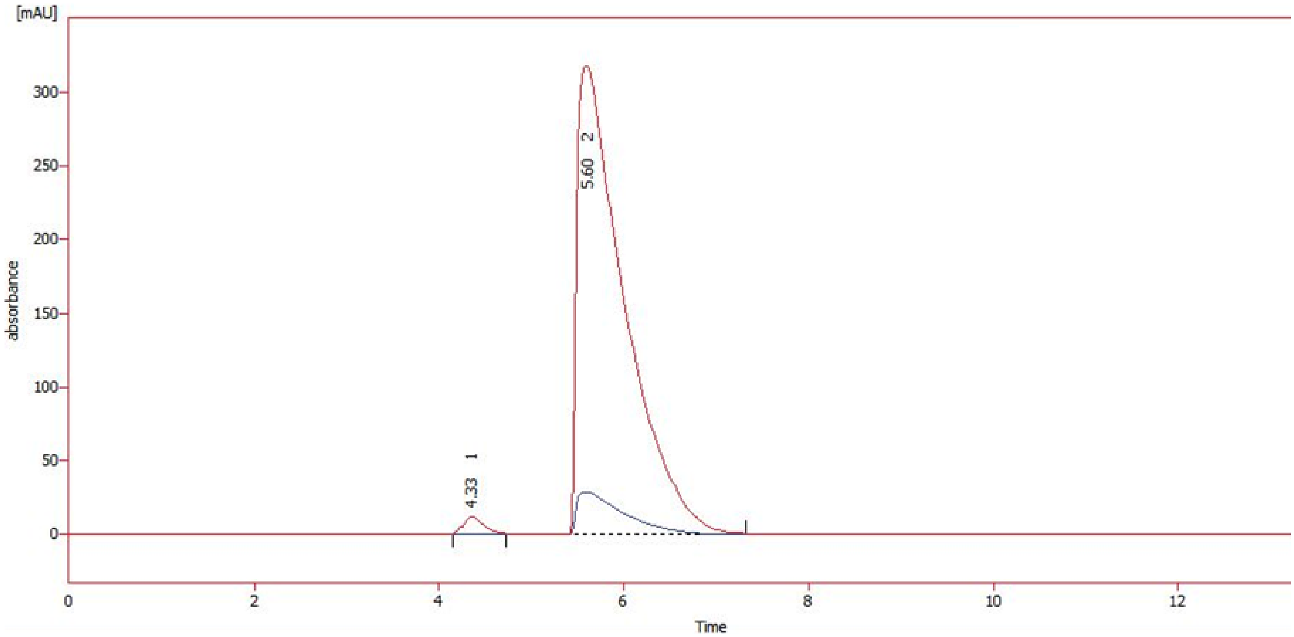

Figure S27. Chiral semi-preparative chromatogram of compound **N-tosyl azole (*R*)**. The mobile phase was 60% acetonitrile and 40% H_2O (isocratic). Red line corresponds to UV 210 nm. Blue line corresponds to UV 260 nm.

4. Compound purity information

Compound	Time (min)	Purity (%)	ee (%)	Compound	Time (min)	Purity (%)	ee (%)
**1**	10.03	>99	>99	**14**	5.62	>99	>99
**2**	8.25	>99	98	**15**	8.52	>99	>99
**3**	7.57	>99	>99	**16**	6.25	>99	>99
**4**	7.10	>99	>99	**17**	5.78	>99	98
**5**	7.30	>99	>99	**18**	6.17	>99	>99
**6**	7.40	>99	>99	**19**	6.08	>99	>99
**7**	8.65	>99	98	**20**	7.85	>99	>99
**8**	7.98	>99	>99	**21**	3.93	96	>99
**9**	6.93	>99	>99	**22**	4.15	96	95
**10**	6.53	>99	>99	**23**	6.60	>99	>99
**11**	7.50	>99	>99	**24**	7.12	>99	>99
**12**	6.83	>99	>99	**N-tosyl azole (*S*)**	4.15	>99	>99
**13**	5.57	>99	>99	**N-tosyl azole (*R*)**	5.60	98	97

Table S1: HPLC conditions for separation: **1-18, 21-24, N-tosyl azole (*S*)** and **N-tosyl azole (*R*)**: Solvent A was 40% H_2O and solvent B was 60% acetonitrile. **19-20**: Solvent A was 0.1% TFA in 40% H_2O and solvent B was 60% acetonitrile. The flow rate was 5 mL/min.

5. Biological information

A. Yeast strains

#	Species	Strain Name	Parental strain	Genotype	Source
1.	*C. albicans*	SC5314	WT		David Perlin[2]
2.	*C. albicans*	ATCC 90028	-		ATCC
3.	*C. albicans*	ATCC 24433	-		ATCC
4.	*C. albicans*	P-87	clinical isolate		Matthew P. Hirakawa[3]
5.	*C. albicans*	SN152	-	*leu2Δ/leu2Δ his1Δ/his1Δ arg4Δ/arg4Δ URA3/ura3Δ::imm434 IRO1/iro1Δ::imm*	Susan Lindquist[4]
6.	*C. albicans*	*BV11/ erg3Δ/Δ erg11Δ/Δ*	*SN152*	*erg3Δ::C.d.HIS1/erg3Δ :C.m.LEU2 erg11Δ:C.d.ARG4/erg 11Δ/C.d.ARG4*	Susan Lindquist[4]
7.	*C. glabrata*	T-50 (BG2)	clinical isolate		Brendan Cormack[5]
8.	*C. glabrata*	ATCC 66032	-		ATCC
9.	*C. glabrata*	ATCC 2001	-		Cecile Fairhead[6]
10.	*C. glabrata*	T-191 (11-331)	clinical isolate		Ronen Ben Ami[7]
11.	*C. glabrata*	T-195 (11-064)	clinical isolate		Ronen Ben Ami[7]
12.	*C. parapsilosis*	ATCC 22019			ATCC
13.	*C. guilliermondii*	T-47 (B-3163)	WT		Mike McEachern[8]
14.	*C. tropicalis*	660	WT		P. T. Magee[9]
15.	*C. dubliniensis*	T-99 (Wu284)			Gary Moran[10]
16.	*C. auris*	ATCC MYA5001			ATCC
17.	*C. auris*	ATCC MYA5002			ATCC

Table S2. Yeast strain information.

B. Minimal inhibitory concentrations (MICs) tables

Compound	*C. auris* 5001	*C. auris* 5002
**FLC**	8	>64
**VOR**	0.03	1
**1**	0.06	1
**2**	0.06	1
**5**	0.06	4
**10**	0.5	16
**17**	0.25	4
**19**	0.12	2
**21**	0.25	8
**23**	2	64

Table S3. MIC values of selected hybrids against *C. auris*. Cells were grown in YPAD medium at 37 °C for 24 h. Values are given in μg/mL.

Compound	*C. albicans* SC5314	*C. albicans* 90028	*C. albicans* 24433	*C. albicans* P-87	*C. albicans* SN152	*C. albicans erg3Δ/Δ erg11Δ/Δ*
**FLC**	0.5	0.5	1	1	0.5	>64
**VOR**	0.007	0.007	0.015	0.007	0.007	>64
**1**	0.003	0.003	0.007	0.003	0.003	64
**2**	0.007	0.007	0.015	0.007	0.007	64
**3**	0.03	0.06	0.12	0.03	0.06	64
**4**	1	1	2	0.5	1	>64
**5**	0.007	0.015	0.03	0.015	0.007	64
**6**	0.03	0.12	0.12	0.03	0.06	>64
**7**	4	8	16	4	8	64
**8**	32	64	>64	64	64	>64
**9**	0.25	0.25	0.5	0.25	0.012	64
**10**	0.12	0.12	0.25	0.25	0.012	>64
**11**	0.5	1	1	1	0.5	>64
**12**	2	4	4	4	2	>64
**13**	2	2	4	4	2	>64
**14**	1	1	1	1	0.5	>64
**15**	>64	>64	>64	>64	>64	>64
**16**	>64	>64	>64	>64	>64	>64
**17**	0.12	0.12	0.12	0.25	0.06	16
**18**	16	16	8	16	8	64
**19**	0.06	0.12	0.06	0.03	0.03	64
**20**	8	16	16	8	8	32
**21**	0.12	0.25	0.12	0.12	0.12	>64
**22**	8	8	16	16	8	>64
**23**	1	1	1	1	1	>64
**24**	>64	>64	>64	>64	>64	>64

Table S4. MIC values of hybrids **1-24** against *C. albicans*. Cells were grown in YPAD medium at 30 °C for 24 h. Values are given in μg/mL.

Compound	*C. glabrata* 50	*C. glabrata* 66032	*C. glabrata* 2001	*C. glabrata* 191	*C. glabrata* 195
**FLC**	16	32	16	16	16
**VOR**	1	1	0.5	0.5	1
**1**	1	2	0.5	0.5	1
**2**	1	0.5	0.25	2	2
**3**	16	8	8	16	16
**4**	>64	>64	>64	>64	>64
**5**	2	1	0.5	2	2
**6**	>64	>64	>64	>64	>64
**7**	>64	>64	>64	>64	>64
**8**	>64	>64	>64	>64	>64
**9**	32	16	8	16	32
**10**	>64	8	4	32	32
**11**	>64	>64	32	64	64
**12**	>64	>64	>64	>64	>64
**13**	64	32	16	64	64
**14**	32	16	8	16	64
**15**	>64	>64	>64	>64	>64
**16**	>64	>64	>64	>64	>64
**17**	16	16	16	16	16
**18**	64	64	32	64	64
**19**	8	4	2	4	8
**20**	>64	>64	>64	>64	>64
**21**	1	4	2	2	2
**22**	>64	>64	>64	>64	>64
**23**	8	16	8	16	32
**24**	>64	>64	>64	>64	>64

Table S5. MIC values of hybrids **1-24** against *C. glabrata*. Cells were grown in YPAD medium at 30 °C for 24 h. Values are given in μg/mL.

Compound	*C. parapsilosis* 22019	*C. guilliermondii* T-47	*C. tropicalis* 660	*C. dubliniensis* T-99
**FLC**	1	4	1	0.25
**VOR**	0.015	0.06	0.12	0.03
**1**	0.007	0.06	0.03	0.06
**2**	0.015	0.06	0.5	0.12
**3**	0.5	1	1	1
**4**	8	16	>64	0.03
**5**	0.5	0.5	0.06	0.5
**6**	2	2	8	2
**7**	8	32	64	0.5
**8**	64	64	>64	2
**9**	1	2	2	4
**10**	1	1	4	2
**11**	4	8	8	8
**12**	4	32	>64	32
**13**	8	32	16	64
**14**	8	16	16	4
**15**	>64	>64	>64	16
**16**	>64	>64	>64	16
**17**	0.5	2	2	2
**18**	8	>64	>64	16
**19**	0.06	2	0.12	2
**20**	8	>64	>64	0.5
**21**	1	1	0.12	2
**22**	8	16	32	>64
**23**	0.5	8	>64	4
**24**	>64	>64	>64	2

Table S6. MIC values of hybrids **1-24** against *C. parapsilosis, C. guilliermondii, C. tropicalis*, and *C. dubliniensis*. Cells were grown in YPAD medium at 30 °C for 24 h. Values are given in μg/mL.

6. References

(1) Pore, V. S.; Agalave, S. G.; Singh, P.; Shukla, P. K.; Kumar, V.; Siddiqi, M. I.; Design and Synthesis of New Fluconazole Analogues. *Org. Biomol. Chem* **2015**, *13* (23), 6551–6561. https://doi.org/10.1039/c5ob00590f.

(2) Jones, T.; Federspiel, N. A.; Chibana, H.; Dungan, J.; Kalman, S.; Magee, B. B.; Newport, G.; Thorstenson, Y. R.; Agabian, N.; Magee, P. T.; Davis, R. W.; Scherer, S. The Diploid Genome Sequence of Candida Albicans. *Proc. Natl. Acad. Sci. U. S. A.* **2004**, *101* (19), 7329–7334. https://doi.org/10.1073/pnas.0401648101.

(3) Hirakawa, M. P.; Martinez, D. A.; Sakthikumar, S.; Anderson, M. Z.; Berlin, A.; Gujja, S.; Zeng, Q.; Zisson, E.; Wang, J. M.; Greenberg, J. M.; Berman, J.; Bennett, R. J.; Cuomo, C. A. Genetic and Phenotypic Intra-Species Variation in Candida Albicans. *Genome Res.* **2015**, *25* (3), 413–425. https://doi.org/10.1101/gr.174623.114.

(4) Vincent, B. M.; Lancaster, A. K.; Scherz-Shouval, R.; Whitesell, L.; Lindquist, S. Fitness Trade-Offs Restrict the Evolution of Resistance to Amphotericin B. *PLoS Biol.* **2013**, *11* (10), e1001692. https://doi.org/10.1371/journal.pbio.1001692.

(5) Cormack, B. P.; Falkow, S. Efficient Homologous and Illegitimate Recombination in the Opportunistic Yeast Pathogen Candida Glabrata. *Genetics* **1999**, *151* (3), 979–987. https://doi.org/10.1093/genetics/151.3.979.

(6) Muller, H.; Hennequin, C.; Gallaud, J.; Dujon, B.; Fairhead, C. The Asexual Yeast Candida Glabrata Maintains Distinct a and α Haploid Mating Types. *Eukaryot. Cell* **2008**, *7* (5), 848–858. https://doi.org/10.1128/EC.00456-07.

(7) Ben-Ami, R.; Zimmerman, O.; Finn, T.; Amit, S.; Novikov, A.; Wertheimer, N.; Lurie-Weinberger, M.; Berman, J. Heteroresistance to Fluconazole Is a Continuously Distributed Phenotype among Candida Glabrata Clinical Strains Associated with in

Vivo Persistence. *MBio.* **2016**, *7* (4). https://doi.org/10.1128/mBio.00655-16.

(8) Mceachern, M. J.; Blackburn, E. H. A Conserved Sequence Motif within the Exceptionally Diverse Telomeric Sequences of Budding Yeasts. *Proc. Natl. Acad. Sci. U. S. A.* **1994**, *91* (8), 3453–3457. https://doi.org/10.1073/pnas.91.8.3453.

(9) Beckerman, J.; Chibana, H.; Turner, J.; Magee, P. T. Single-Copy IMH3 Allele Is Sufficient to Confer Resistance to Mycophenolic Acid in Candida Albicans and to Mediate Transformation of Clinical Candida Species. *Infect. Immun.* **2001**, *69* (1), 108–114. https://doi.org/10.1128/IAI.69.1.108-114.2001.

(10) Moran, G. P.; MacCallum, D. M.; Spiering, M. J.; Coleman, D. C.; Sullivan, D. J. Differential Regulation of the Transcriptional Repressor NRG1 Accounts for Altered Host-Cell Interactions in Candida Albicans and Candida Dubliniensis. *Mol. Microbiol.* **2007**, *66* (4), 915–929. https://doi.org/10.1111/j.1365-2958.2007.05965.x.

7. NMR spectra

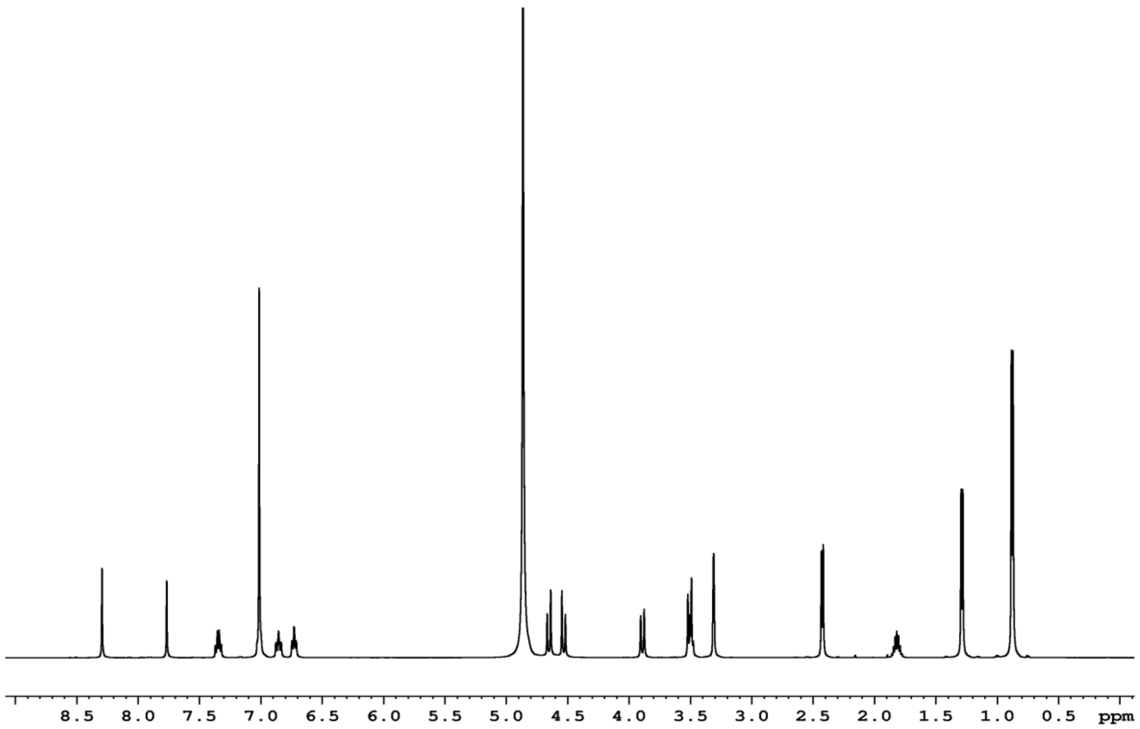

Figure S28. 500 MHz ^{1}H-NMR spectrum of hybrid **1** in CD_3OD at 298 K.

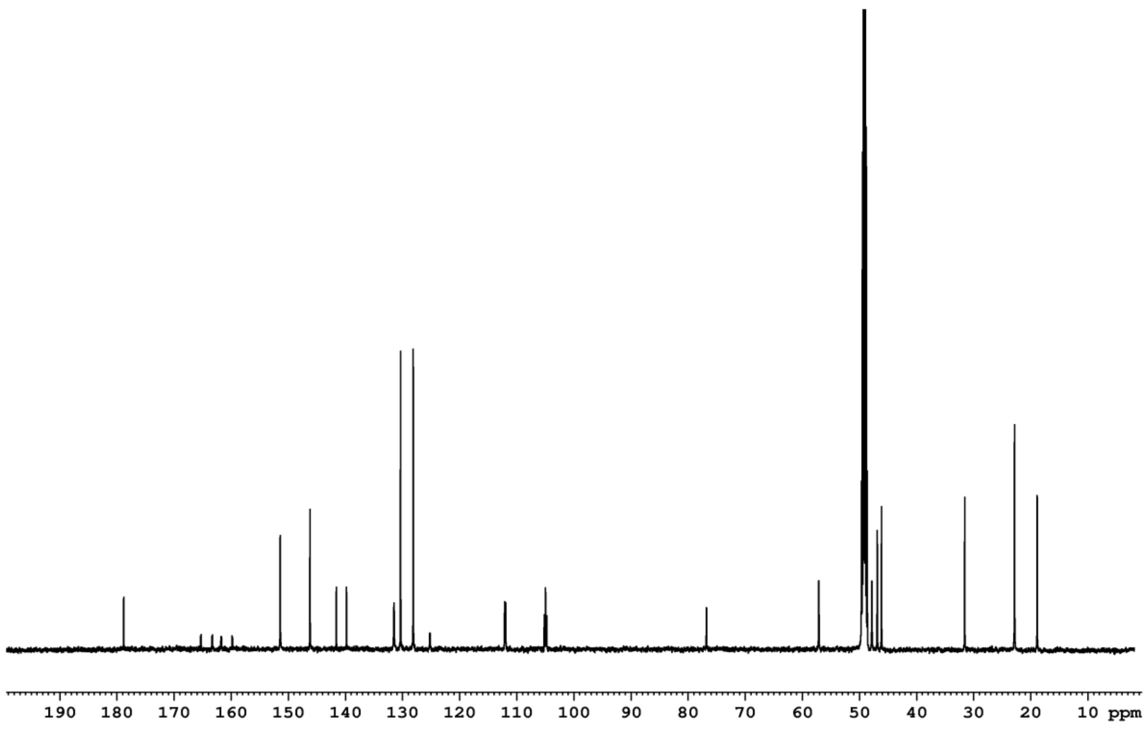

Figure S29. 125 MHz ^{13}C-NMR spectrum of hybrid **1** in CD_3OD at 298 K.

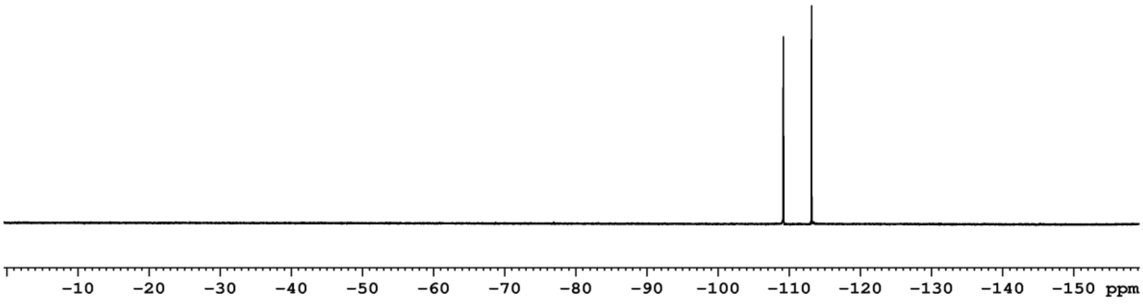

Figure S30. 470 MHz ^{19}F-NMR spectrum of hybrid **1** in CD_3OD at 298 K.

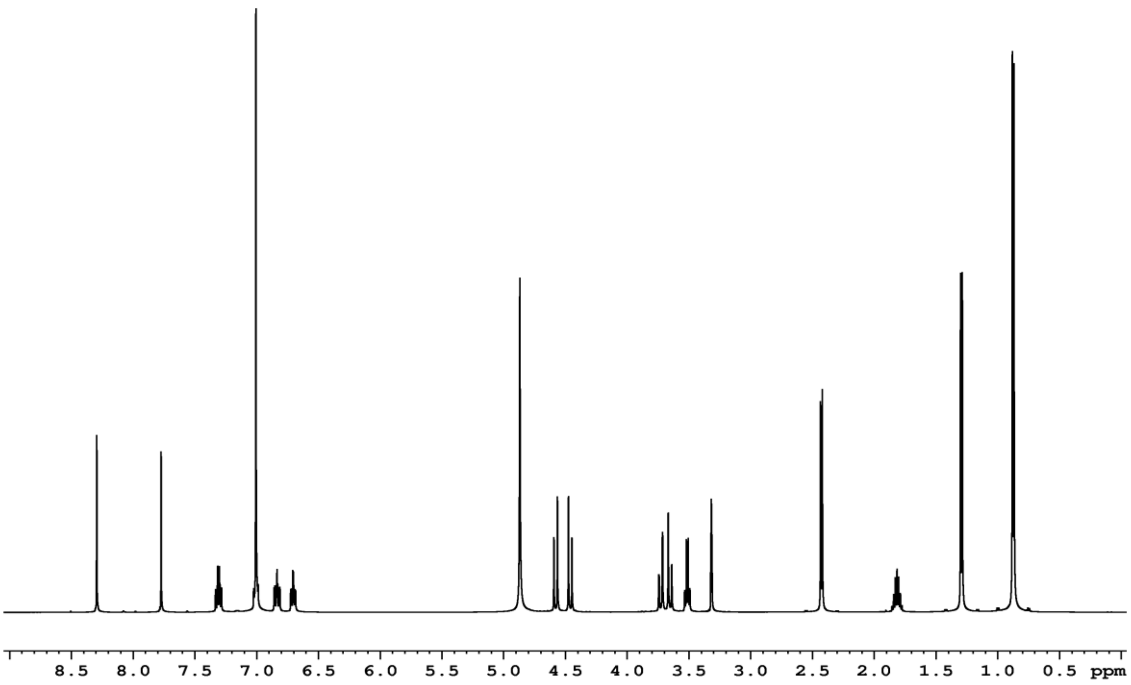

Figure S31. 500 MHz ^{1}H-NMR spectrum of hybrid **2** in CD_3OD at 298 K.

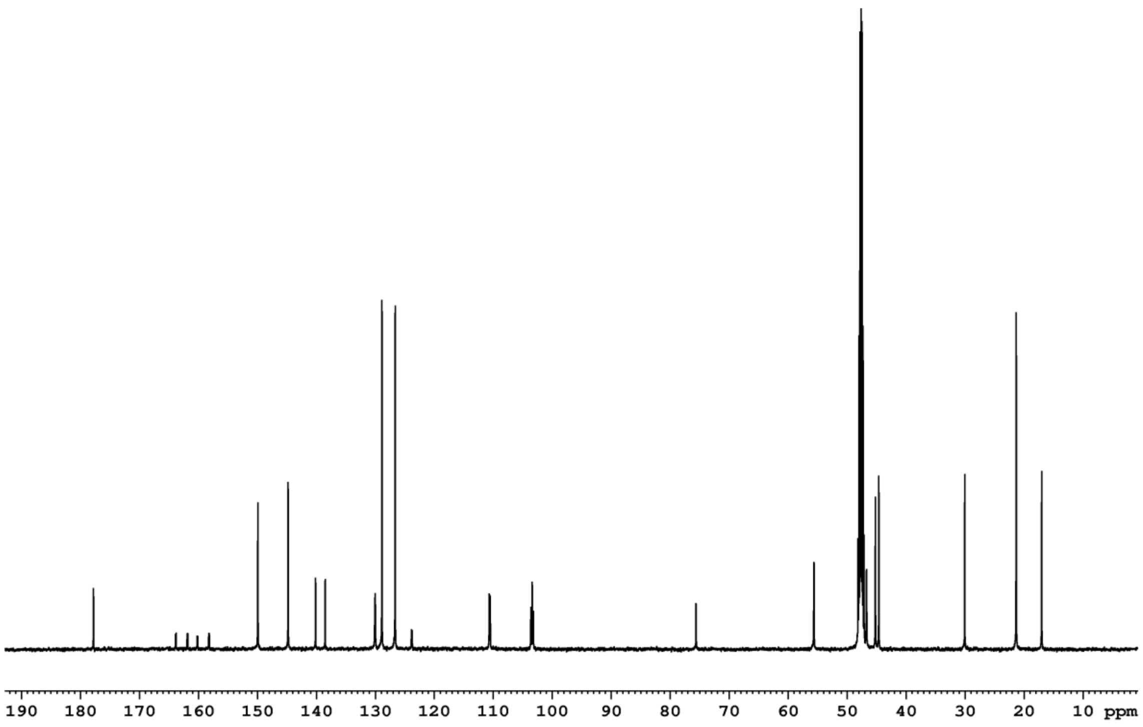

Figure S32. 125 MHz ^{13}C-NMR spectrum of hybrid **2** in CD_3OD at 298 K.

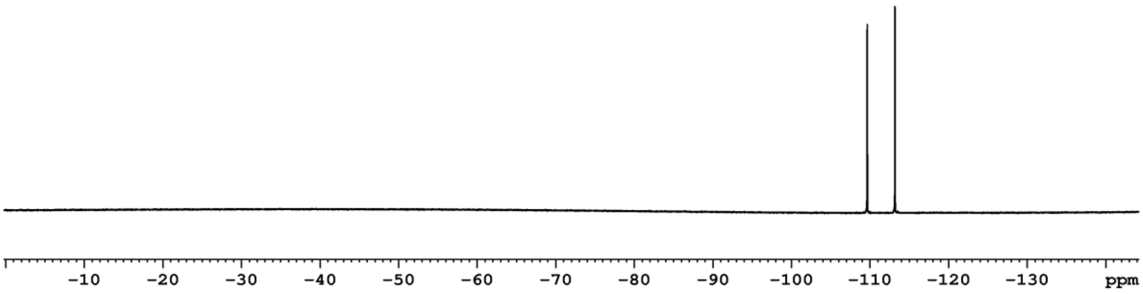

Figure S33. 470 MHz ^{19}F-NMR spectrum of hybrid **2** in CD_3OD at 298 K.

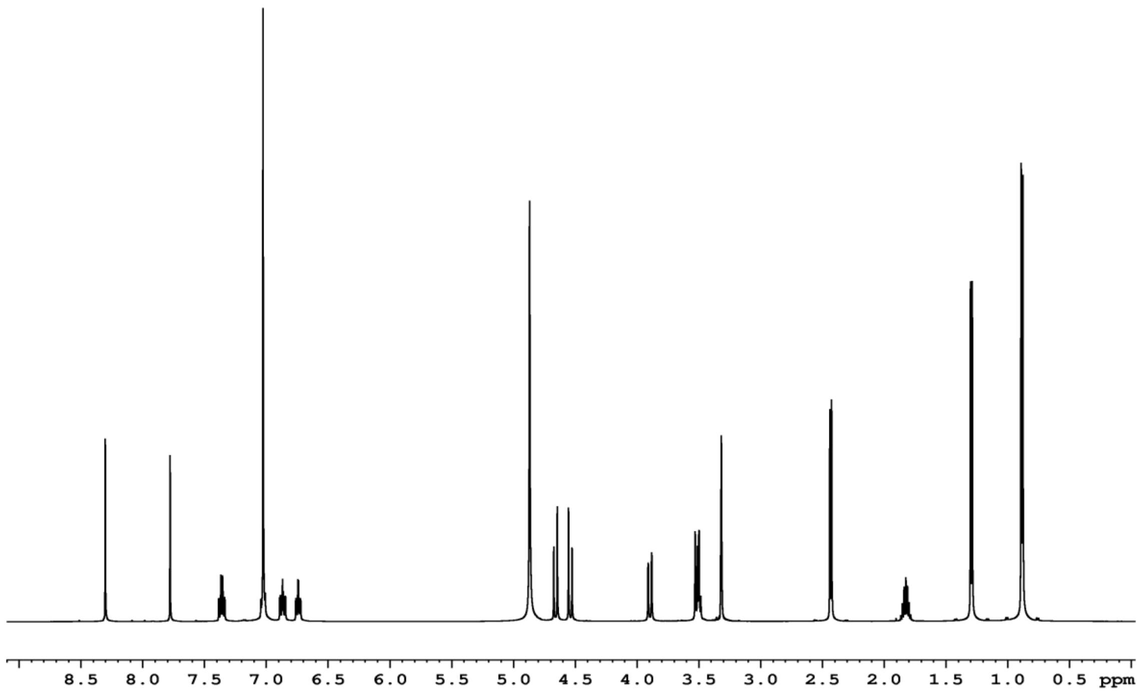

Figure S34. 500 MHz ^{1}H-NMR spectrum of hybrid **3** in CD_3OD at 298 K.

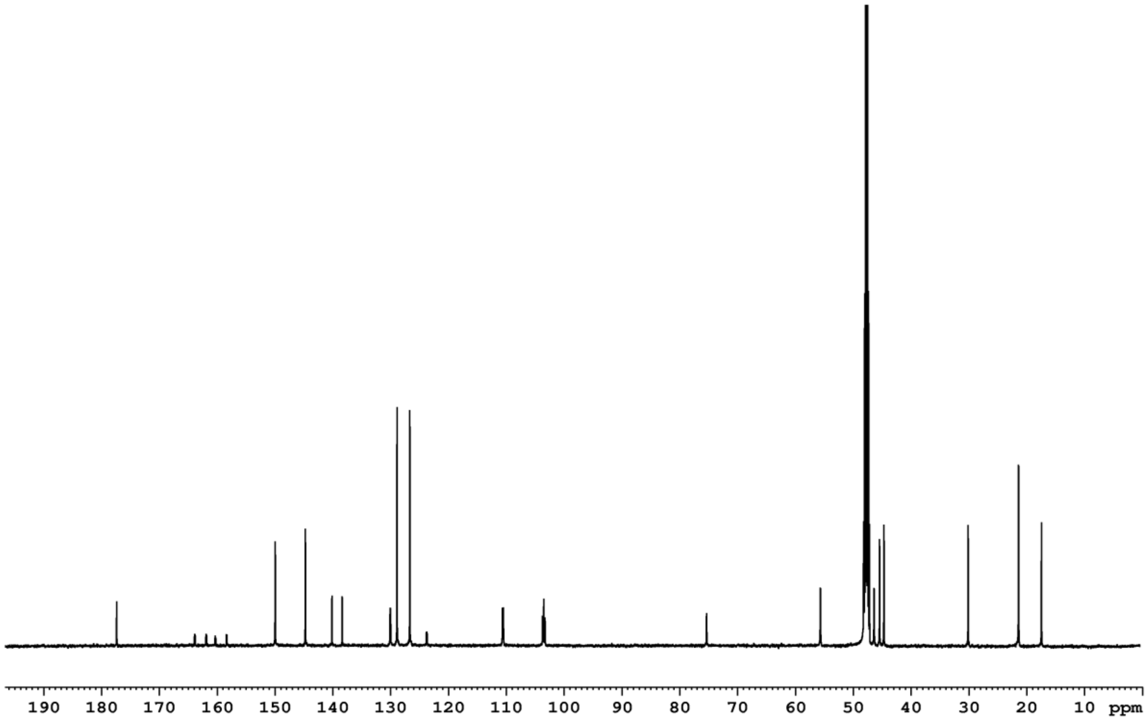

Figure S35. 125 MHz ^{13}C-NMR spectrum of hybrid **3** in CD_3OD at 298 K.

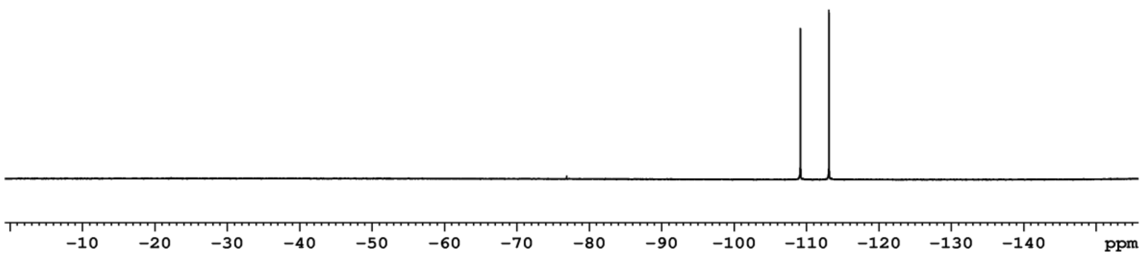

Figure S36. 470 MHz ^{19}F-NMR spectrum of hybrid **3** in CD_3OD at 298 K.

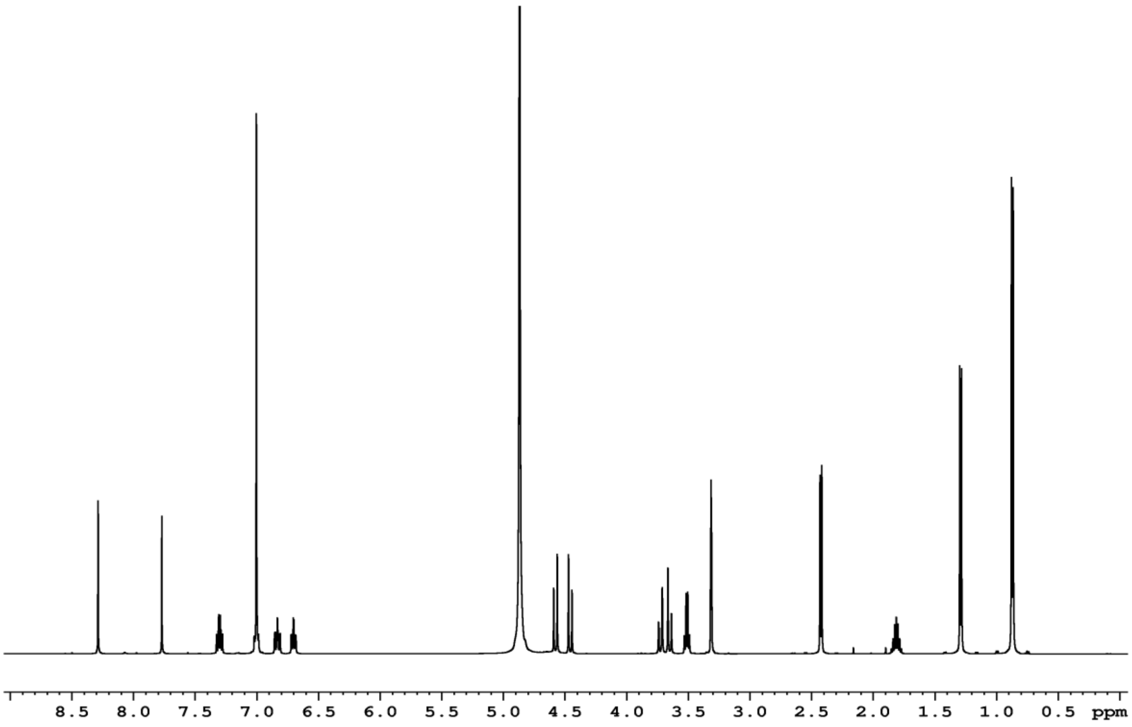

Figure S37. 500 MHz ^{1}H-NMR spectrum of hybrid **4** in CD_3OD at 298 K.

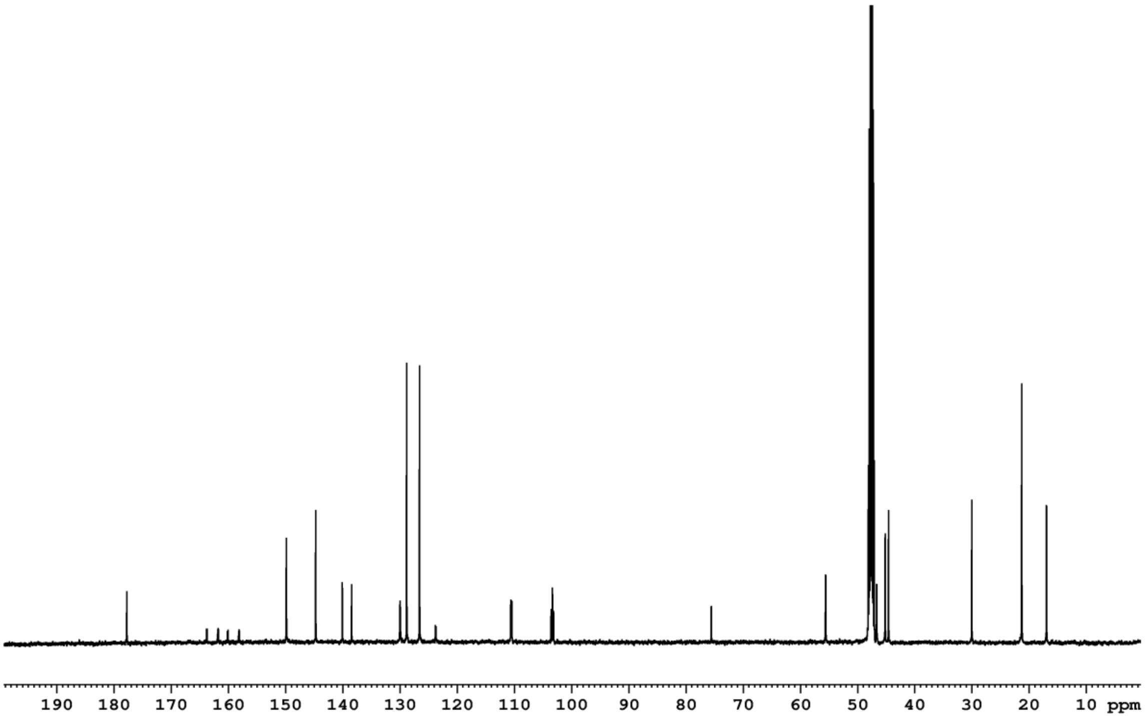

Figure S38. 125 MHz ^{13}C-NMR spectrum of hybrid **4** in CD_3OD at 298 K.

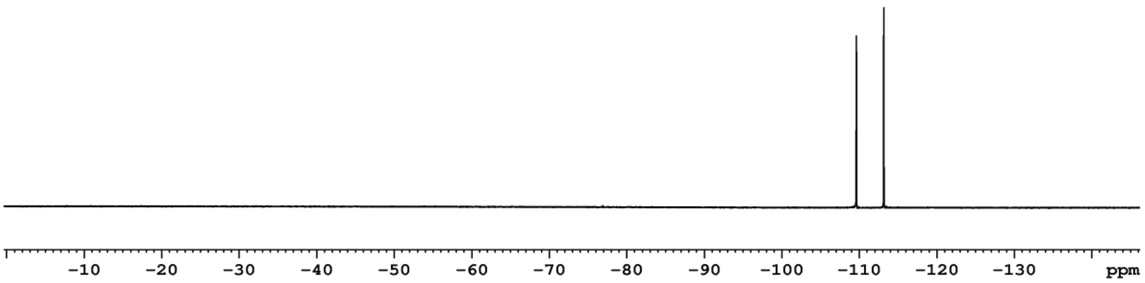

Figure S39. 470 MHz ^{19}F-NMR spectrum of hybrid **4** in CD_3OD at 298 K.

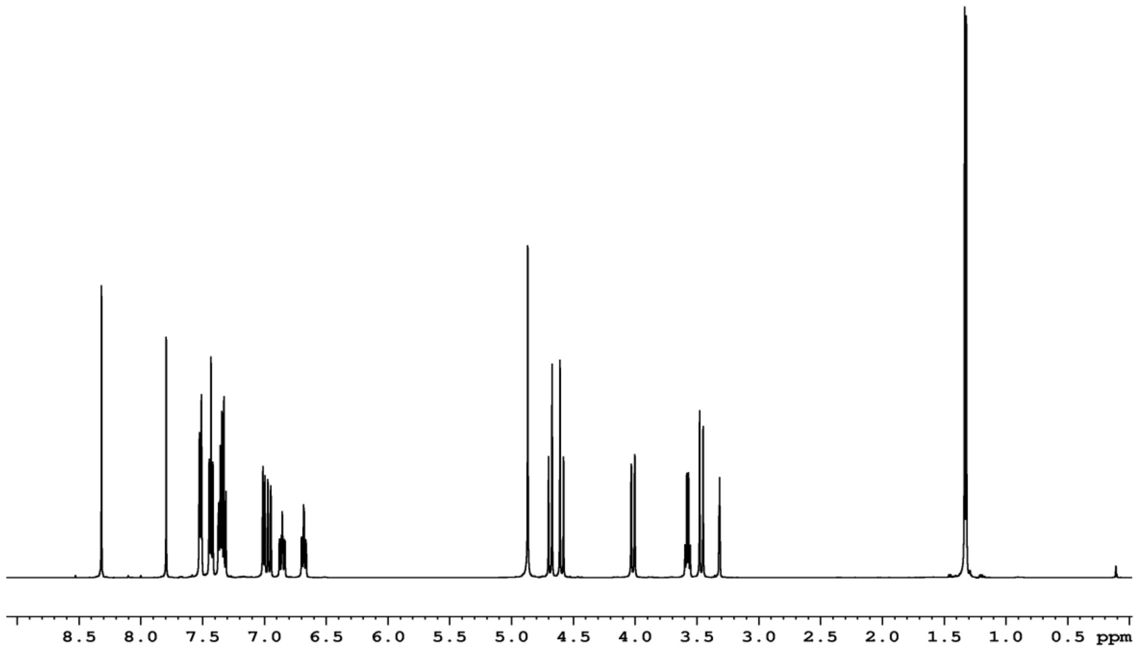

Figure S40. 500 MHz ^{1}H-NMR spectrum of hybrid **5** in CD_3OD at 298 K.

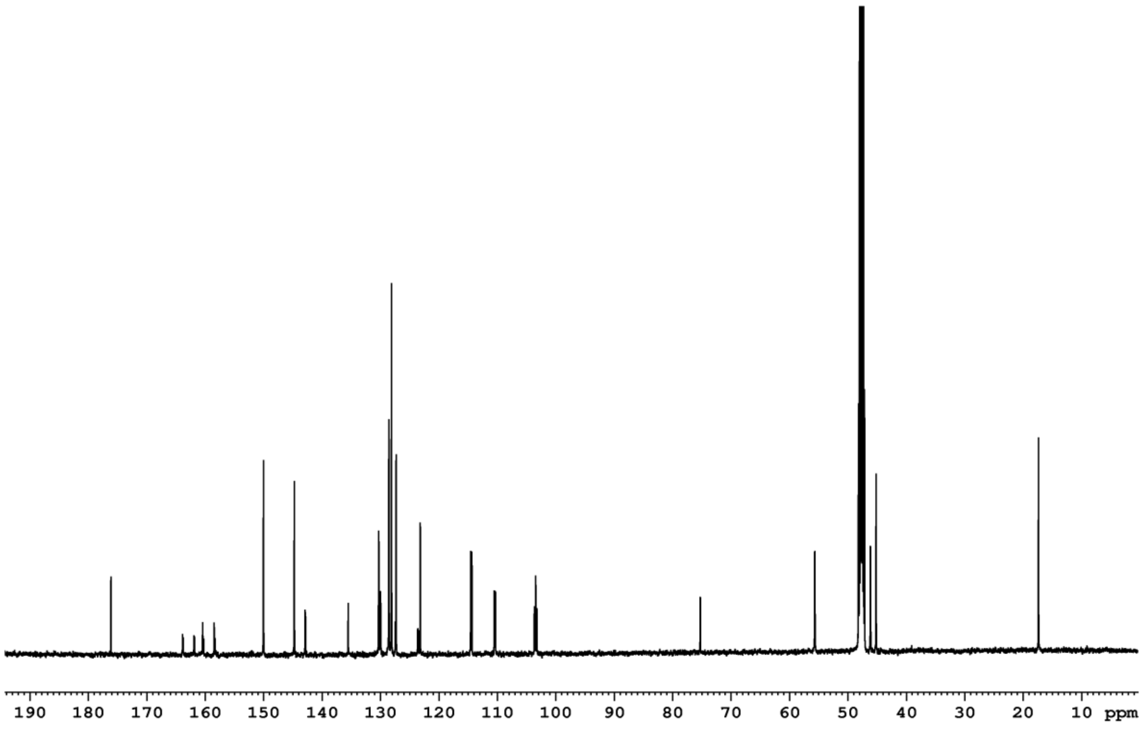

Figure S41. 125 MHz ^{13}C-NMR spectrum of hybrid **5** in CD_3OD at 298 K.

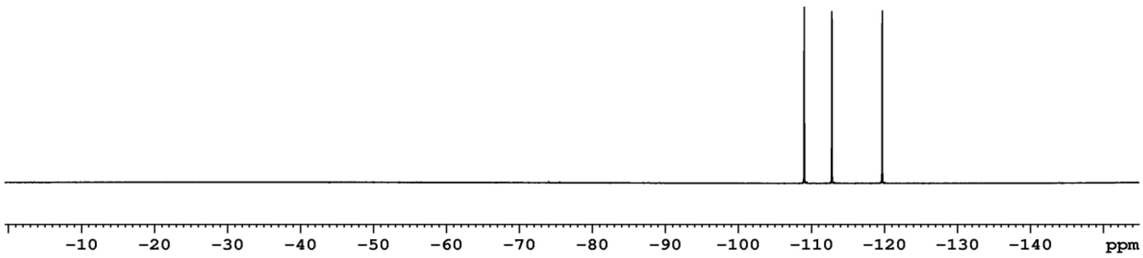

Figure S42. 470 MHz ^{19}F-NMR spectrum of hybrid **5** in CD_3OD at 298 K.

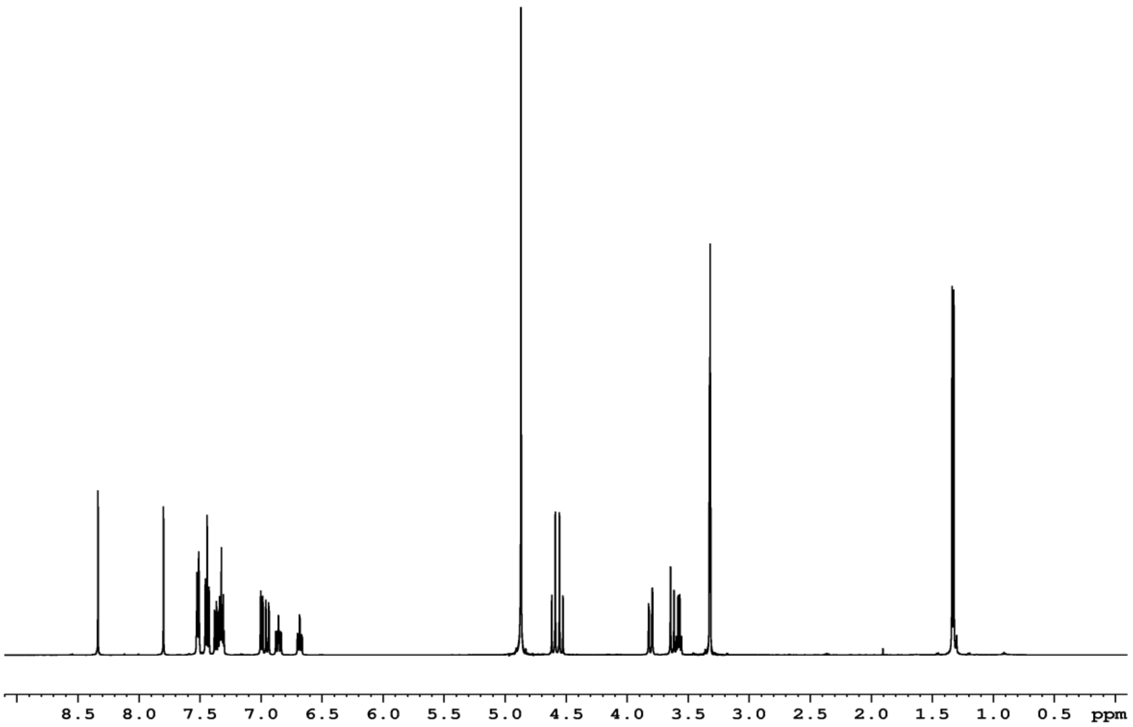

Figure S43. 500 MHz ^{1}H-NMR spectrum of hybrid **6** in CD_3OD at 298 K.

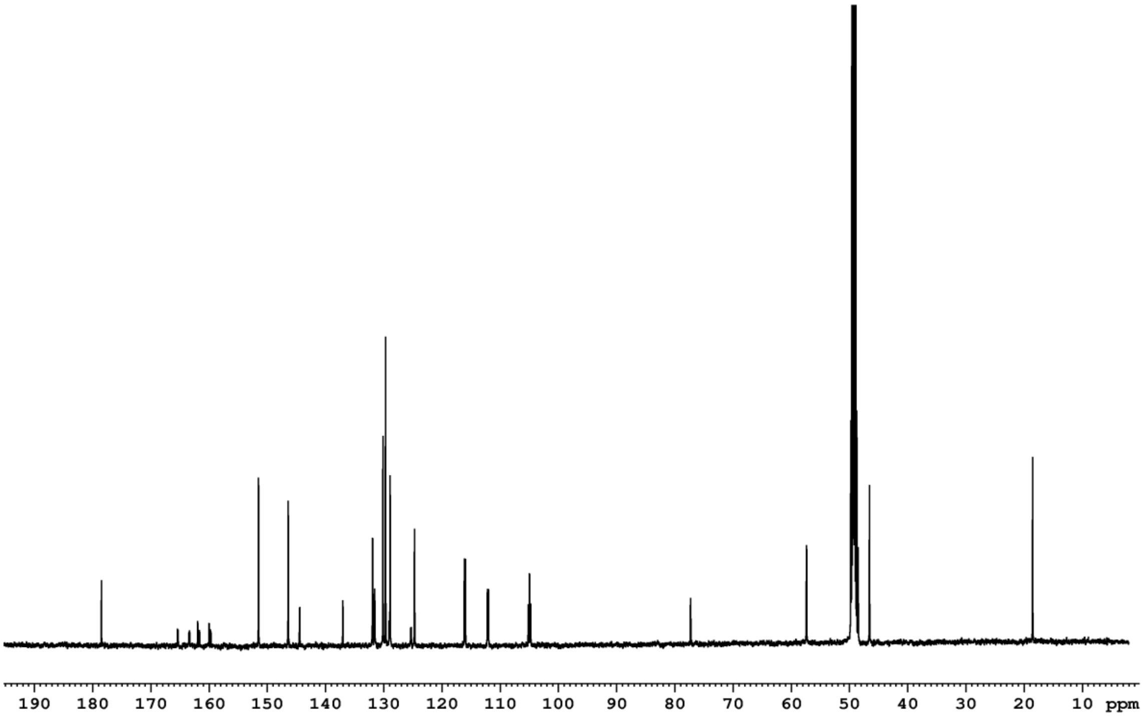

Figure S44. 125 MHz ^{13}C-NMR spectrum of hybrid **6** in CD_3OD at 298 K.

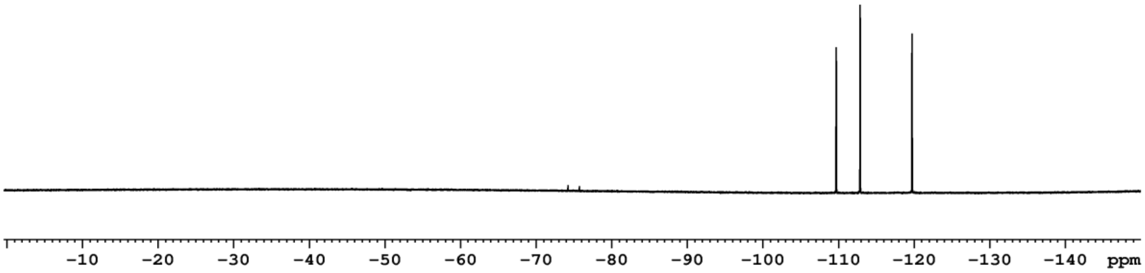

Figure S45. 470 MHz ^{19}F-NMR spectrum of hybrid **6** in CD_3OD at 298 K.

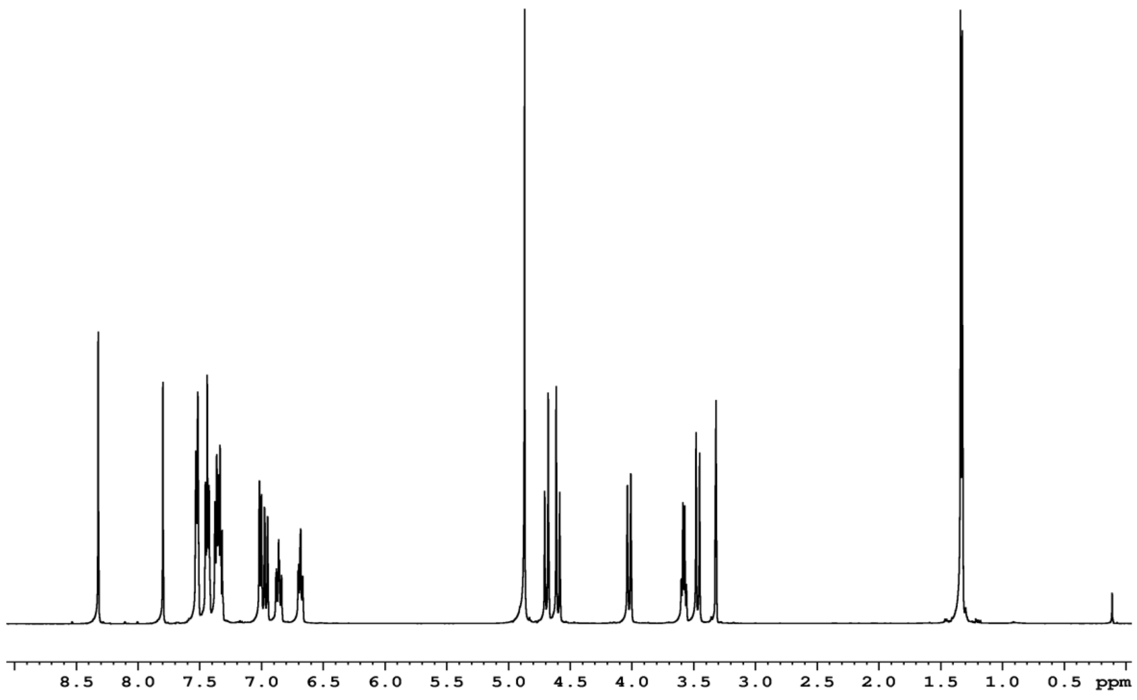

Figure S46. 500 MHz ^{1}H-NMR spectrum of hybrid **7** in CD_3OD at 298 K.

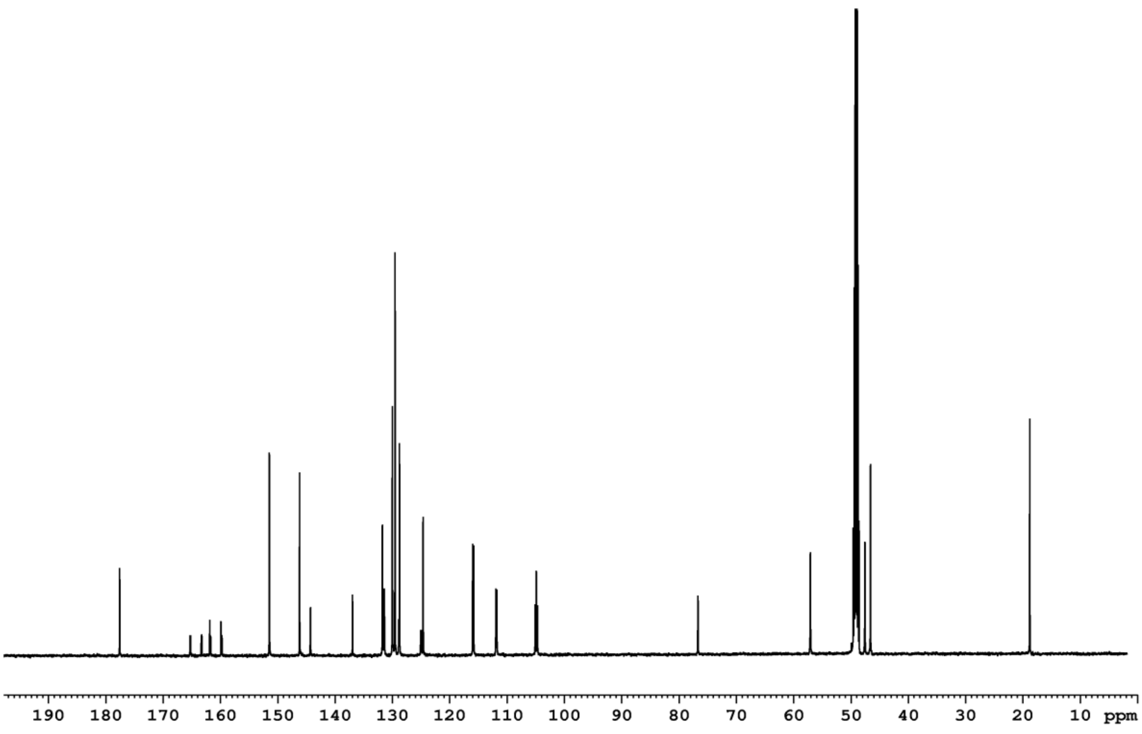

Figure S47. 125 MHz ^{13}C-NMR spectrum of hybrid **7** in CD_3OD at 298 K.

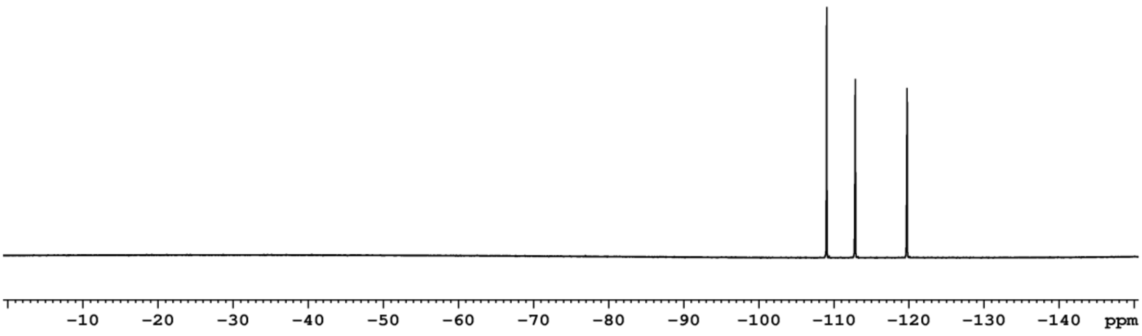

Figure S48. 470 MHz ^{19}F-NMR spectrum of hybrid **7** in CD_3OD at 298 K.

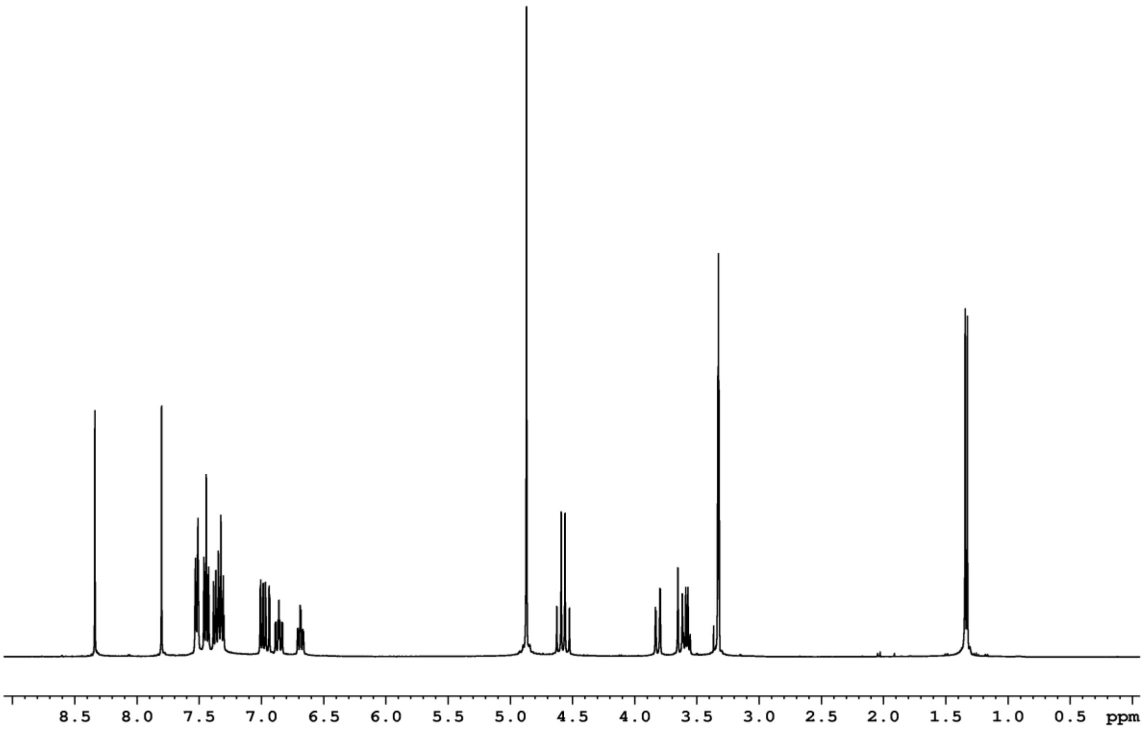

Figure S49. 400 MHz ^{1}H-NMR spectrum of hybrid **8** in CD_3OD at 298 K.

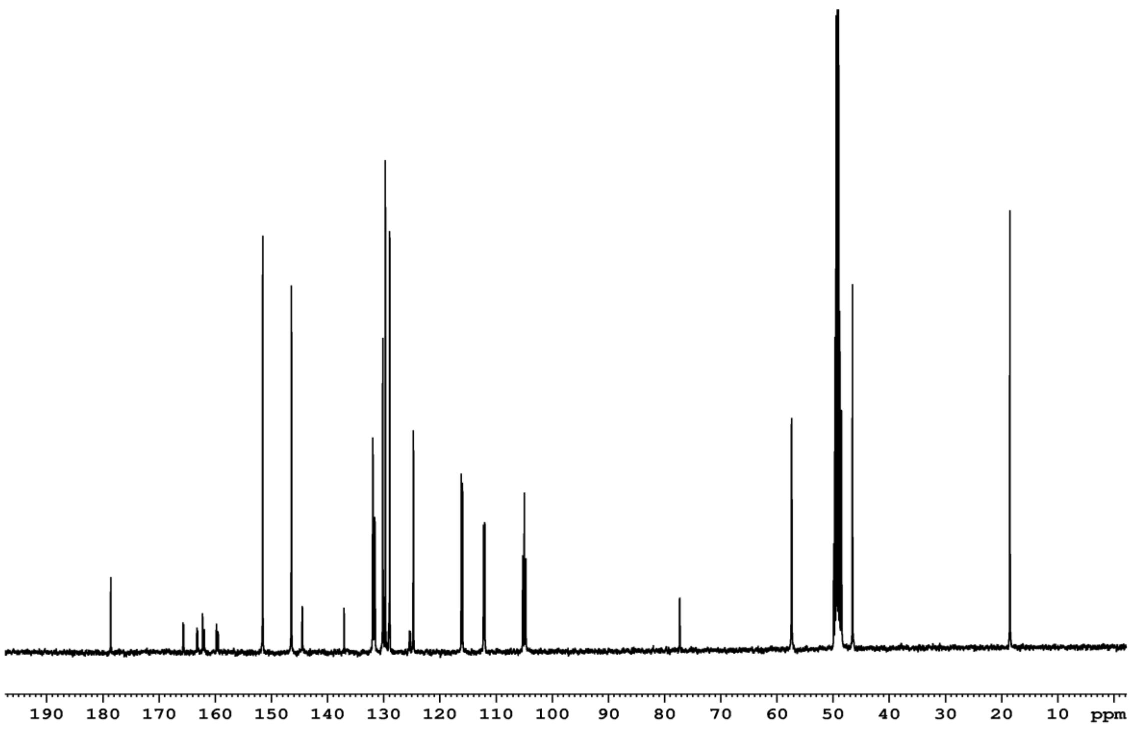

Figure S50. 100 MHz ^{13}C-NMR spectrum of hybrid **8** in CD_3OD at 298 K.

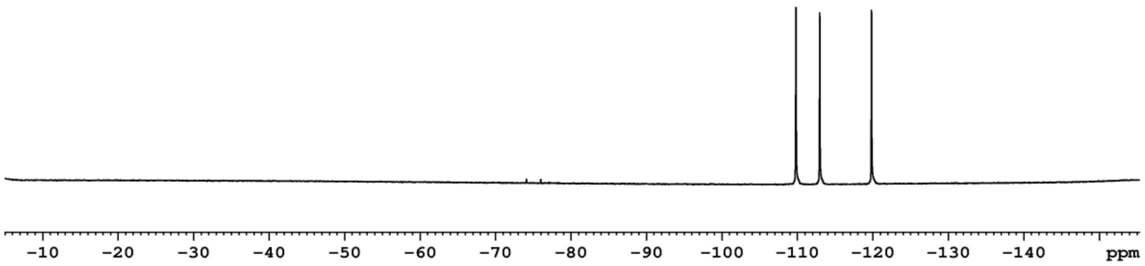

Figure S51. 375 MHz ^{19}F-NMR spectrum of hybrid **8** in CD_3OD at 298 K.

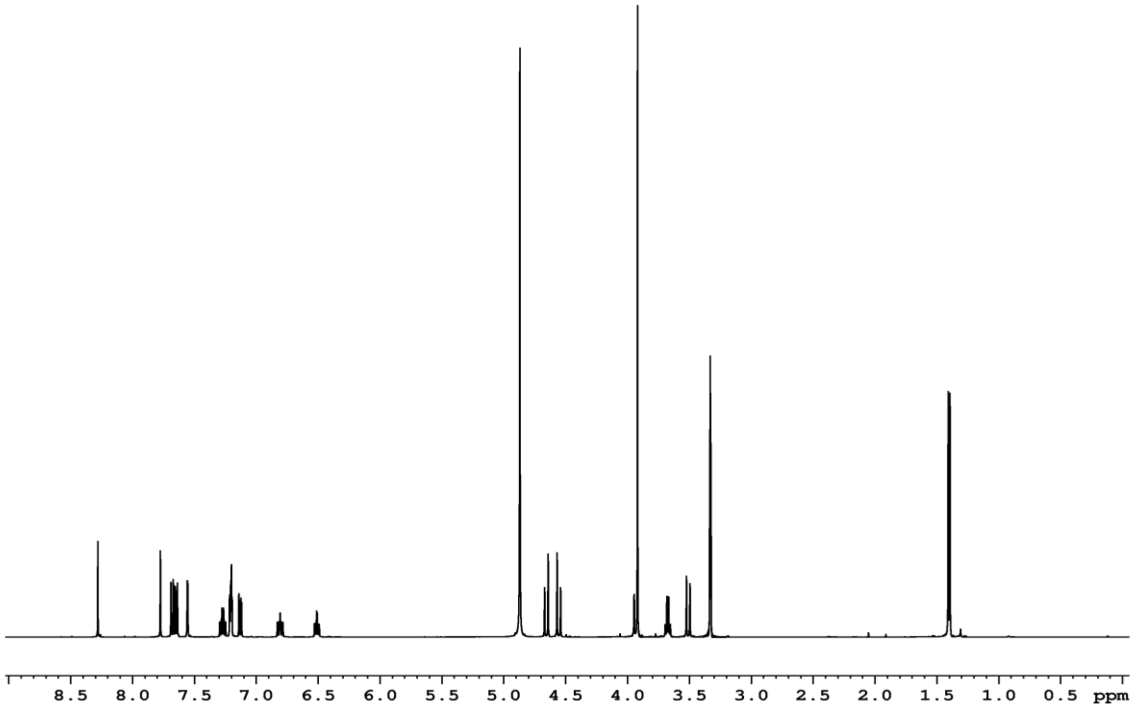

Figure S52. 500 MHz ^{1}H-NMR spectrum of hybrid **9** in CD_3OD at 298 K.

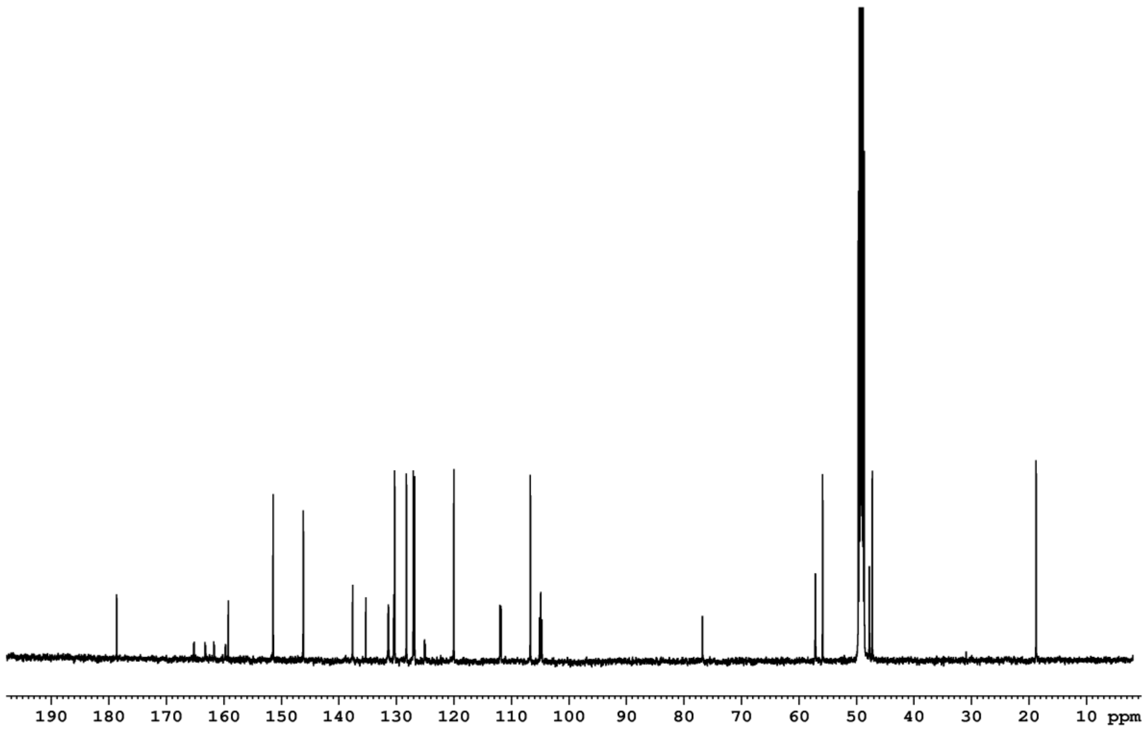

Figure S53. 125 MHz ^{13}C-NMR spectrum of hybrid **9** in CD_3OD at 298 K.

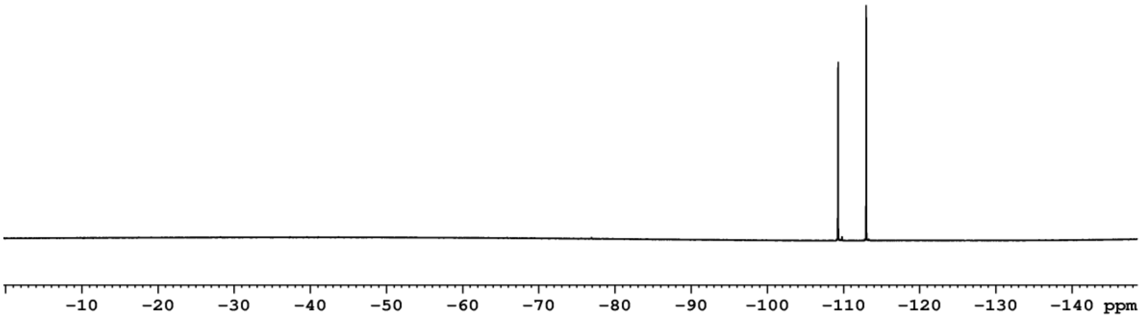

Figure S54. 470 MHz ^{19}F-NMR spectrum of hybrid **9** in CD_3OD at 298 K.

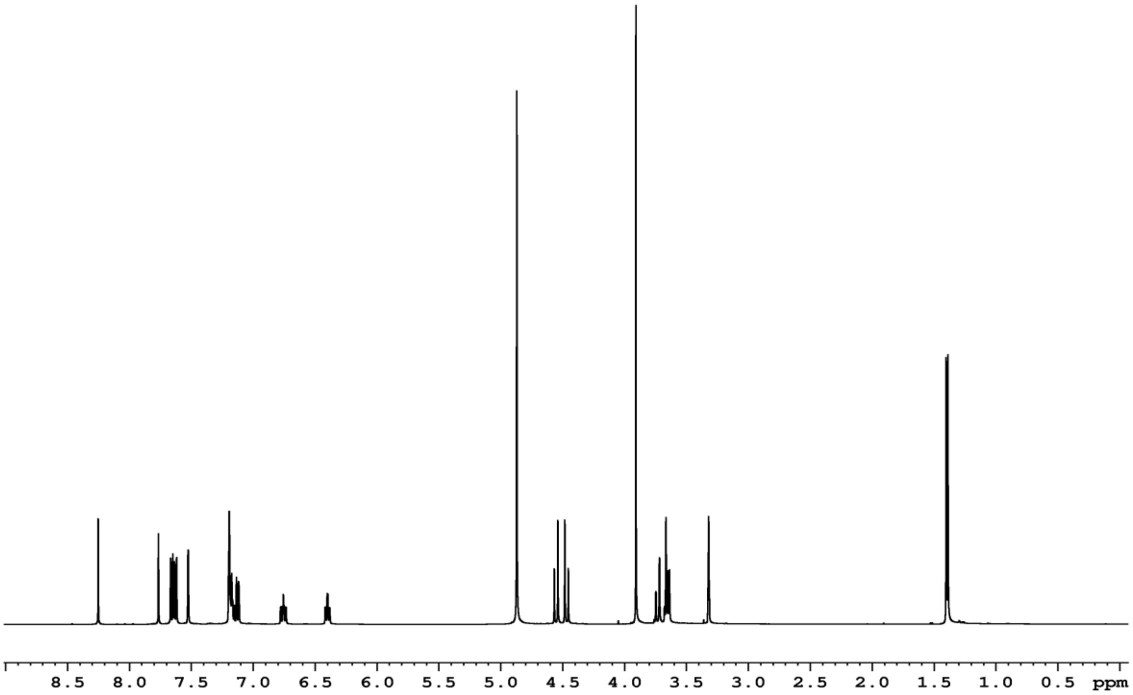

Figure S55. 500 MHz ^{1}H-NMR spectrum of hybrid **10** in CD_3OD at 298 K.

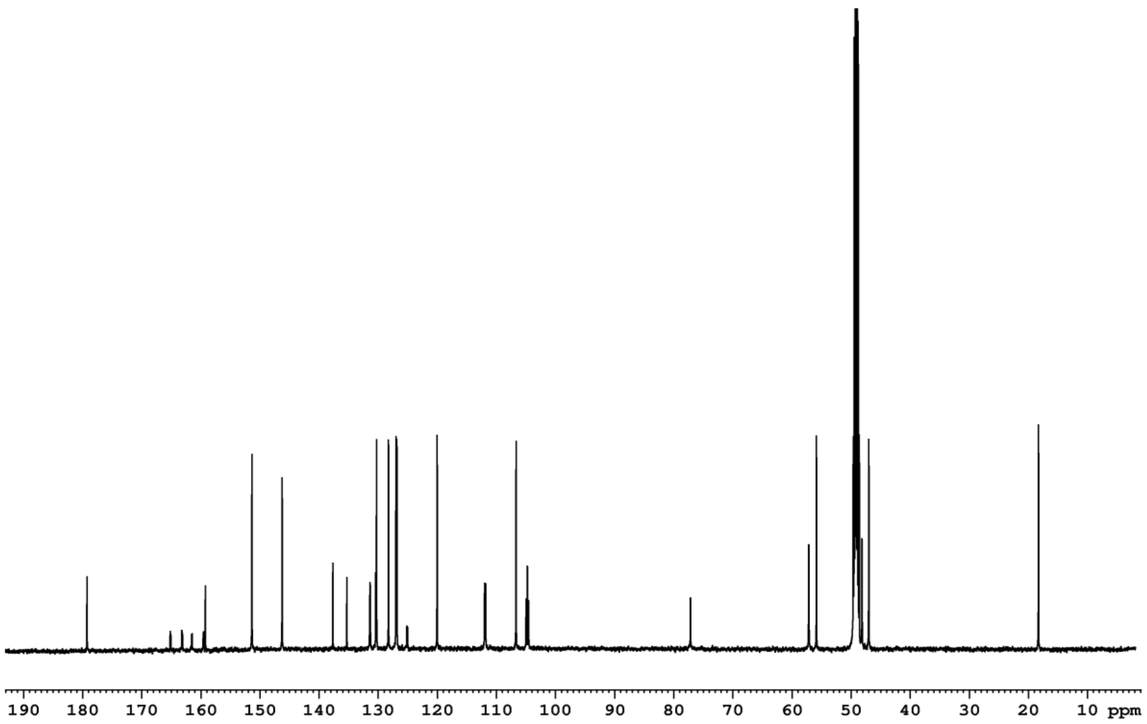

Figure S56. 125 MHz ^{13}C-NMR spectrum of hybrid **10** in CD_3OD at 298 K.

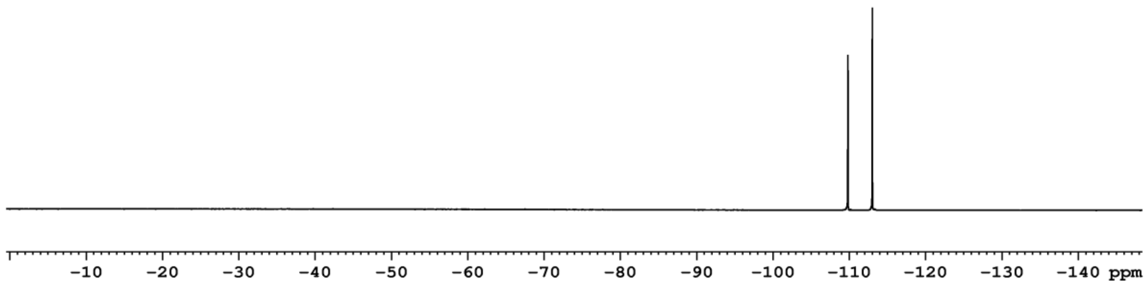

Figure S57. 470 MHz ^{19}F-NMR spectrum of hybrid **10** in CD_3OD at 298 K.

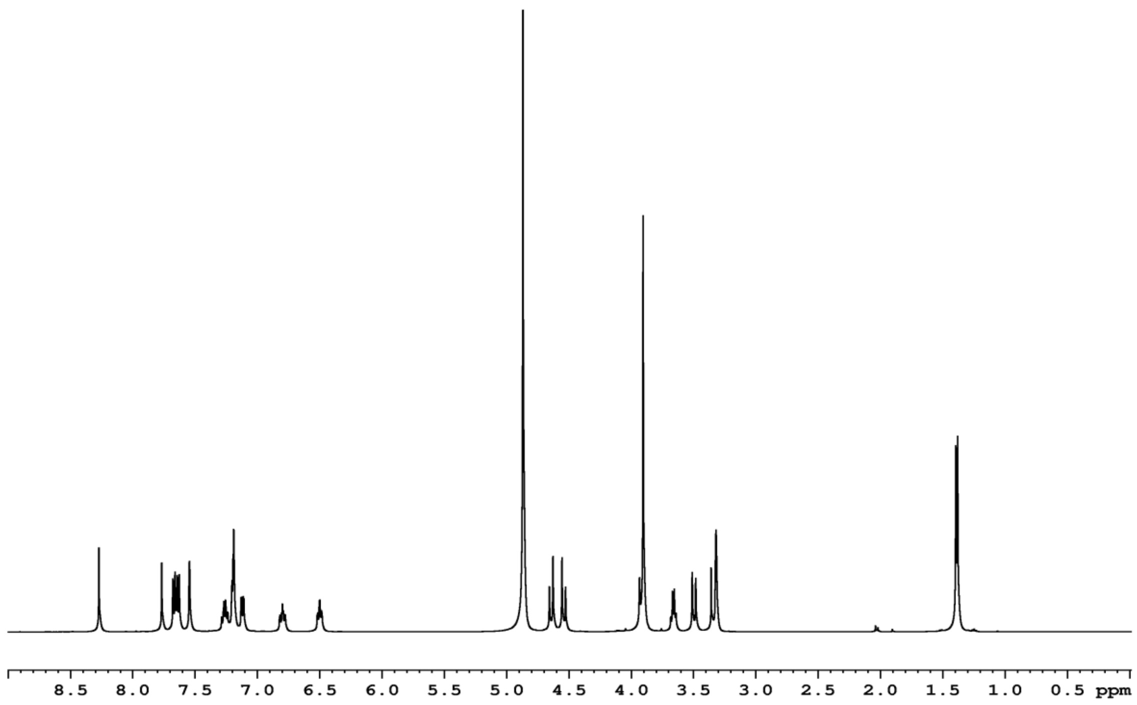

Figure S58. 500 MHz ^{1}H-NMR spectrum of hybrid **11** in $CDCl_3$ at 298 K.

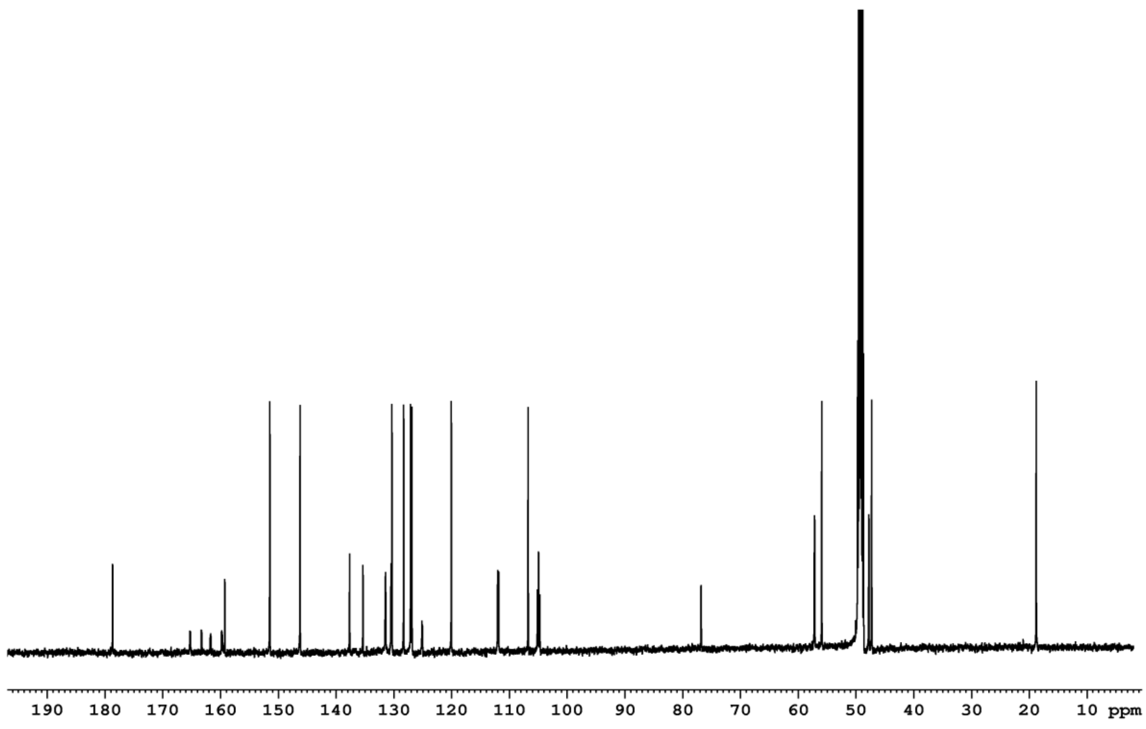

Figure S59. 125 MHz ^{13}C-NMR spectrum of hybrid **11** in $CDCl_3$ at 298 K.

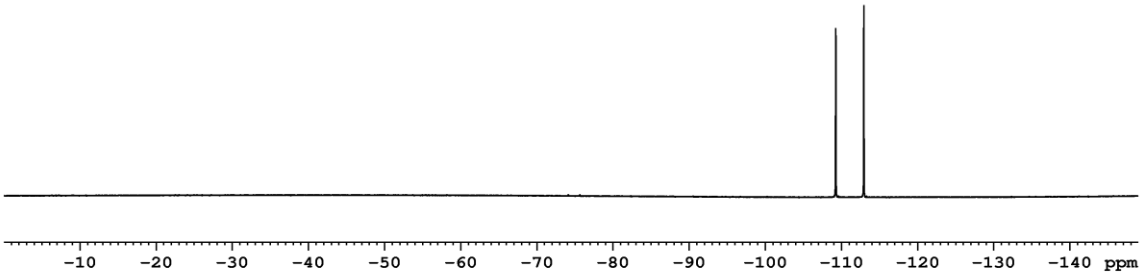

Figure S60. 470 MHz ^{19}F-NMR spectrum of hybrid **11** in CD_3OD at 298 K.

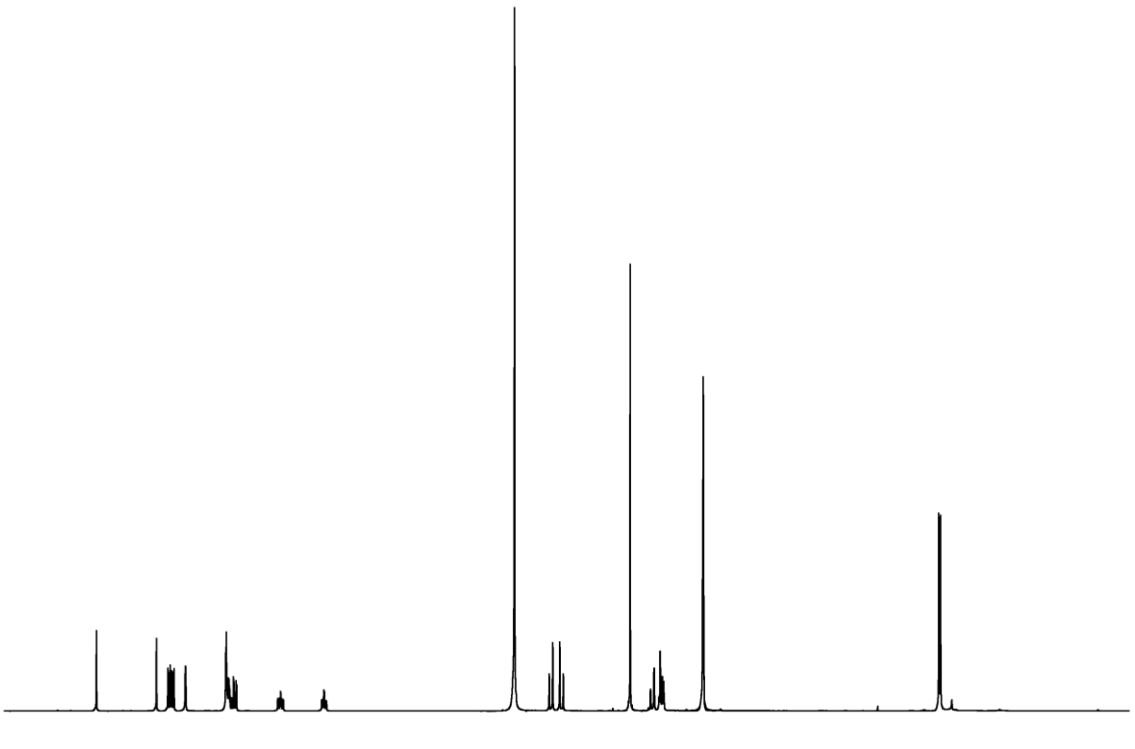

Figure S61. 500 MHz ^{1}H-NMR spectrum of hybrid **12** in CD_3OD at 298 K.

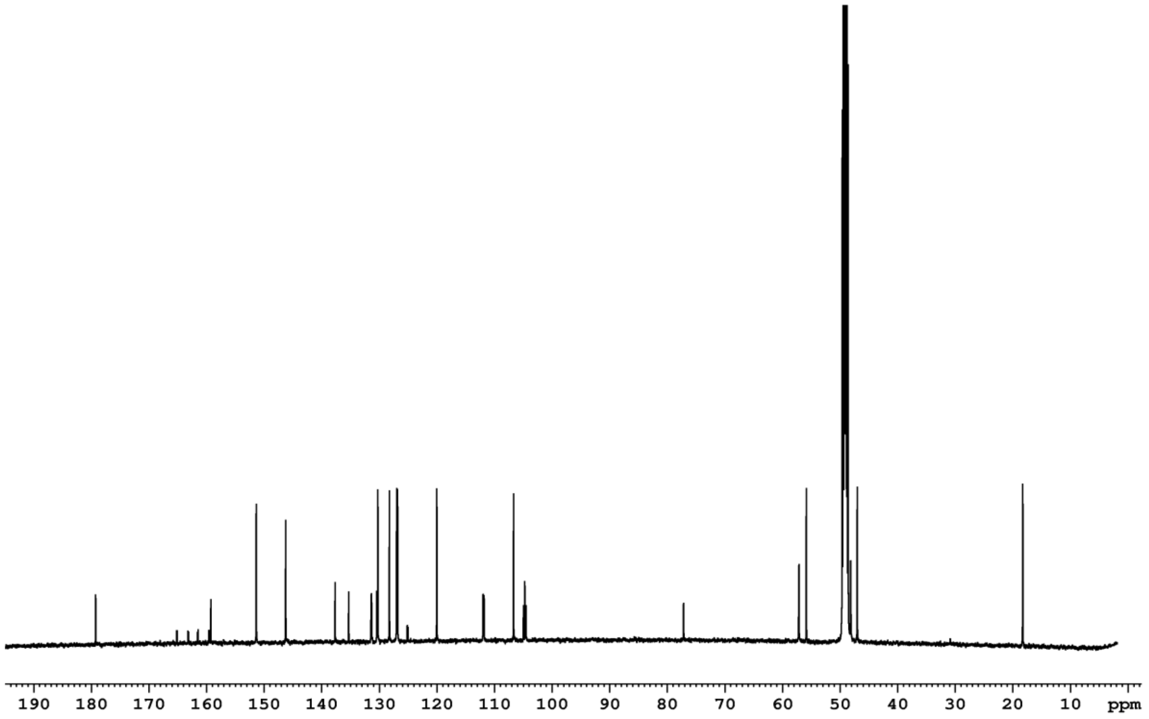

Figure S62. 125 MHz ^{13}C-NMR spectrum of hybrid **12** in CD_3OD at 298 K.

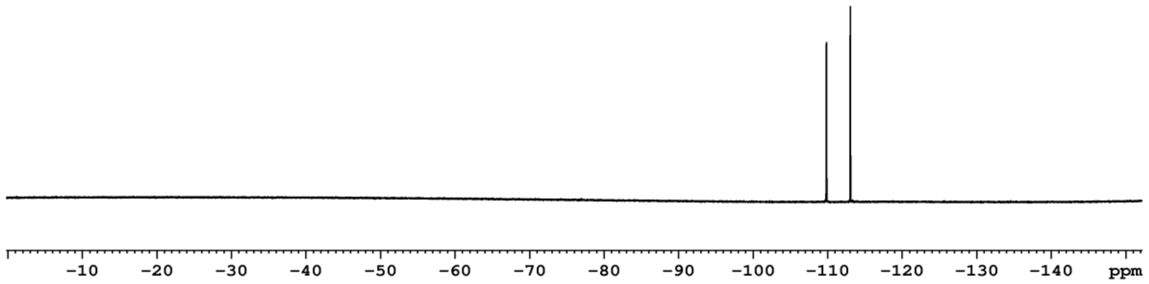

Figure S63. 470 MHz ^{19}F-NMR spectrum of hybrid **12** in CD_3OD at 298 K.

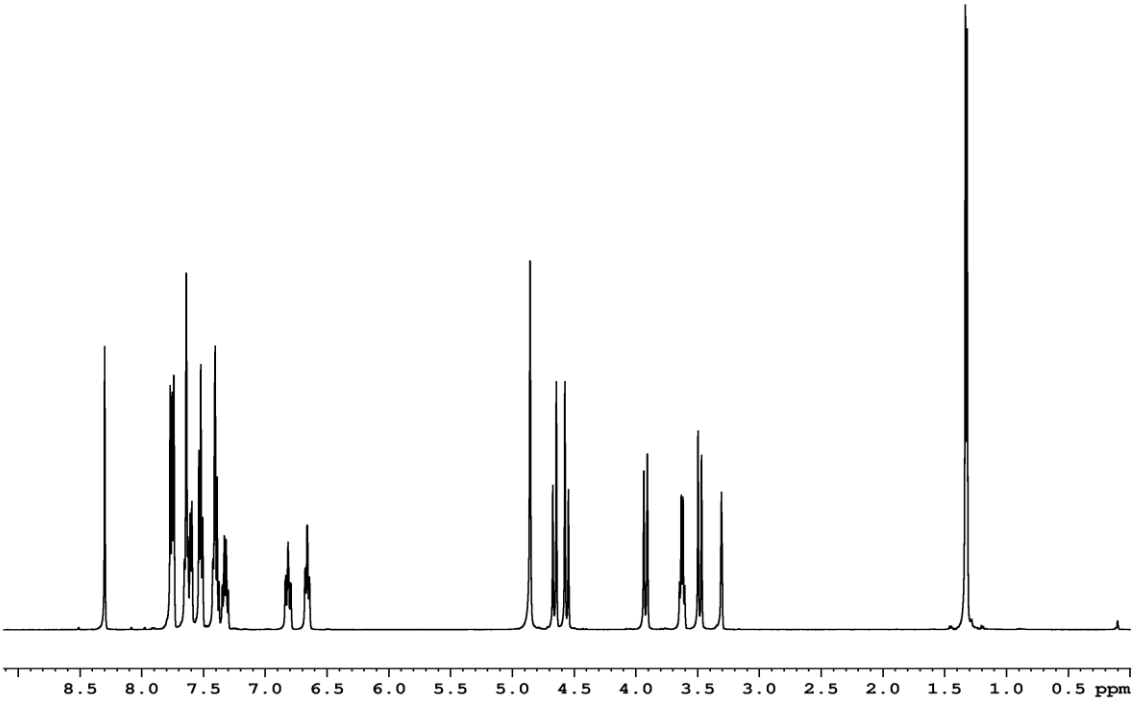

Figure S64. 500 MHz ^{1}H-NMR spectrum of hybrid **13** in CD_3OD at 298 K.

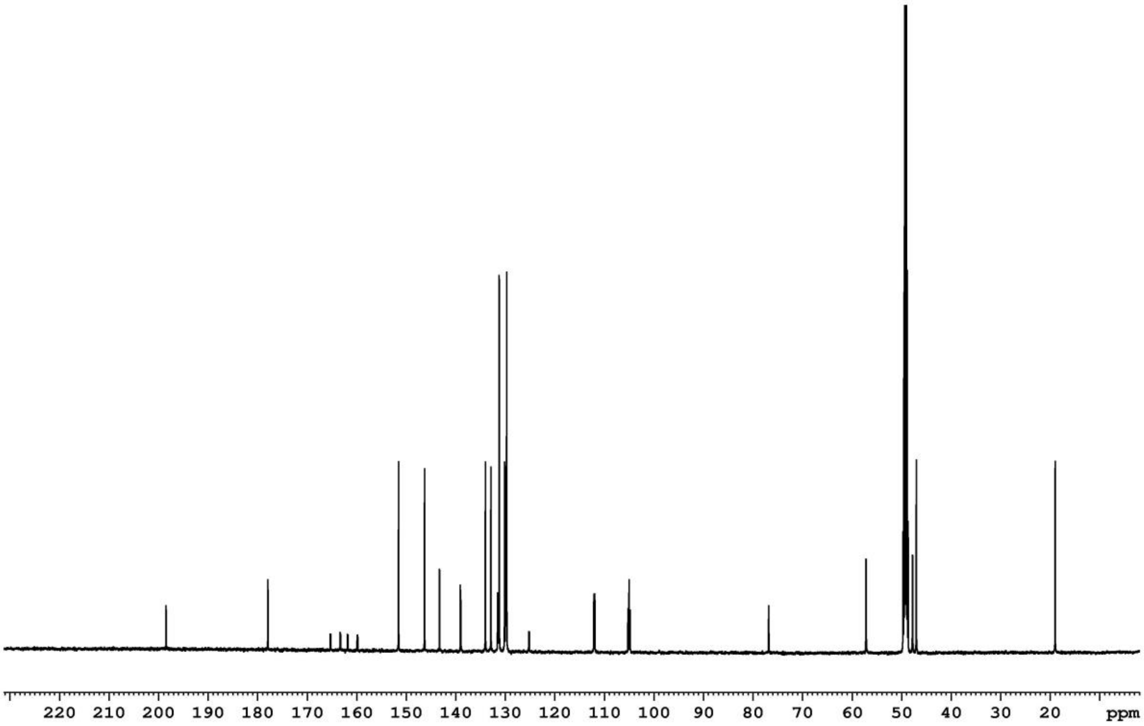

Figure S65. 125 MHz ^{13}C-NMR spectrum of hybrid **13** in CD_3OD at 298 K.

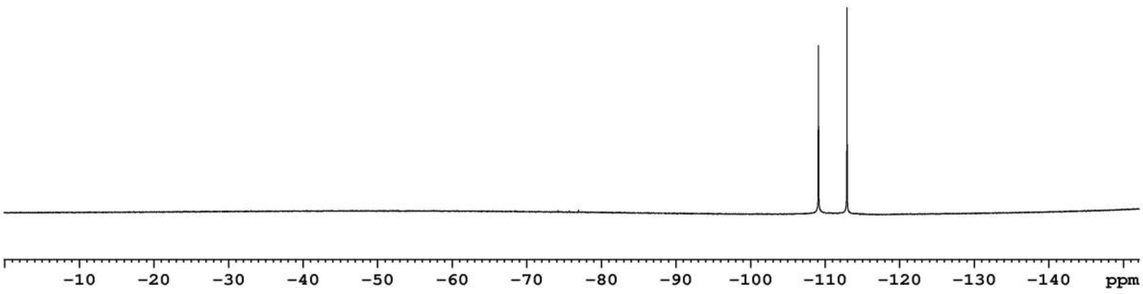

Figure S66. 470 MHz ^{19}F-NMR spectrum of hybrid **13** in CD_3OD at 298 K.

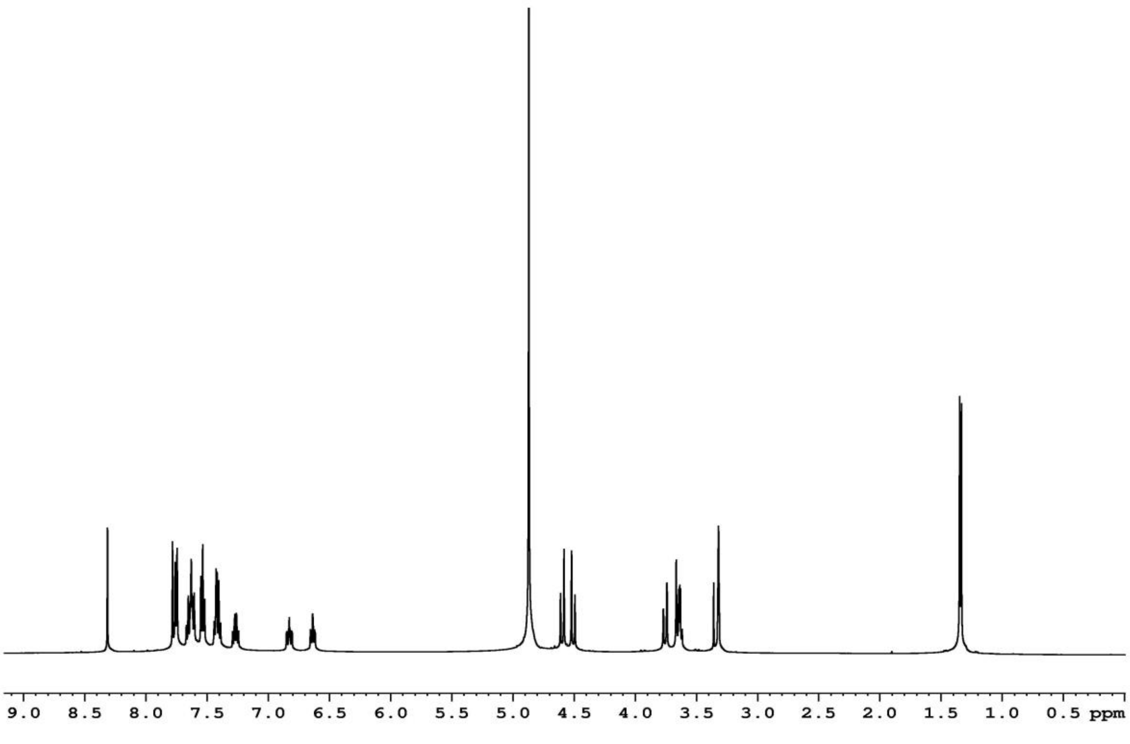

Figure S67. 500 MHz ^{1}H-NMR spectrum of hybrid **14** in CD_3OD at 298 K.

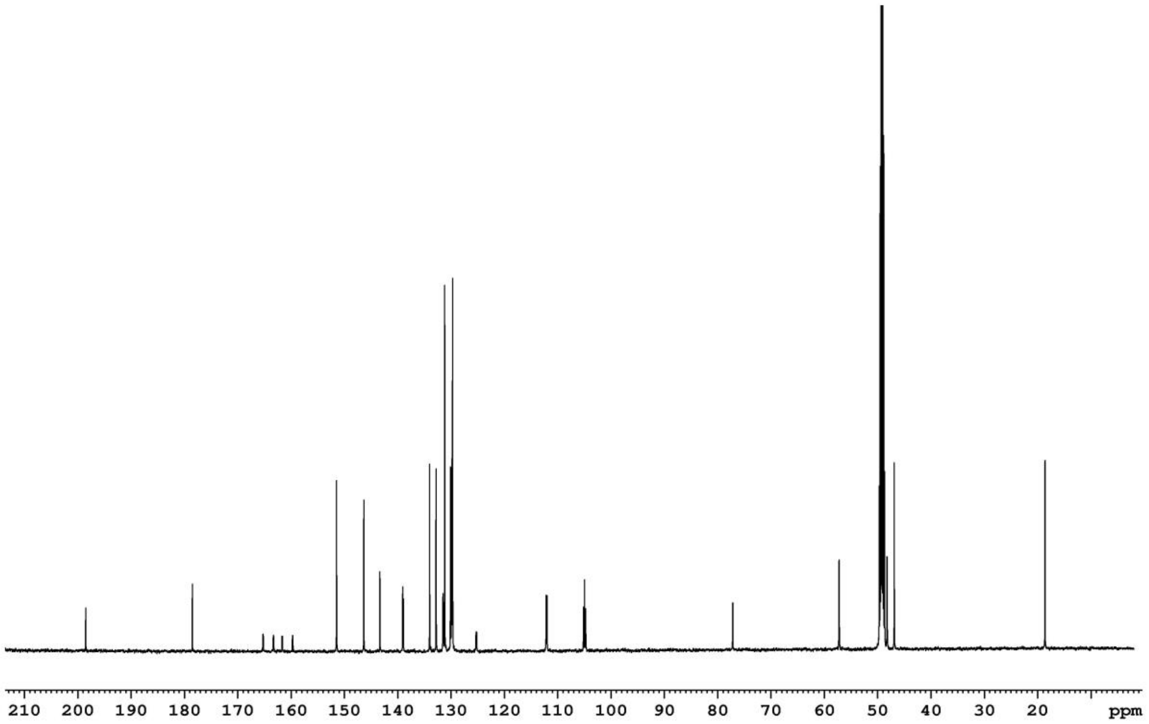

Figure S68. 125 MHz ^{13}C-NMR spectrum of hybrid **14** in CD_3OD at 298 K.

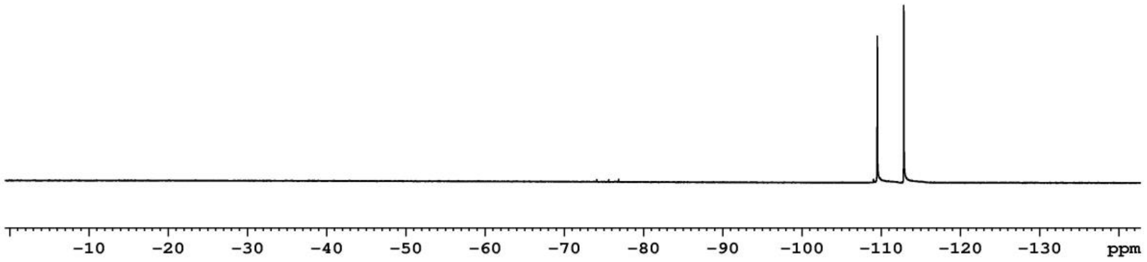

Figure S69. 470 MHz ^{19}F-NMR spectrum of hybrid **14** in CD_3OD at 298 K.

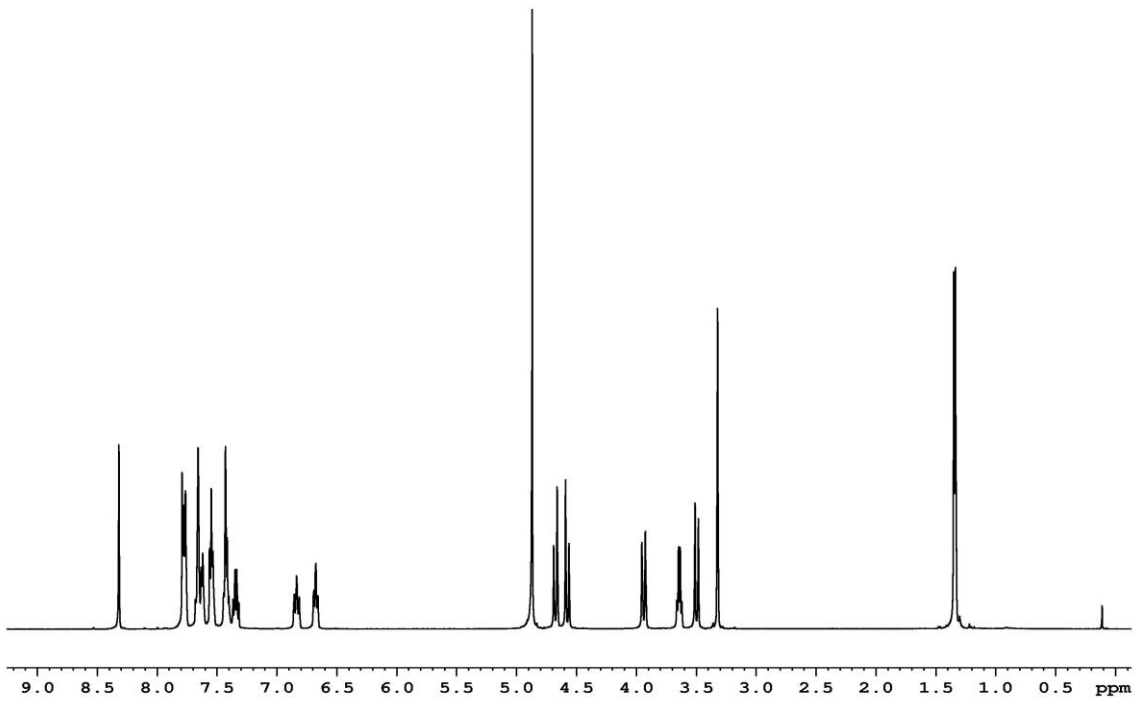

Figure S70. 500 MHz ^{1}H-NMR spectrum of hybrid **15** in CD_3OD at 298 K.

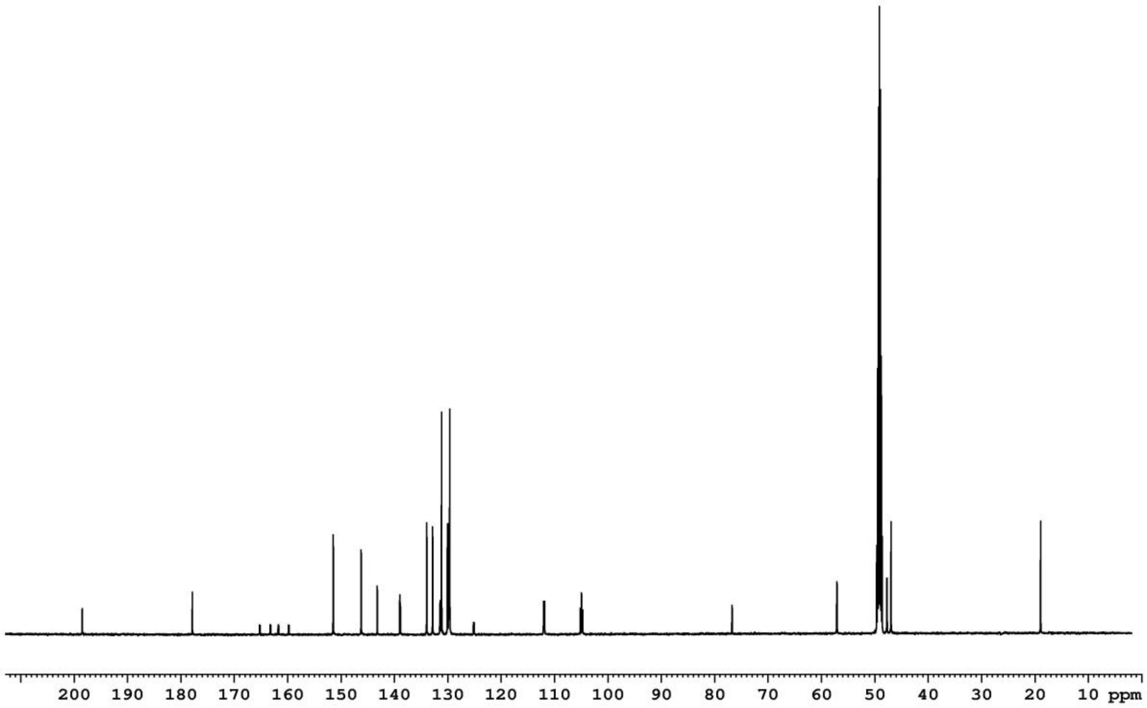

Figure S71. 125 MHz ^{13}C-NMR spectrum of hybrid **15** in CD_3OD at 298 K.

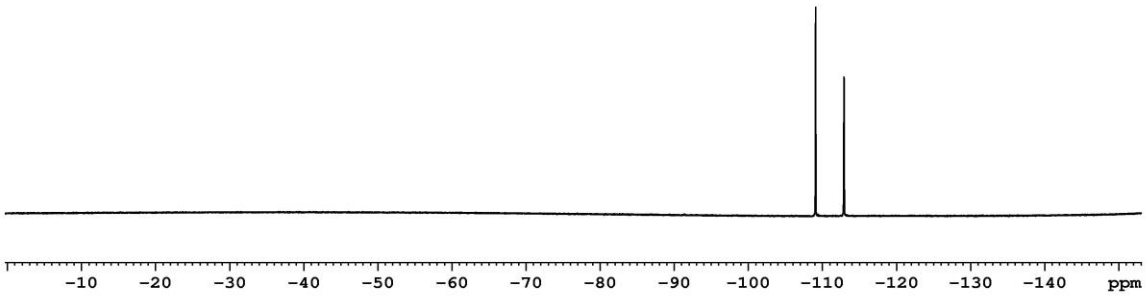

Figure S72. 470 MHz ^{19}F-NMR spectrum of hybrid **15** in CD_3OD at 298 K.

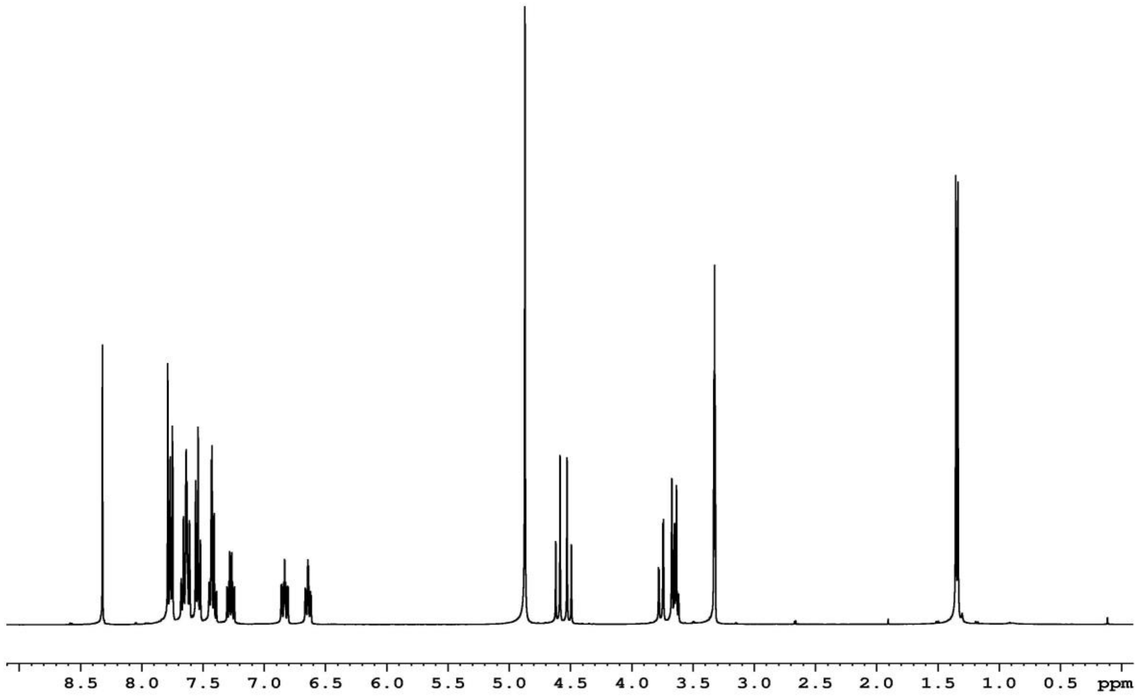

Figure S73. 400 MHz ^{1}H-NMR spectrum of hybrid **16** in CD_3OD at 298 K.

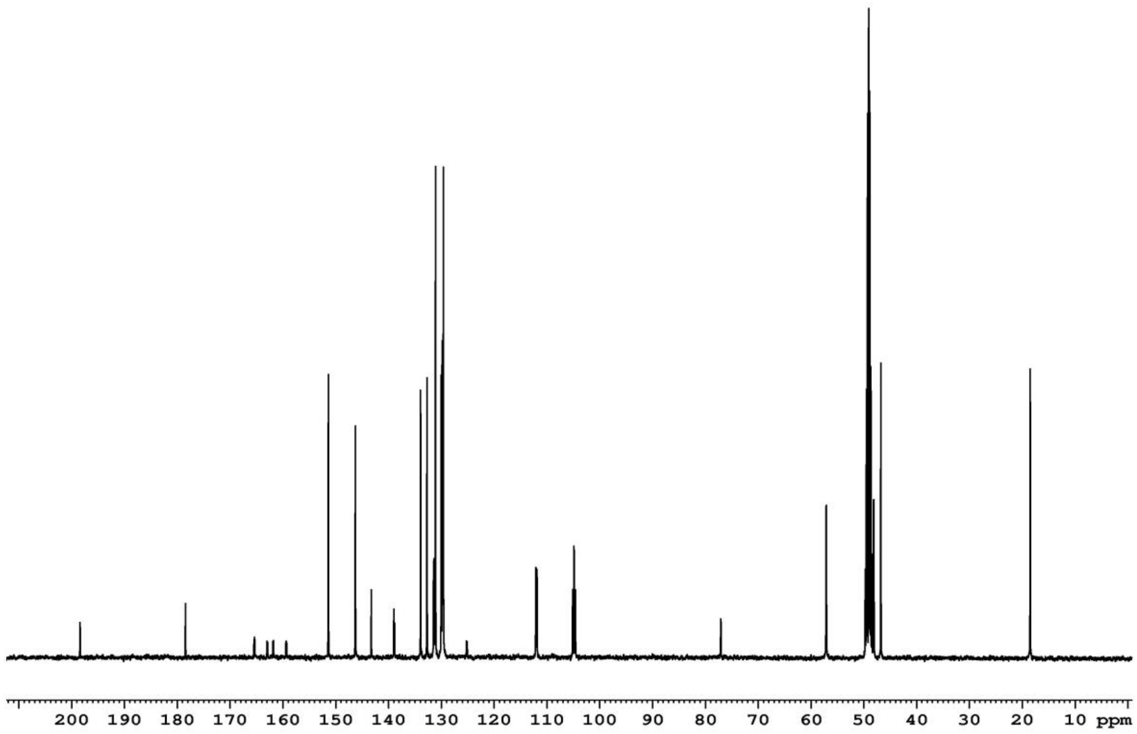

Figure S74. 100 MHz ^{13}C-NMR spectrum of hybrid **16** in CD_3OD at 298 K.

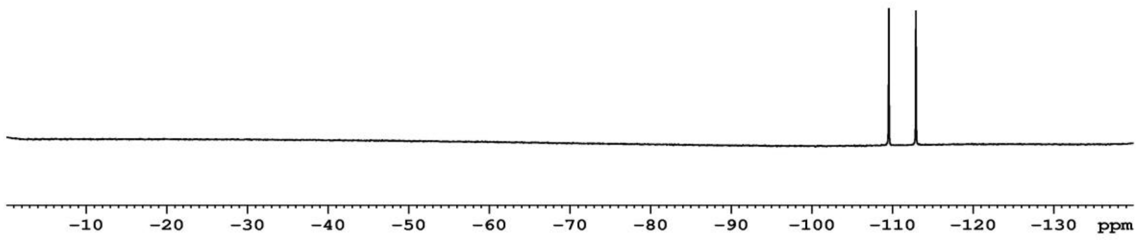

Figure S75. 375 MHz ^{19}F-NMR spectrum of hybrid **16** in CD_3OD at 298 K.

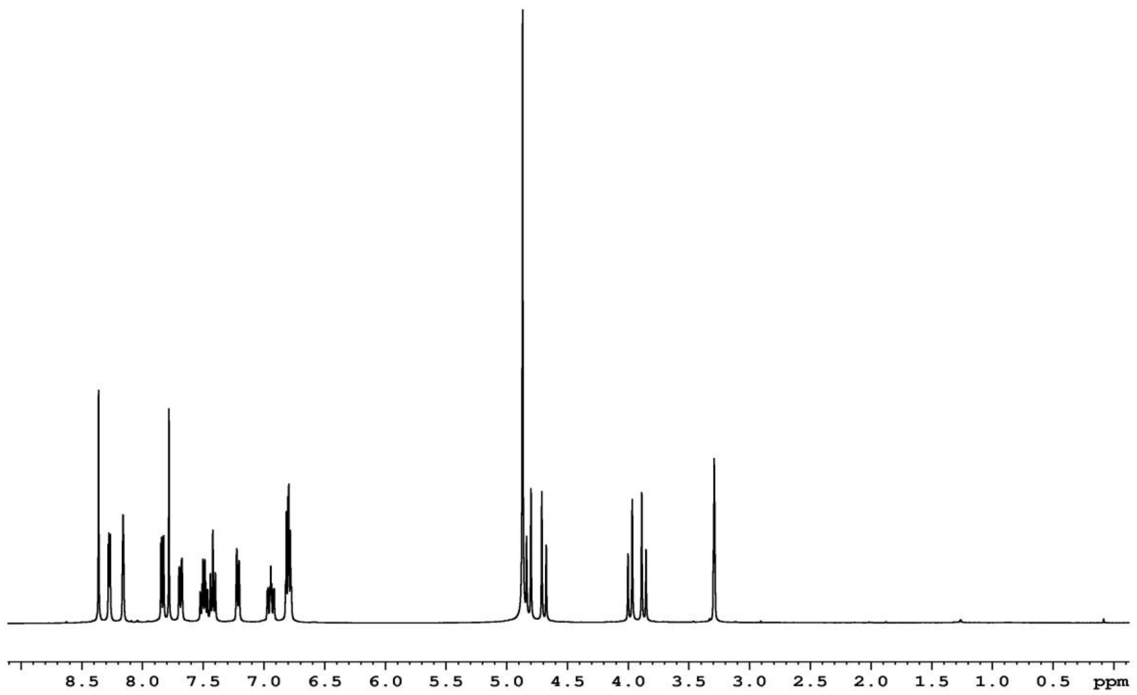

Figure S76. 400 MHz ^{1}H-NMR spectrum of hybrid **17** in CD_3OD at 298 K.

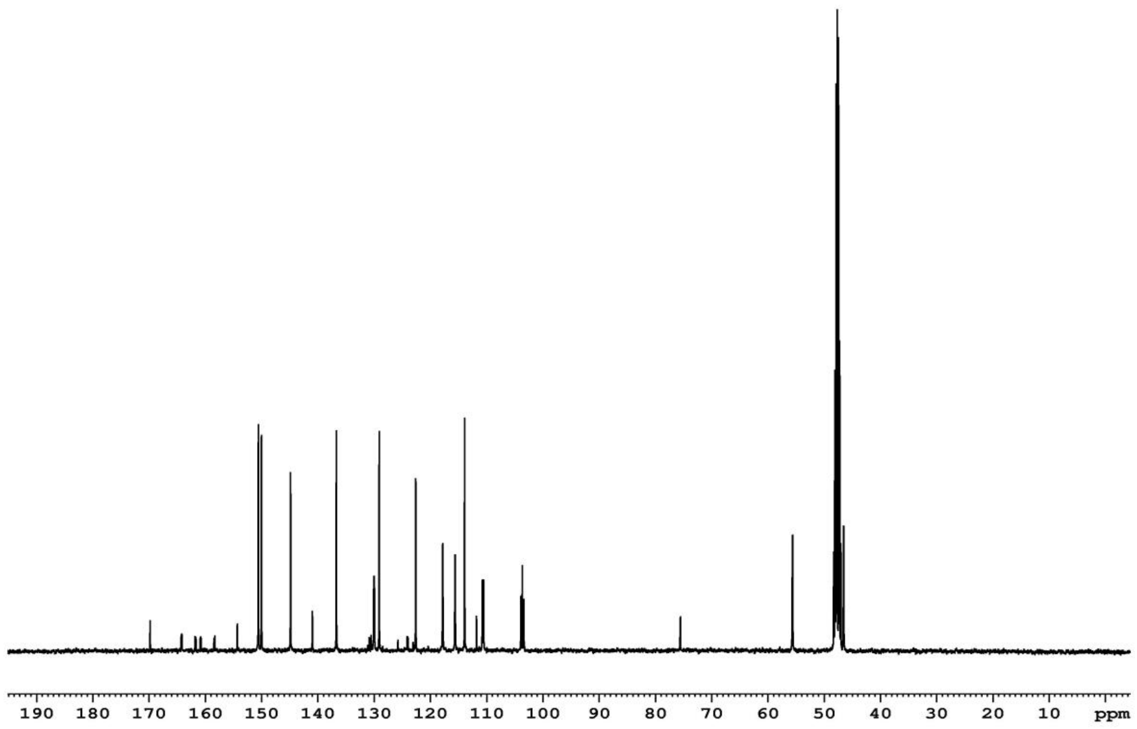

Figure S77. 100 MHz ^{13}C-NMR spectrum of hybrid **17** in CD_3OD at 298 K.

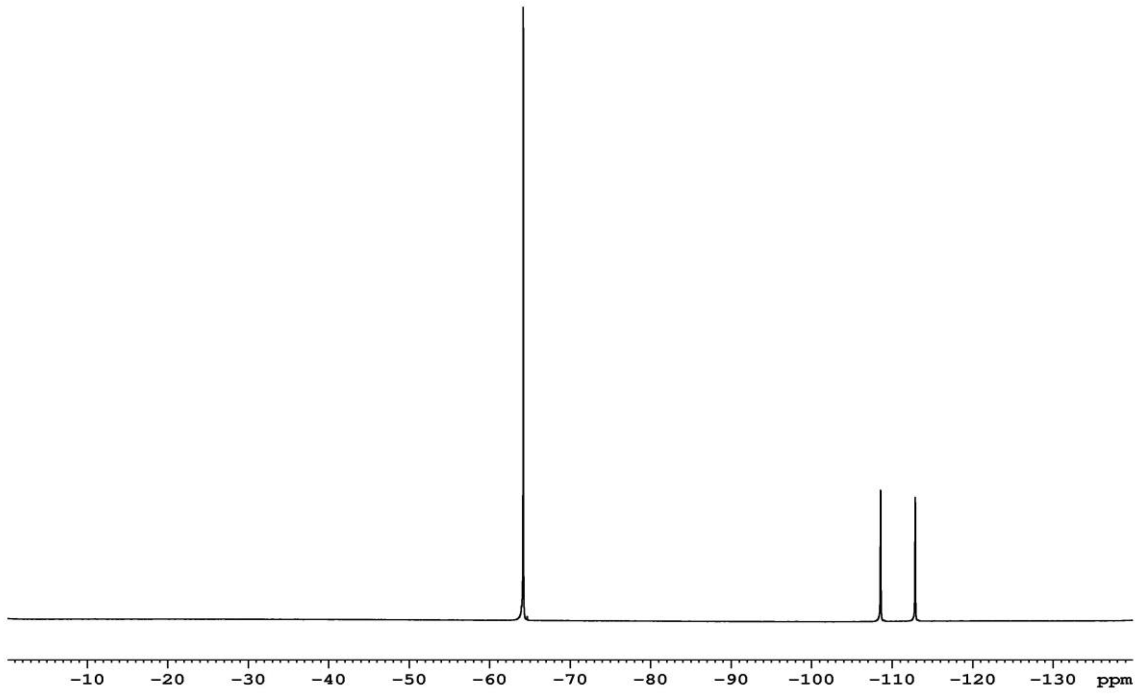

Figure S78. 375 MHz ^{19}F-NMR spectrum of hybrid **17** in CD_3OD at 298 K.

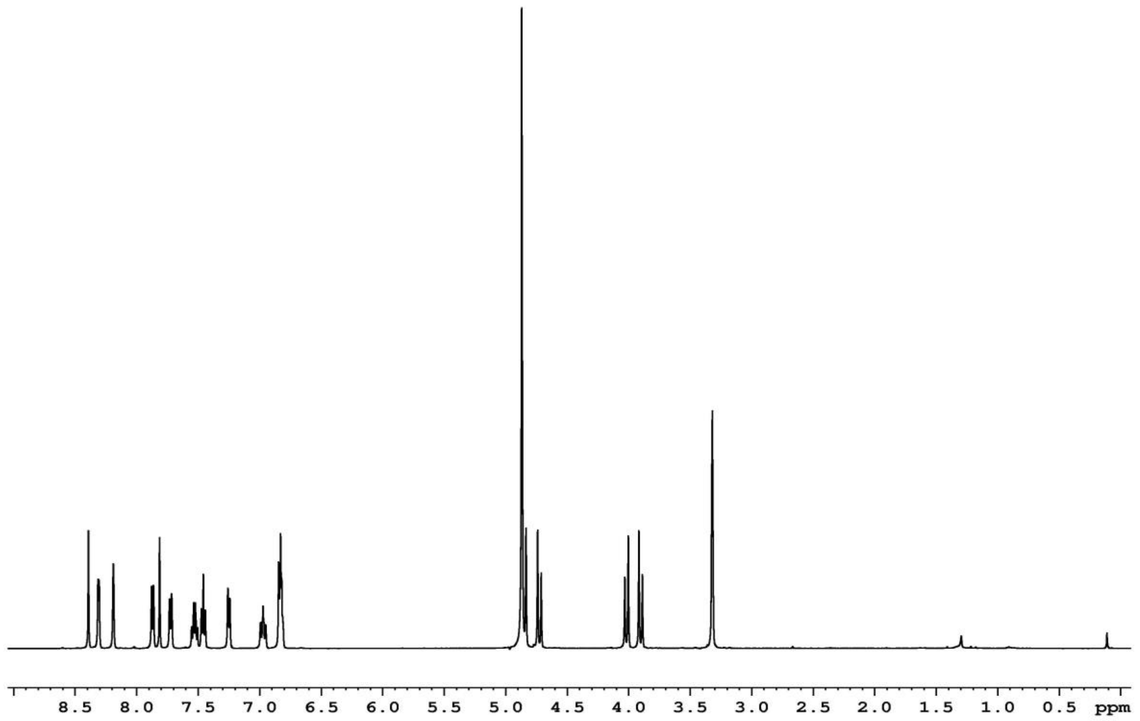

Figure S79. 500 MHz ^{1}H-NMR spectrum of hybrid **18** in CD_3OD at 298 K.

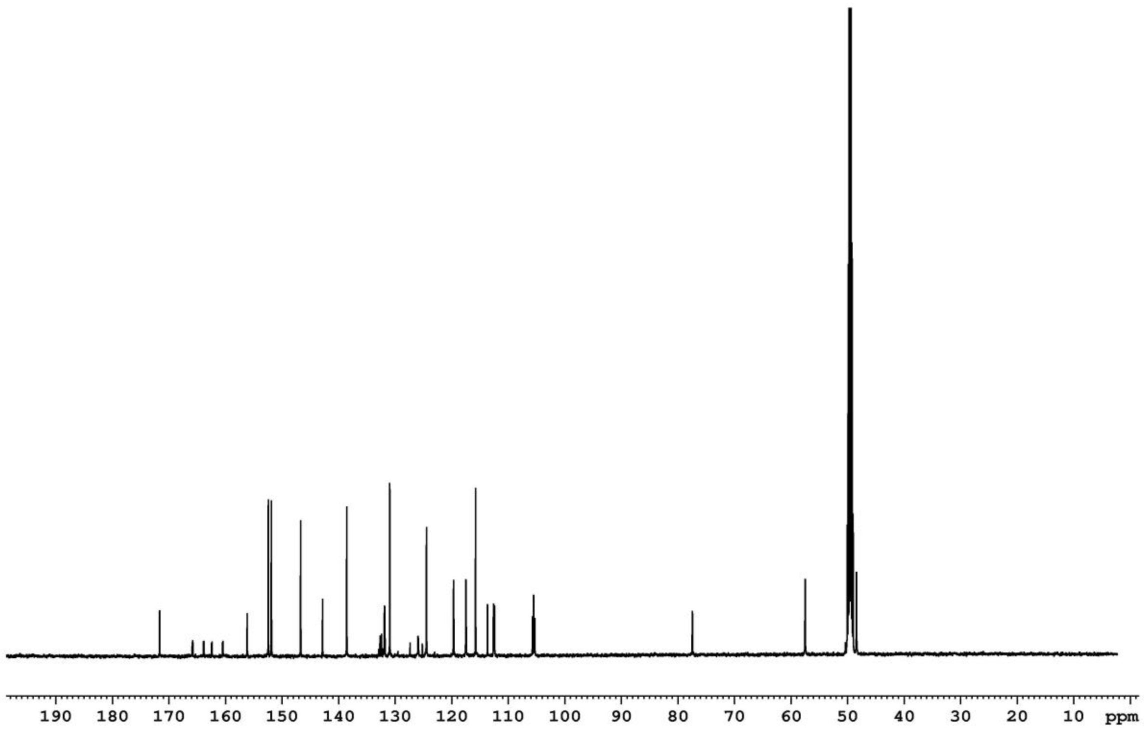

Figure S80. 125 MHz ^{13}C-NMR spectrum of hybrid **18** in CD_3OD at 298 K.

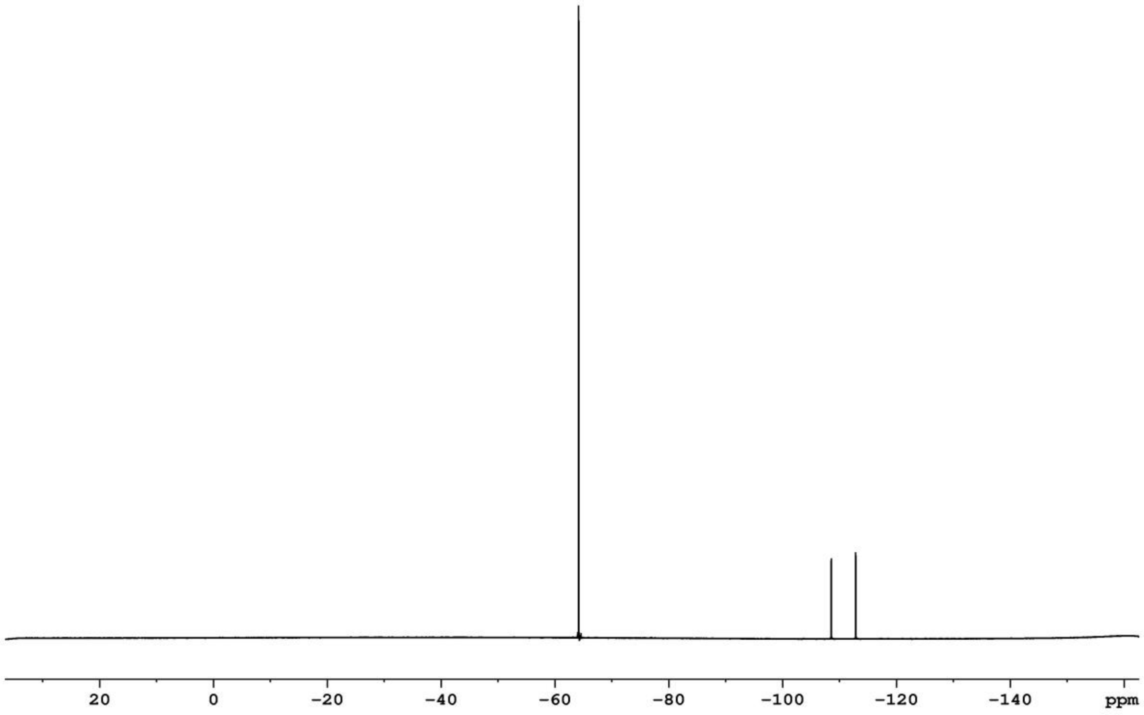

Figure S81. 470 MHz ^{19}F-NMR spectrum of hybrid **18** in CD_3OD at 298 K.

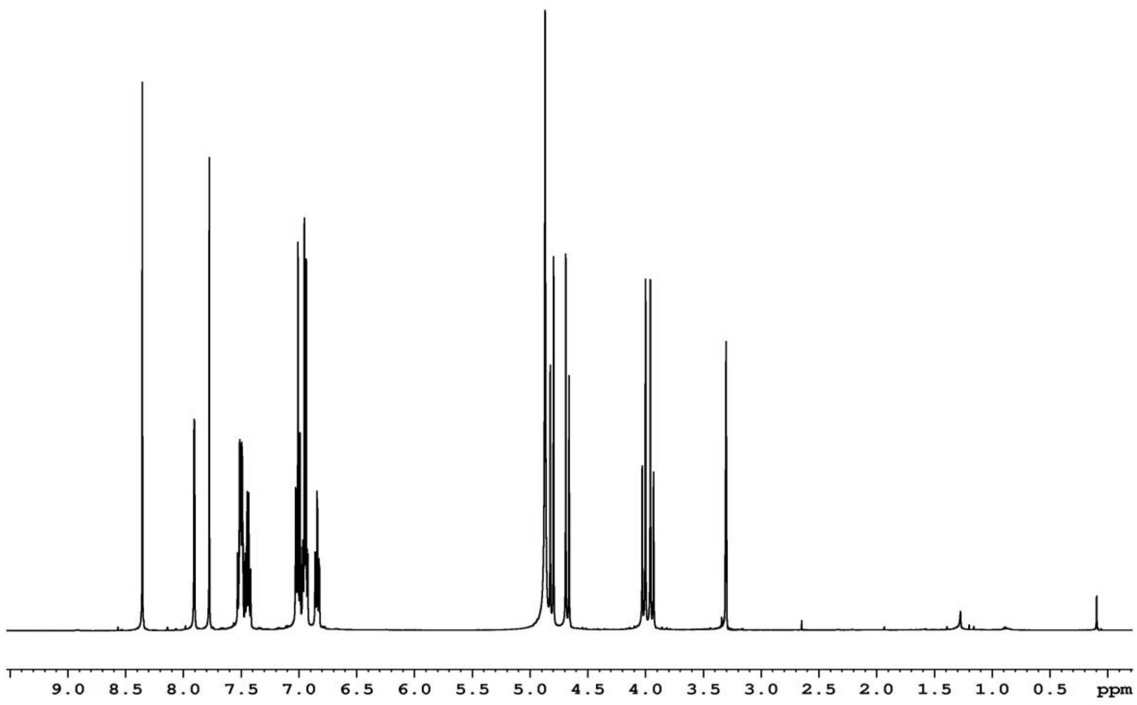

Figure S82. 500 MHz ^{1}H-NMR spectrum of hybrid **19** in CD_3OD at 298 K.

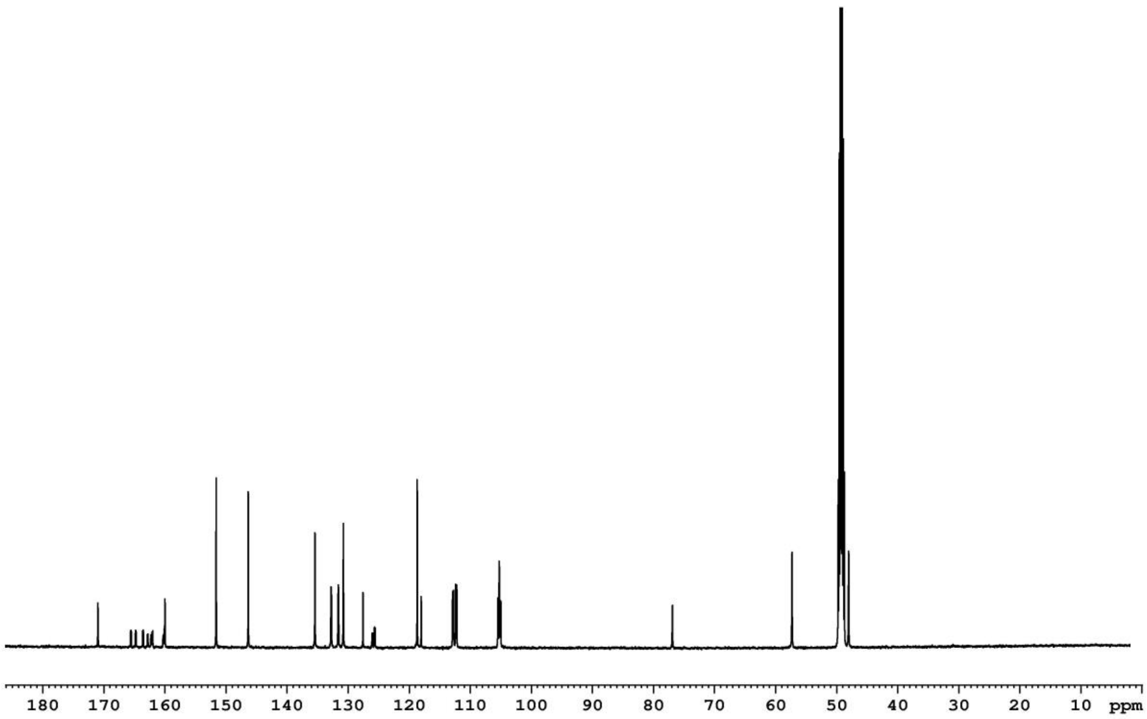

Figure S83. 125 MHz ^{13}C-NMR spectrum of hybrid **19** in $CDCl_3$ at 298 K.

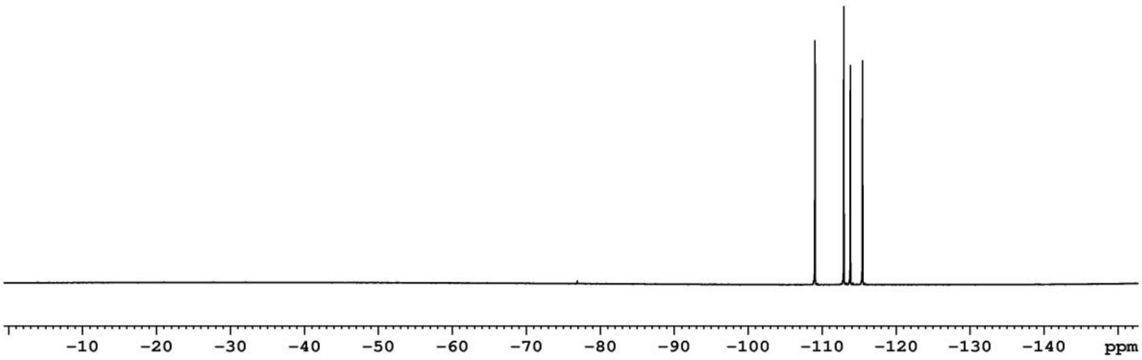

Figure S84. 470 MHz ^{19}F-NMR spectrum of hybrid **19** in CD_3OD at 298 K.

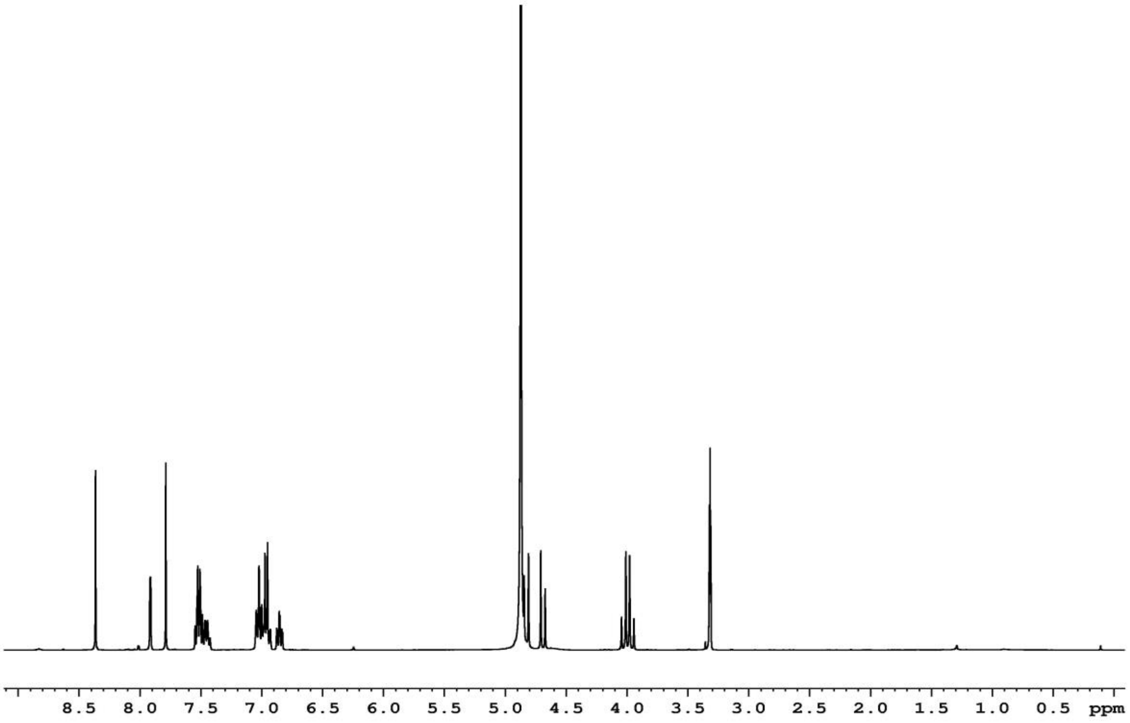

Figure S85. 400 MHz ^{1}H-NMR spectrum of hybrid **20** in CD_3OD at 298 K.

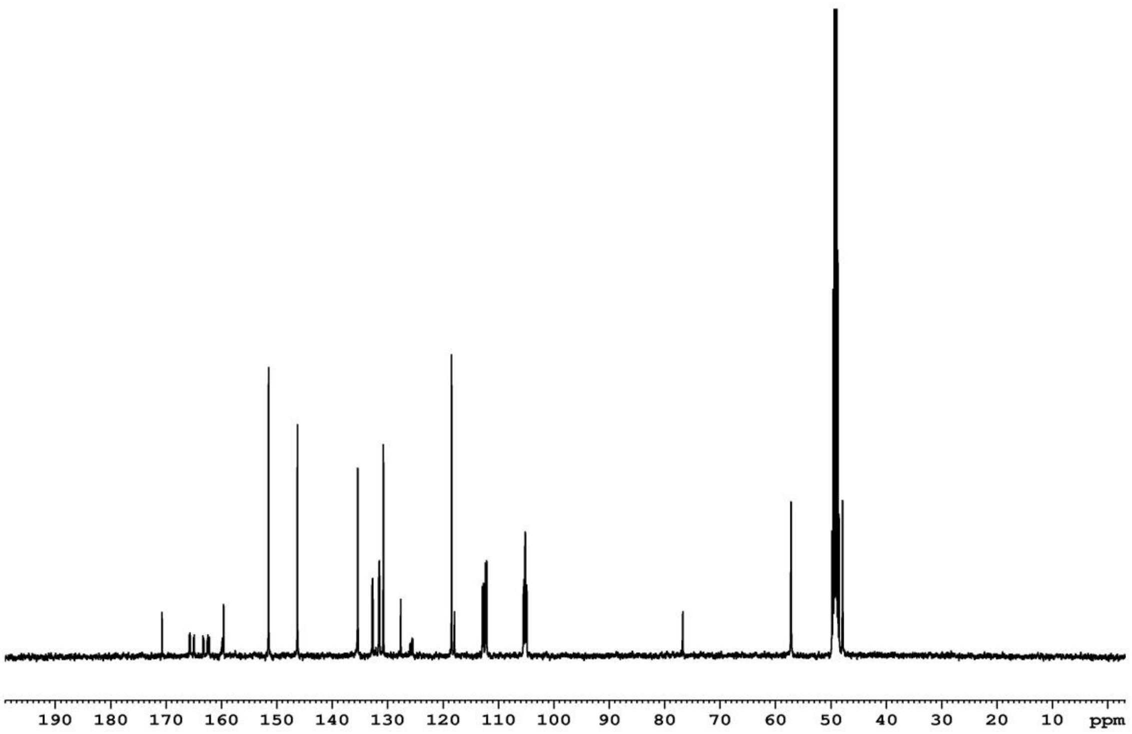

Figure S86. 100 MHz ^{13}C-NMR spectrum of hybrid **20** in CD_3OD at 298 K.

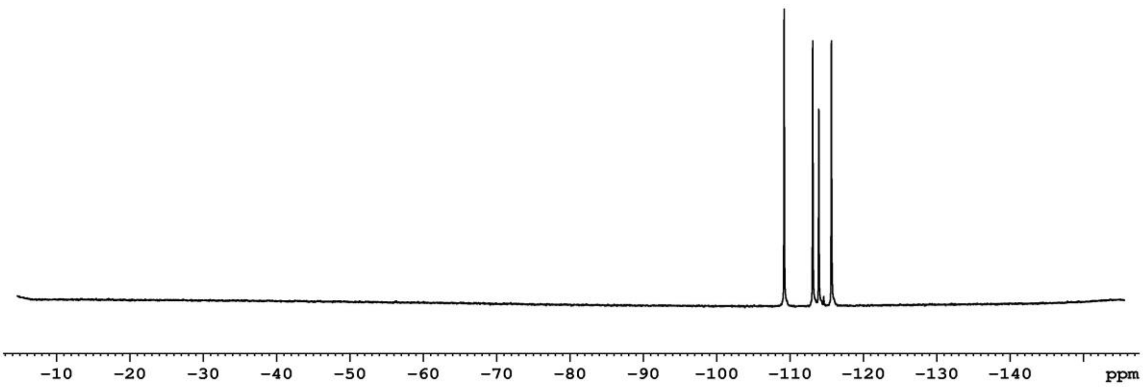

Figure S87. 375 MHz ^{19}F-NMR spectrum of Hybrid **20** in CD_3OD at 298 K.

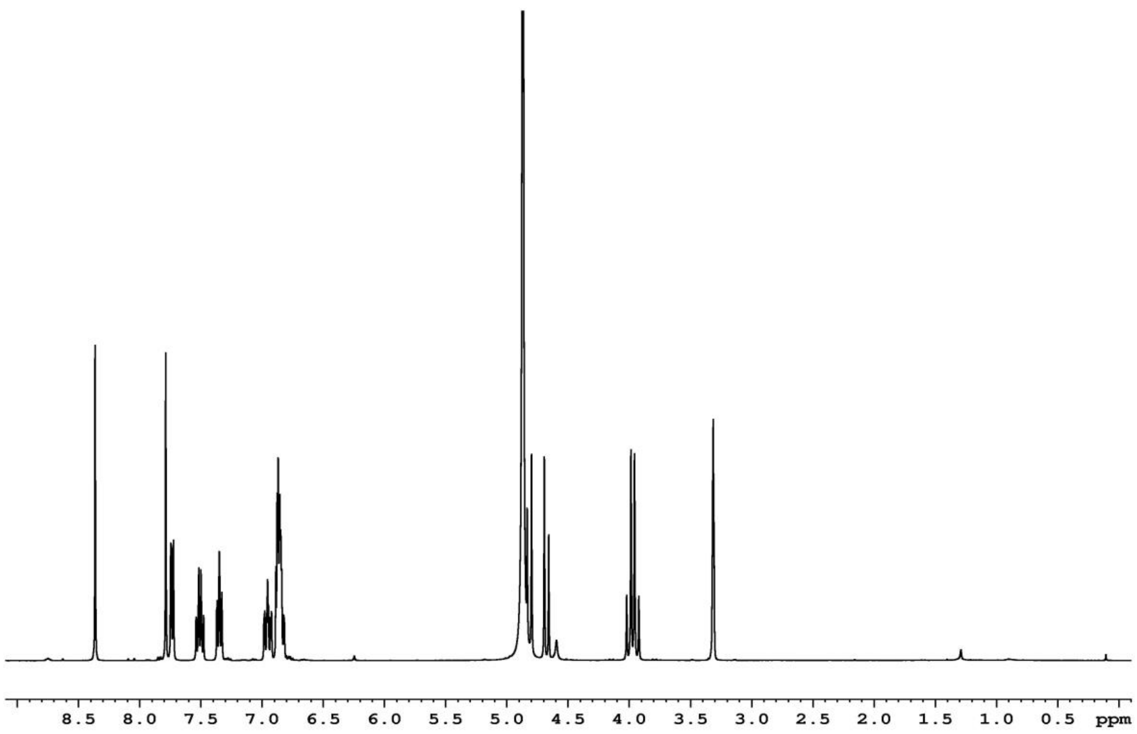

Figure S88. 400 MHz ^{1}H-NMR spectrum of hybrid **21** in CD_3OD at 298 K.

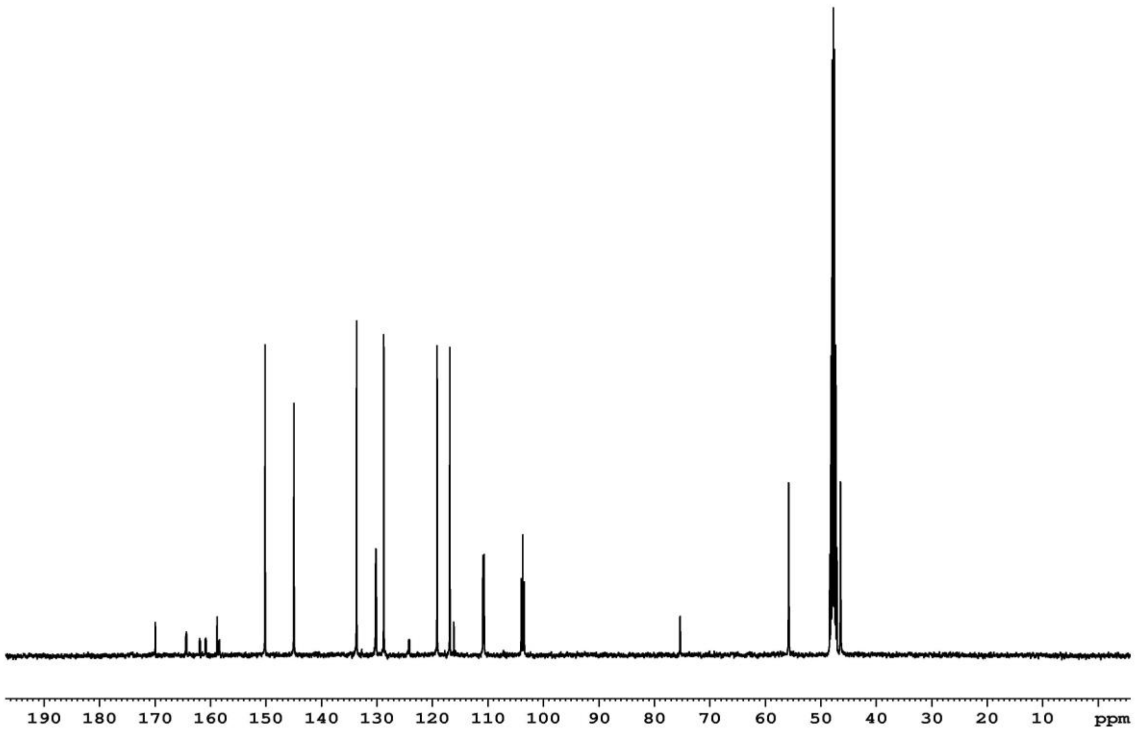

Figure S89. 100 MHz ^{13}C-NMR spectrum of hybrid **21** in CD_3OD at 298 K.

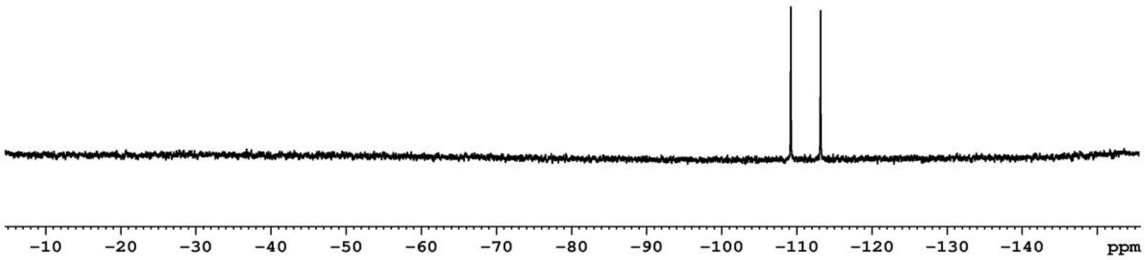

Figure S90. 375 MHz ^{19}F-NMR spectrum of hybrid **21** in CD_3OD at 298 K.

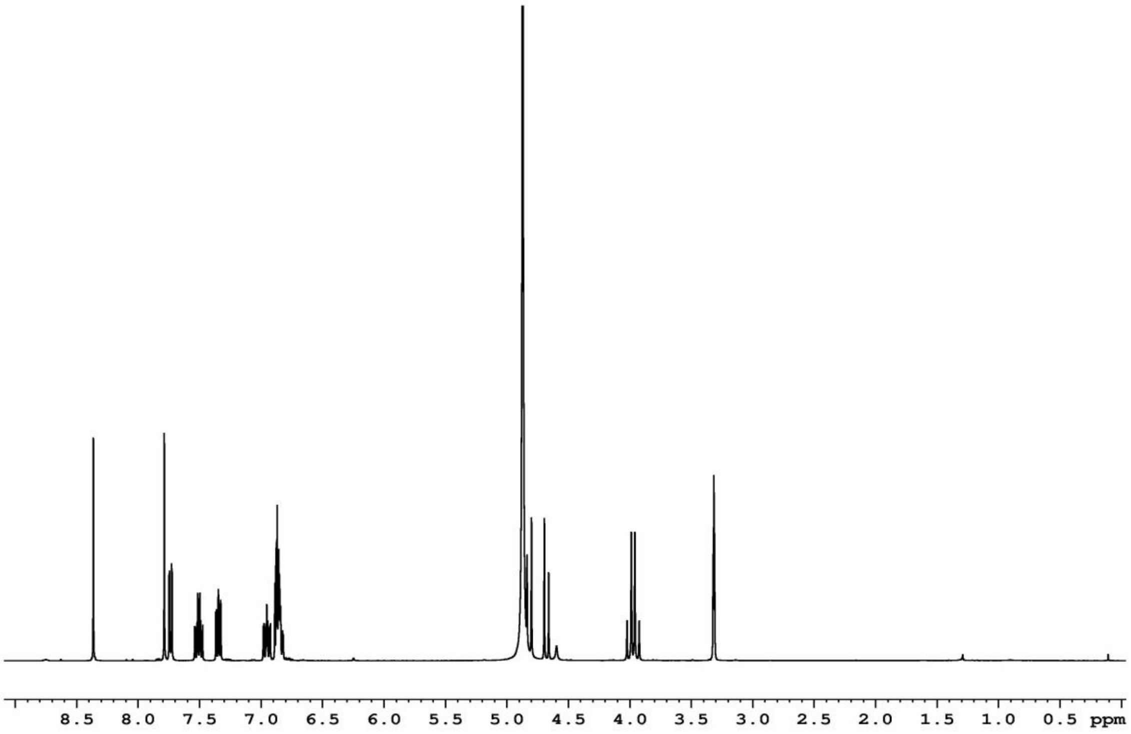

Figure S91. 400 MHz ^{1}H-NMR spectrum of hybrid **22** in CD_3OD at 298 K.

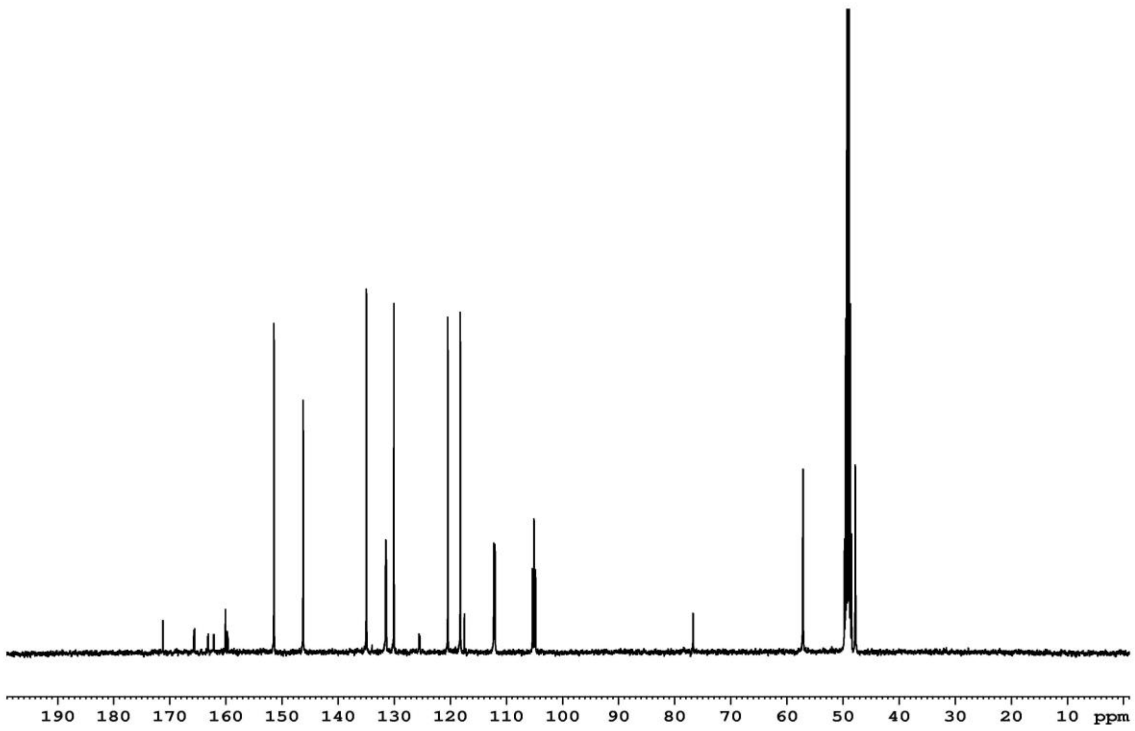

Figure S92. 100 MHz ^{13}C-NMR spectrum of hybrid **22** in CD_3OD at 298 K.

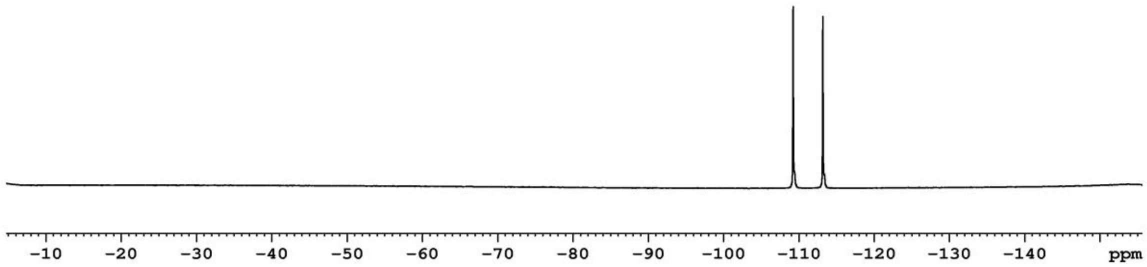

Figure S93. 375 MHz ^{19}F-NMR spectrum of hybrid **22** in CD_3OD at 298 K.

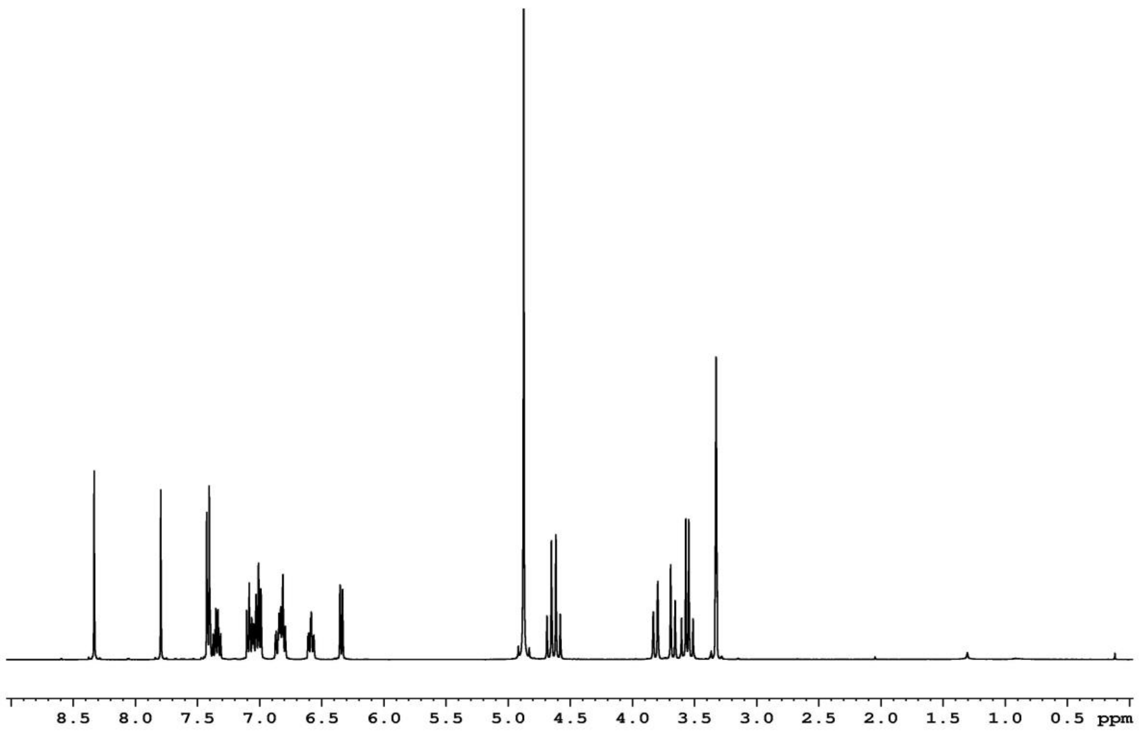

Figure S94. 400 MHz ^{1}H-NMR spectrum of hybrid **23** in CD_3OD at 298 K.

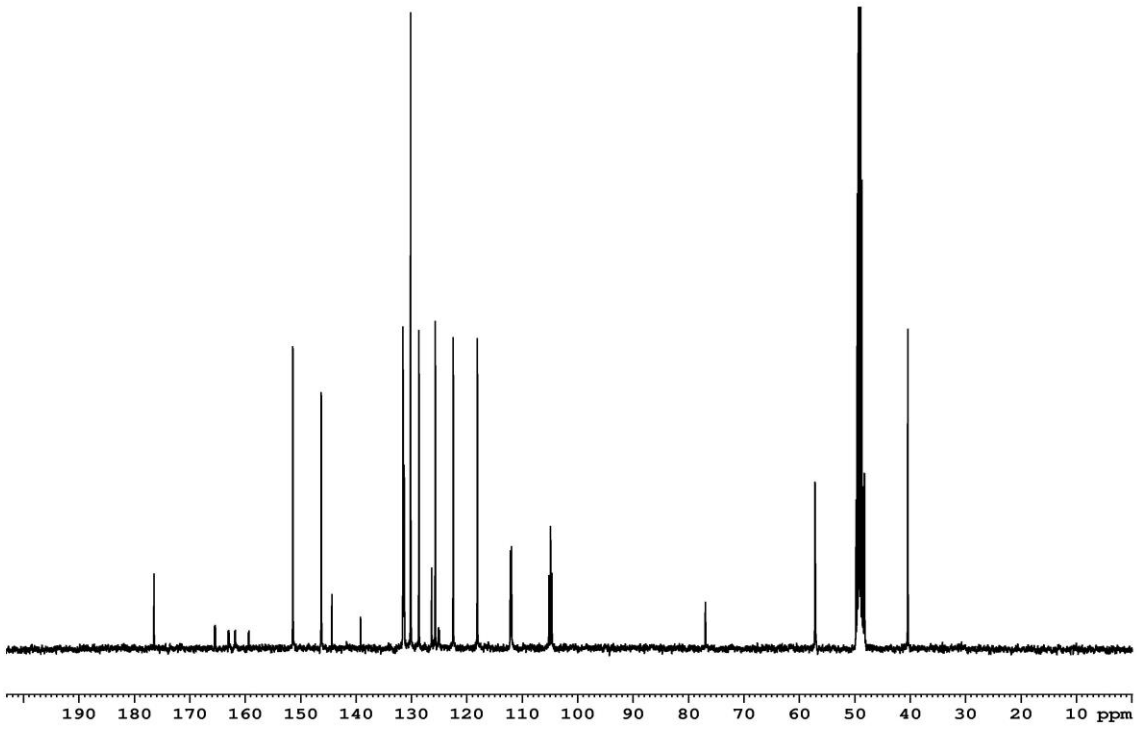

Figure S95. 125 MHz ^{13}C-NMR spectrum of hybrid **23** in CD_3OD at 298 K.

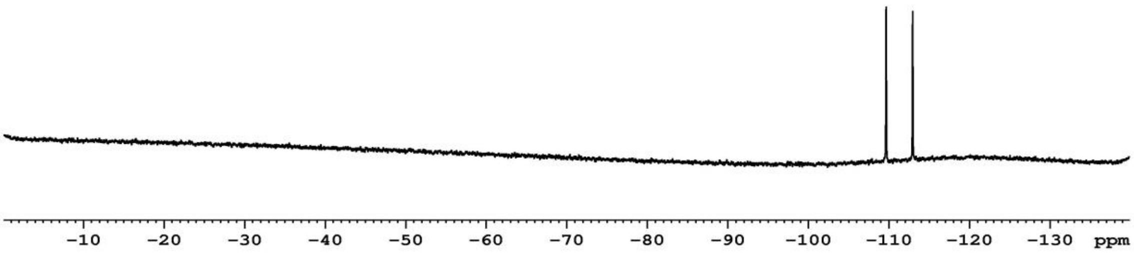

Figure S96. 375 MHz ^{19}F-NMR spectrum of hybrid **23** in CD_3OD at 298 K.

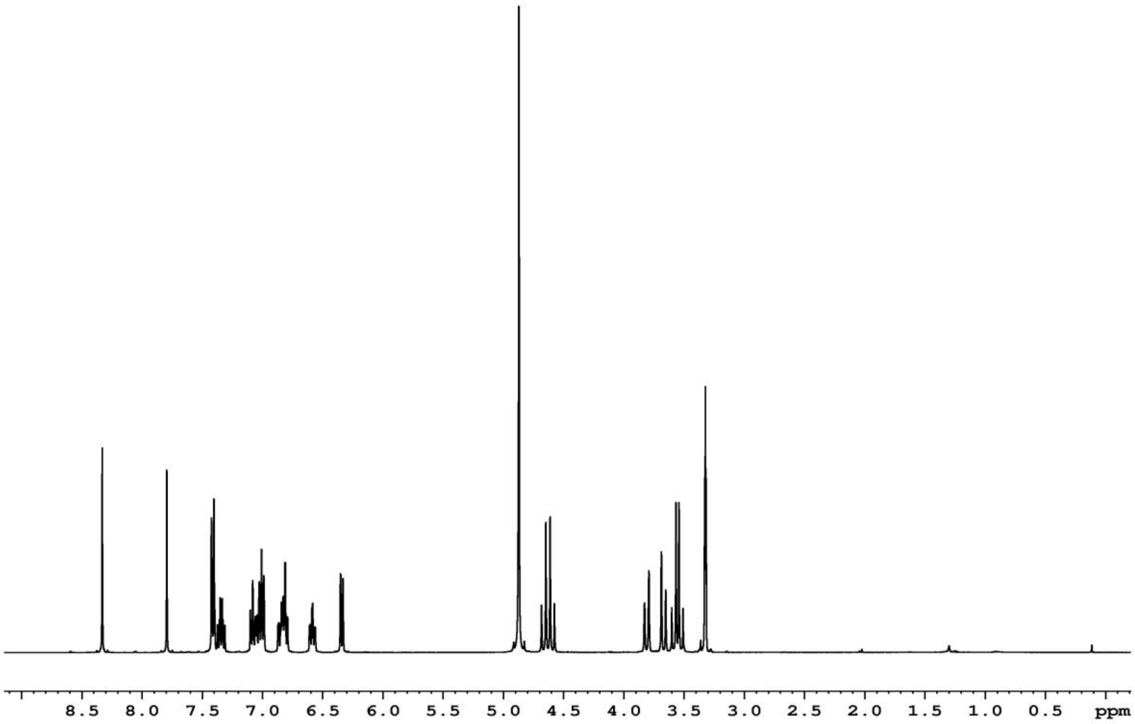

Figure S97. 400 MHz ^{1}H-NMR spectrum of hybrid **24** in CD_3OD at 298 K.

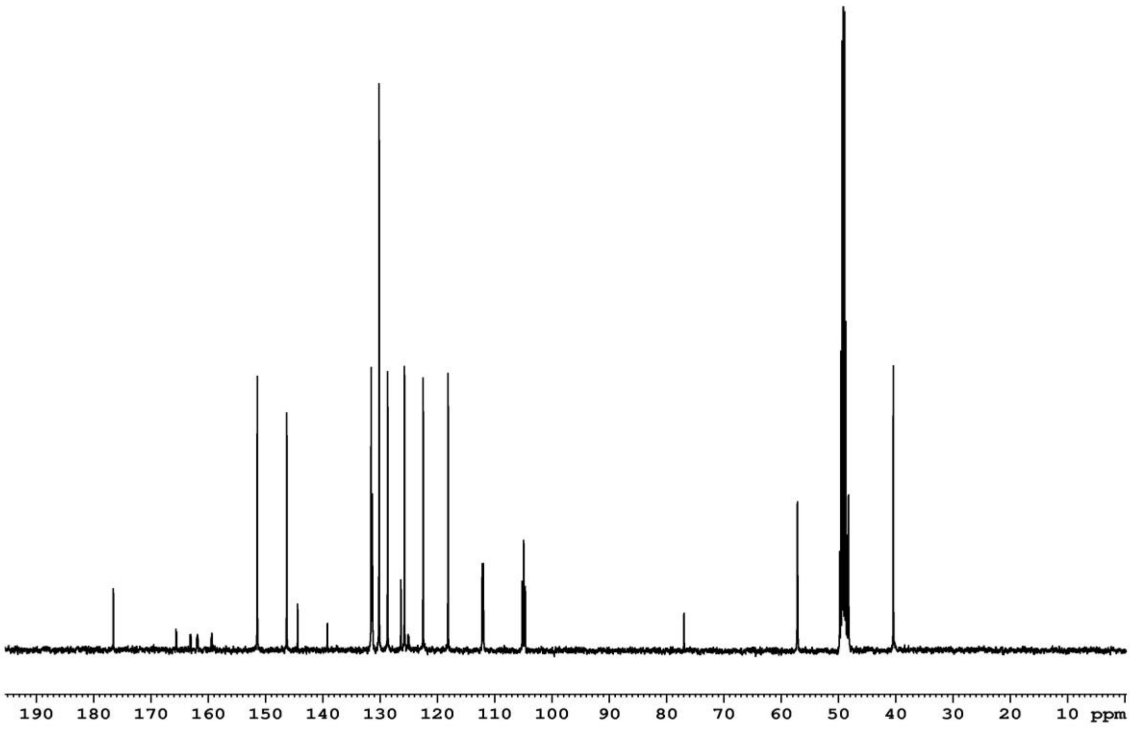

Figure S98. 100 MHz ^{13}C-NMR spectrum of hybrid **24** in CD_3OD at 298 K.

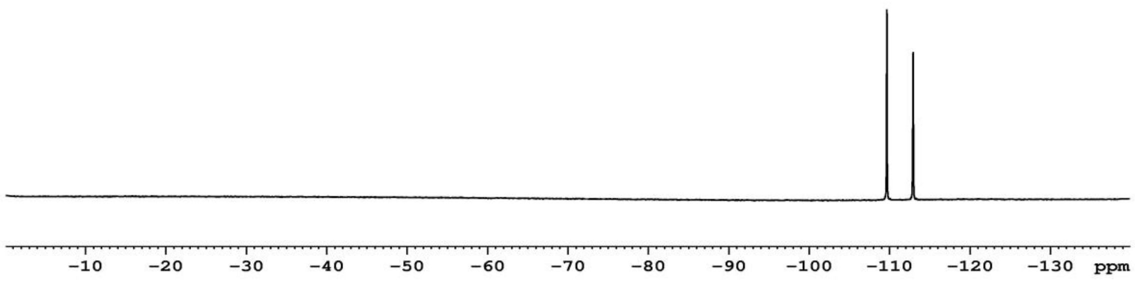

Figure S99. 375 MHz ^{19}F-NMR spectrum of hybrid **24** in CD_3OD at 298 K.

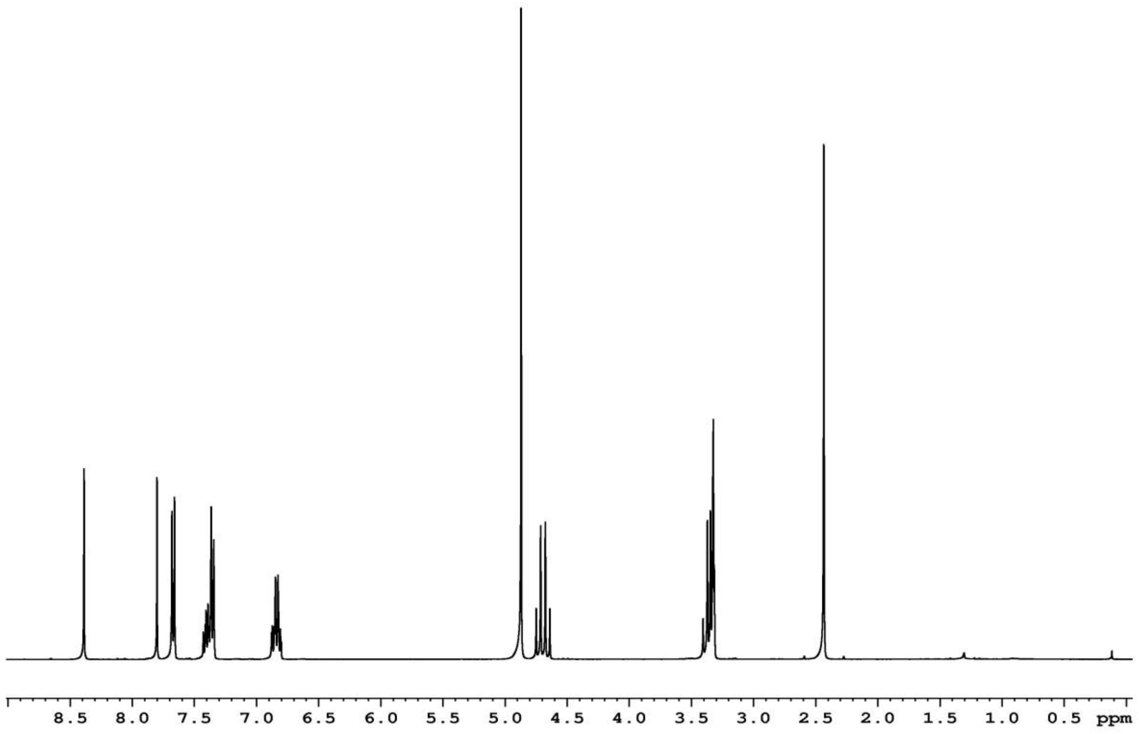

Figure S100. 400 MHz ^{1}H-NMR spectrum of compound **N-tosyl azole (*S*)** in CD_3OD at 298 K.

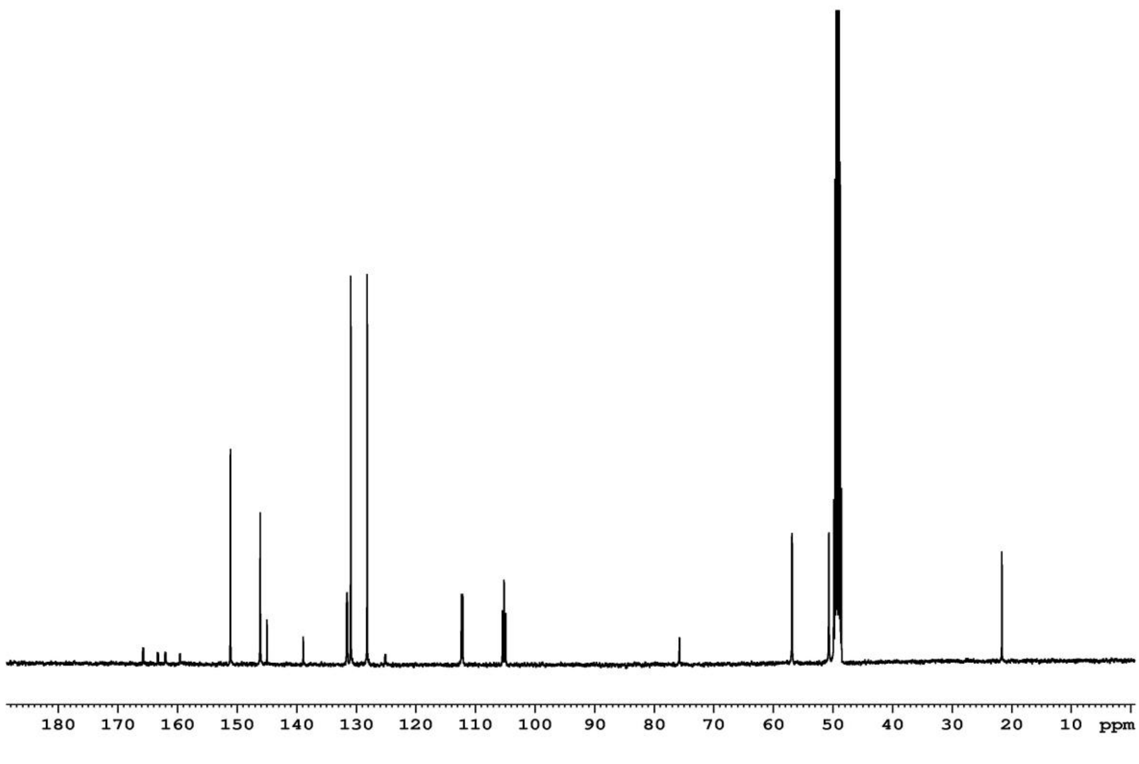

Figure S101. 100 MHz ^{13}C-NMR spectrum of compound **N-tosyl azole (*S*)** in CD_3OD at 298 K.

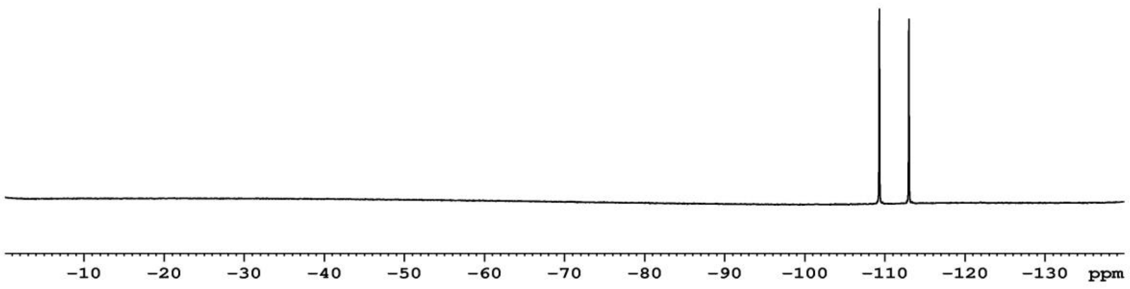

Figure S102. 375 MHz ^{19}F-NMR spectrum of compound **N-tosyl azole (*S*)** in CD_3OD at 298 K.

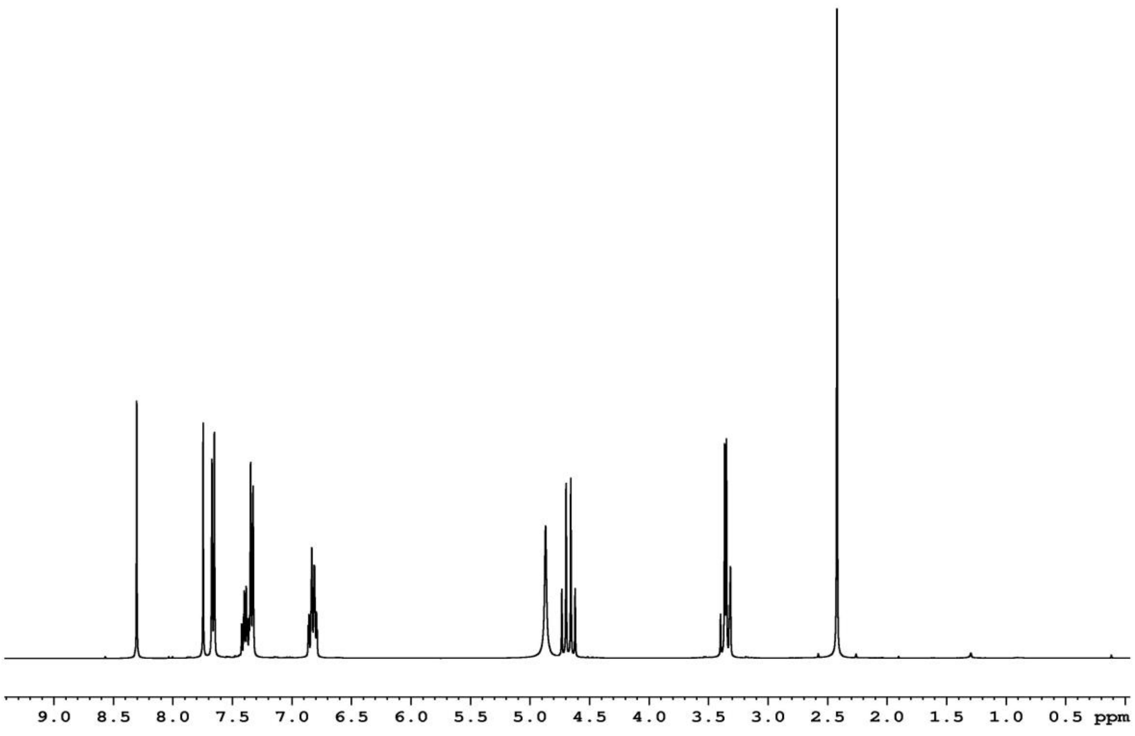

Figure S103. 400 MHz ^{1}H-NMR spectrum of compound **N-tosyl azole (*R*)** in CD_3OD at 298 K.

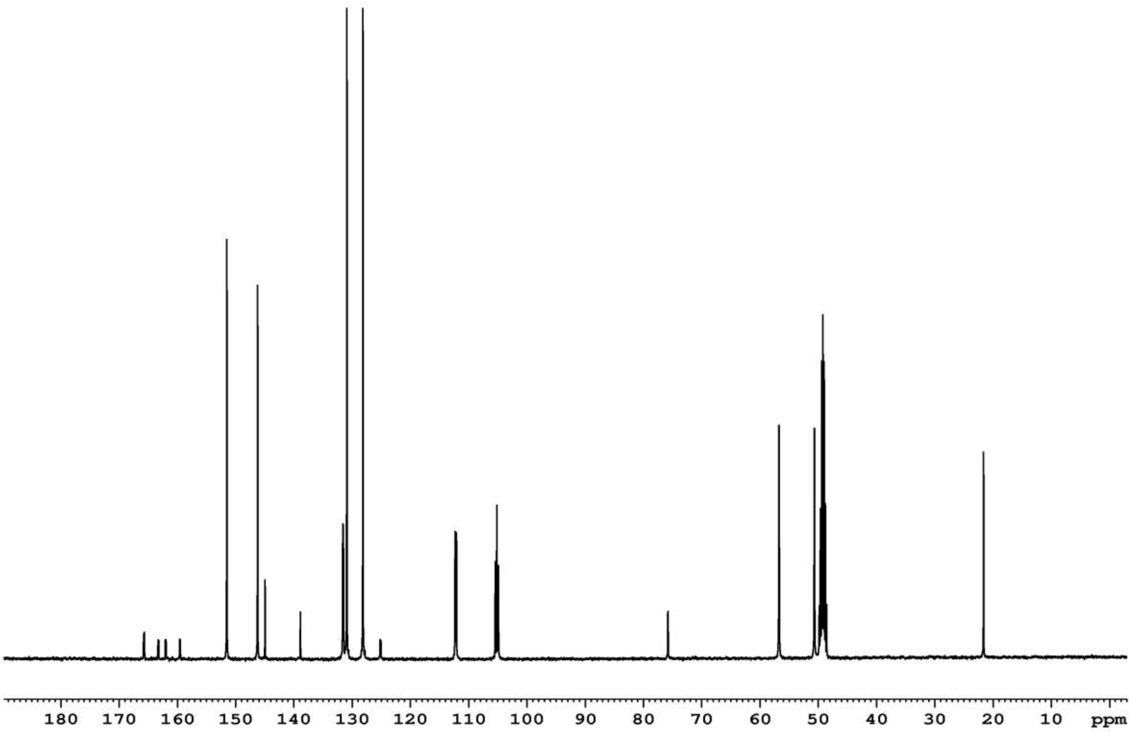

Figure S104. 100 MHz ^{13}C-NMR spectrum of compound **N-tosyl azole (*R*)** in CD_3OD at 298 K.

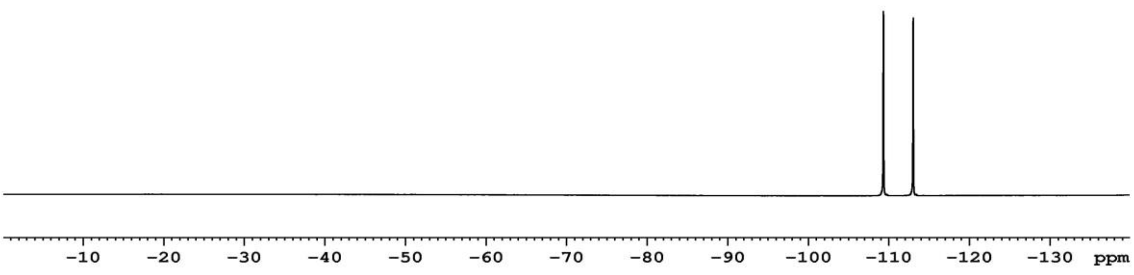

Figure S105. 375 MHz ^{19}F-NMR spectrum of Compound **N-tosyl azole (*R*)** in CD_3OD at 298 K.
